# A Novel Antimalarial Agent that Inhibits Protein Synthesis in *Plasmodium falciparum*

**DOI:** 10.1002/anie.202514085

**Published:** 2025-10-20

**Authors:** Patricia Bravo, Eleonora Diamanti, Mostafa M. Hamed, Lorenzo Bizzarri, Natalie Wiedemar, Armin Passecker, Nicolas M. B. Brancucci, Anna Albisetti, Christin Gumpp, Boris Illarionov, Markus Fischer, Matthias Witschel, Tobias Schehl, Hannes Hahne, Pascal Mäser, Matthias Rottmann, Anna K. H. Hirsch

**Affiliations:** ^1^ Medical Parasitology and Infection Biology Swiss Tropical and Public Health Institute Kreuzstrasse 2 Allschwil 4123 Switzerland; ^2^ Universität Basel Petersplatz 1 Basel 4003 Switzerland; ^3^ Helmholtz Institute for Pharmaceutical Research Saarland (HIPS)─Helmholtz Centre for Infection Research (HZI) PharmaScienceHub Campus Building E8.1 66123 Saarbrücken Germany; ^4^ OmicScouts GmbH Lise‐Meitner‐Straße 30 D‐85354 Freising Germany; ^5^ Hamburg School of Food Science Institute of Food Chemistry Grindelallee 117 20146 Hamburg Germany; ^6^ BASF‐SE Carl‐Bosch‐Strasse 38 67056 Ludwigshafen Germany; ^7^ Department of Pharmacy Helmholtz Institute for Pharmaceutical Research Saarland (HIPS)─Helmholtz Centre for Infection Research (HZI) Saarland University PharmaScienceHub Campus Building E8.1 66123 Saarbrücken Germany; ^8^ PharmaScienceHub Campus A 2.3 66123 Saarbrücken Germany

**Keywords:** 2‐hydroxyphenyl benzamides, Antimalarial, Cytosolic ribosomes, Drug discovery, Drug resistance

## Abstract

The emergence of drug resistance to nearly all antimalarials following their rollout underscores the need for novel chemotypes with novel modes of action to replenish the antimalarial drug‐development pipeline. We identified a novel class of compounds in the antimalarial armory. Compound **31**, characterized by a 2‐hydroxyphenyl benzamide scaffold, displays potent activity against blood‐stage and mature sexual stages of *Plasmodium falciparum* and no toxicity in human cells. Resistance selection studies with **31** identified a previously unknown point mutation in the *P. falciparum* multidrug‐resistance protein 1 (*pfmdr1*) gene, for which we confirmed causality by CRISPR/Cas9‐based gene editing as the primary mediator of resistance. No cross‐resistance toward first‐line antimalarials was identified in compound **31**‐resistant parasites. Proteomics studies indicated that the primary mode of action of **31** is through direct binding to cytosolic ribosomal subunits, thereby inhibiting protein synthesis in the parasite. Taken together, compound **31** is a promising starting point for the development of a next‐generation antimalarial.

## Graphical abstract

Compound **31** represents a promising avenue for the development as an antimalarial agent with a potent activity profile (*Pf*NF54 IC_50 _± s.d. = 3.9 ± 0.4 nM). It has a novel mode of action by binding to cytosolic ribosomal subunits, thereby inhibiting protein synthesis in the parasite.

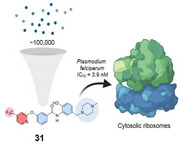

## Introduction

Malaria remains a major threat in global health.^[^ ^1^ ^]^ The impact of this disease led to an estimated annual loss of 600,000 lives and US $4.0 billion in 2023 for malaria control and elimination, slowing economic growth particularly in sub‐Saharan Africa, where 95% of the cases occur.^[^ ^1^ ^]^ Malaria cases rose significantly, with 11 million more estimated cases in 2023 than the previous year.^[^ ^1^ ^]^ Aside from disruptions in health services due to humanitarian emergencies and economic crises, resistance to insecticides and current antimalarial treatments remain a major concern. Artemisinin combination therapy (ACT) has been adopted worldwide as first‐line treatment for uncomplicated malaria caused by *Plasmodium falciparum*.^[^ ^2^ ^]^ In severe cases, ACT is administered after initial treatment with parenteral artesunate.^[^ ^2^ ^]^ However, their short half‐lives and the emerging drug resistance toward artemisinins and partner drugs underlines the need to refill the drug‐development pipeline with new chemotypes having novel mode of action as parasites acquire resistance toward older drugs.^[^ ^3^, ^4^, ^5^ ^]^

**Figure 1:**
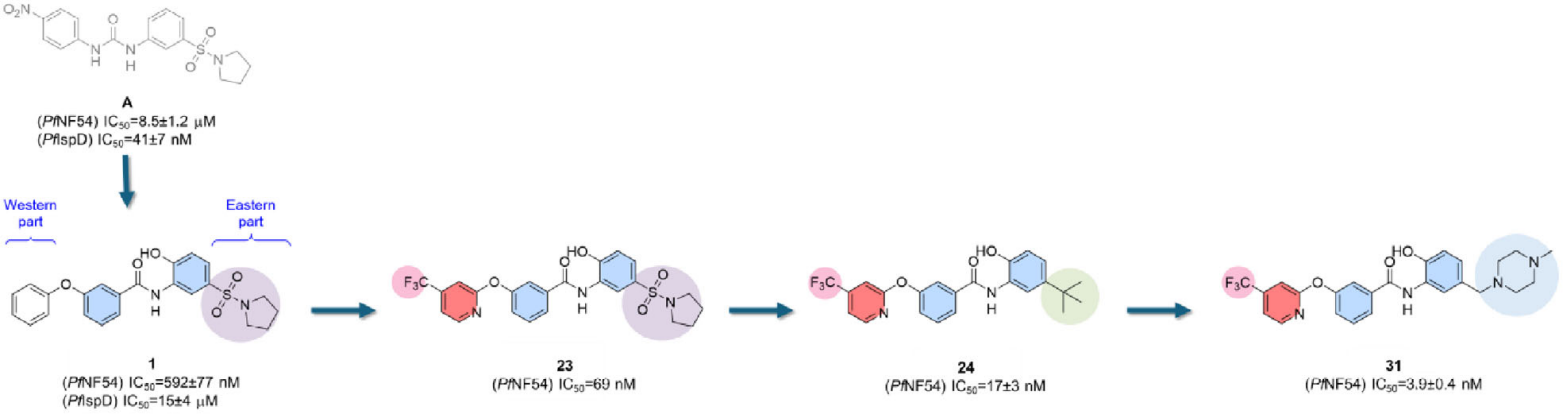
Chemical evolution of **31**. Data are expressed as mean IC_50_ ± standard deviation (s.d.).

A high‐throughput target‐based screening (HTS) of the BASF proprietary library, consisting of ∼1,(00,000 compounds, led to the identification of a novel chemical class targeting the third enzyme (*Pf*IspD) of the methyl‐D‐erythritol phosphate (MEP) pathway in *P. falciparum* (compound **A**, Figure 1). Extensive exploration of related scaffolds resulted in the identification of compound **1**, which showed potent activity against the *P. falciparum* NF54 strain but with only weak inhibition of *Pf*IspD, suggesting a distinct mode of action.^[^ ^6^ ^]^ Extensive phenotypic optimization of this series led to the complete loss of *Pf*IspD activity but resulted in single digit 50% inhibitory concentration (IC_50_) in the NF54 strain with compound **31** emerging as the frontrunner of this study (*Pf*NF54 IC_50 _= 3.9 nM). Here, we describe a series of experiments including in vitro resistance selection, CRISPR/Cas9 genetic validation, *in cellula* translation experiments and proteomics in the search of the mode‐of‐action (MoA) of compound **31** in *P. falciparum*.

## Results and Discussion

### Hit Optimization

Compound **1** features a 2‐hydroxyphenyl benzamide scaffold and a mean *Pf*NF54 IC_50 _± s.d. = 592 ± 77.6 nM but has lost activity against *Pf*IspD (**1,** IC_50 _± s.d. = 15 ± 4 µM). With the aim to identify novel and potent antimalarial compounds, the study was continued with a focus on *Pf*NF54 cell activity. The experimental details of the synthesis are outlined in the (Schemes S1–S10) along with the *Pf*NF54 IC_50_ values and predicted physiochemical properties of all the compounds (**1**–**31**, Table S1).

Initially, a small subset of derivatives was designed and synthesized and found that methylation of the phenolic ‐OH (**2**, IC_50_ > 10,000 nM), of the nitrogen atom of the amide function (**4**, IC_50_ > 10,000 nM), or both in parallel (**3**, IC_50_ > 10,000 nM) led to a complete loss of *P. falciparum* potency. A similar trend was observed for the complete omission of the phenolic ─OH (**5**, IC_50_ ± s.d. = 4927 ± 97.8 nM ).

The Eastern part of the molecule was then explored with various modifications, including the removal of the pyrrolidine to have a free sulfonamide (**6**, IC_50_ ± s.d. = 777 ± 86 nM), the use of linear alkyl chains as in **7** IC_50_ ± s.d. = 217 ± 42.4 nM) and (**8**, IC_50_ ± s.d. = 1910 ± 254 nM) or the swapping from a 5‐ to a 6‐membered ring as in (**9**, IC_50_ ± s.d. = 574 ± 66.53 nM). We were also interested to see how alkylation of the aliphatic rings (**10**, IC_50_ ± s.d. = 1409 ± 147 nM and **11**, IC_50 _± s.d. = 1026 ± 116 nM), introduction of a morpholine ring (**12**, IC_50_ ± s.d. = 197 ± 28 nM) or of a benzylic moiety (**14**, IC_50_ ± s.d. = 2783 ± 389 nM) might affect the activity. Overall, these modifications resulted in either similar or decreased activity (Table S1). The pyrrolidine ring was then kept constant, and the orientation of the phenoxy benzene ring was shifted from the *meta* to the *para* position (**15**, IC_50_ ± s.d. = 539 ± 92.15 nM), which resulted in a slight decrease in potency. Accordingly, by retaining the *meta* orientation, a subset of molecules was made with variable substituents (**16**–**18**) and heterocyclic rings (**19**–**23**), which resulted in compound (**23**, IC_50_ = 69 nM) with an about 8‐fold increased activity compared to the initial hit molecule **1**.

Because of this pattern, the focus was shifted back to the Eastern region and found that the sulfonamide function could be successfully replaced by an alkyl moiety (**24**, IC_50 _± s.d. = 17 ± 3 nM and **25**, IC_50 _± s.d. = 34 ± 4 nM) or a triazole ring (**26**, IC_50 _± s.d. = 158 ± 15.6 nM). Despite an increase in potency, however, compound **24** suffered from poor physicochemical properties (Table 1). Therefore, the chemical optimization of the drug‐like properties was pursued with a subset of compounds such as (**27**, IC_50 _± s.d. = 11 ± 3.4 nM), (**28**, IC_50 _± s.d. = 41 ± 17.2 nM), (**29**, IC_50 _± s.d. = 7.6 ± 1.8 nM), (**30**, IC_50 _± s.d. = 10 ± 1.8 nM), and (**31**, IC_50 _± s.d. = 3.9 ± 0.4 nM), investigating different alkyl amine chains. The focus was then shifted both to the central ring and to the amidic linker. In this direction, compounds (**32,** IC_50 _± s.d. = 21.6 ± 2.65 nM and **33,** IC_50 _± s.d. = 63 ± 7.4 nM) were synthesized by a multistep route, bearing a fluorine and a chlorine, respectively, next to the phenolic ─OH (Scheme S7). Then, the phenolic OH was replaced by a fluorine or by the nitrogen of the pyridyl ring (**35,** IC_50 _> 10,000 nM) (Scheme S8) and, in parallel, the amide linker was investigated by introducing a methylene (**36**, IC_50 _± s.d. = 808 ± 172 nM, Scheme S9) and by making a triazole ring as a bioisostere of the amide (**37**, IC_50 _± s.d. = 21 ± 1.13 nM, Scheme S10).

**Table 1:** Profiling of compounds **24**, **30**–**33**.

Code	*c*LogD^a)^	t_1/2_ [min] Mouse Liver S9	HepG2 %inhibition@ 100 µM
24	4.78	5.4 ± 0.5	n.d.
30	2.82	28.1 ± 5.5	45 ± 21
31	2.45	42.7 ± 3.7	87 ± 5 (CC_50_ = 37.0 ± 1.0 µM)^b)^
32	2.89	18.4 ± 4.2	51 ± 1
33	3.35	49.9 ± 4.0	66 ± 2

n.d. = not determined;

^a)^ Calculated logarithim of the distribution coefficient (*c*LogD) calculated with Startdrop version 7.0.1.29911;

^b)^ Data are expressed as mean CC_50_ ± s.d.

Among the series, compound **31** was identified as the frontrunner with the best‐balanced profile considering potency and the criteria listed in Table 1. To assess cytotoxicity, **31** was tested against human liver cell line HepG2, which exhibited a 50% cytotoxic concentration (CC_50 _± s.d.) of 37 ± 1.0 µM (Table 1). Given that it had an IC_50_ of 3.9 nM against *P. falciparum*, the selectivity index (CC_50_/IC_50_) of **31** was 9,487.

### Compound 31 is Active Against Stage V Gametocytes and Drug‐Resistant *P. falciparum* Strains

Compound **31** was also found to be active against the sexual stage of *P. falciparum* using the MitoTracker‐based readout. This readout is based on quantifying MitoTracker‐based gametocyte viability as recently described.^[^ ^7^ ^]^ Briefly, high content imaging of MitoTracker‐ and Hoechst‐stained cells and automated image analysis using the MetaXpress software was used to determine the number of viable gametocytes based on mitochondrial activity and cellular shape. The IC_50_ value for mature (stage V) gametocytes was 354.5 nM (*N* = 3, Fig S5). While there is a 91‐fold reduction in the activity against the sexual stages compared to the asexual stage, **31** is still effective against the mature gametocytes at sub‐micromolar concentrations, demonstrating transmission‐blocking potential.

To determine potential cross resistance against compound **31** in parasites harboring mutations that confer resistance to current antimalarials, its activity was assessed against drug‐resistant field isolates as previously described. Compound **31** was tested against *P. falciparum* K1 and Dd2 strains that were resistant to chloroquine, pyrimethamine, and sulfadoxine (Table S2).^[^ ^8^ ^]^ A cross‐resistance was considered when there was more than a 10‐fold shift in the IC_50_ relative to the NF54 strain. While no shift in the IC_50_ was observed against the K1 strain when compared to the NF54 strain, an elevated IC_50_ value was observed against the Dd2 strain (IC_50_ ± s.d. = 9.3 ± 0.4 nM). Because of this, **31** was tested against laboratory‐selected Dd2 strains, which contained additional resistance variants to preclinical and clinical drug candidates. These included DDD107458 (protein synthesis inhibitor through mutations targeting the translation elongation factor 2 (*pfeEF2*)^[^ ^9^ ^]^), MMV390048 (phosphatidylinositol 4‐kinase inhibitor (*pfpi4k*)^[^ ^10^ ^]^), GNF156 (cyclic amine resistance locus(*pfcarl*)^[^ ^11^ ^]^), NITD609 (P‐type ATPase 4 (*pfatp4)* ^[^ ^12^ ^]^), ELQ300 (cytochrome bc1 complex inhibitor (*pfcytb*),^[^ ^13^ ^]^ DSM265 (dihydroorotate dehydrogenase inhibitor (*pfdhodh*)^[^ ^14^ ^]^), and fosmidomycin (1‐deoxy‐D‐xylulose‐5‐phosphate reductoisomerase inhibitor (*pfdxr*)^[^ ^15^ ^]^). As no evidence of reduced potency against the laboratory‐selected Dd2 strains (IC_50_ ranging from 2.4–6.6 nM; Table S2) and no shift in the IC_50_ in all the strains relative to the parent Dd2 strain were found, we conclude that **31** is not targeting these MoAs.

### Resistance to 2‐hydroxyphenyl Benzamides is Mediated by Mutations in *pfmdr1*

In vitro resistance evolution coupled with whole‐genome sequencing (WGS) is one of the most successful ways to gain insights into the antimalarial MoA.^[^ ^16^ ^]^ In an attempt to find the MoA, the frontrunner **31** was used to apply compound pressure to a culture of Dd2b2, a strain with an accelerated mutation rate.^[^ ^17^, ^18^ ^]^

**Figure 2:**
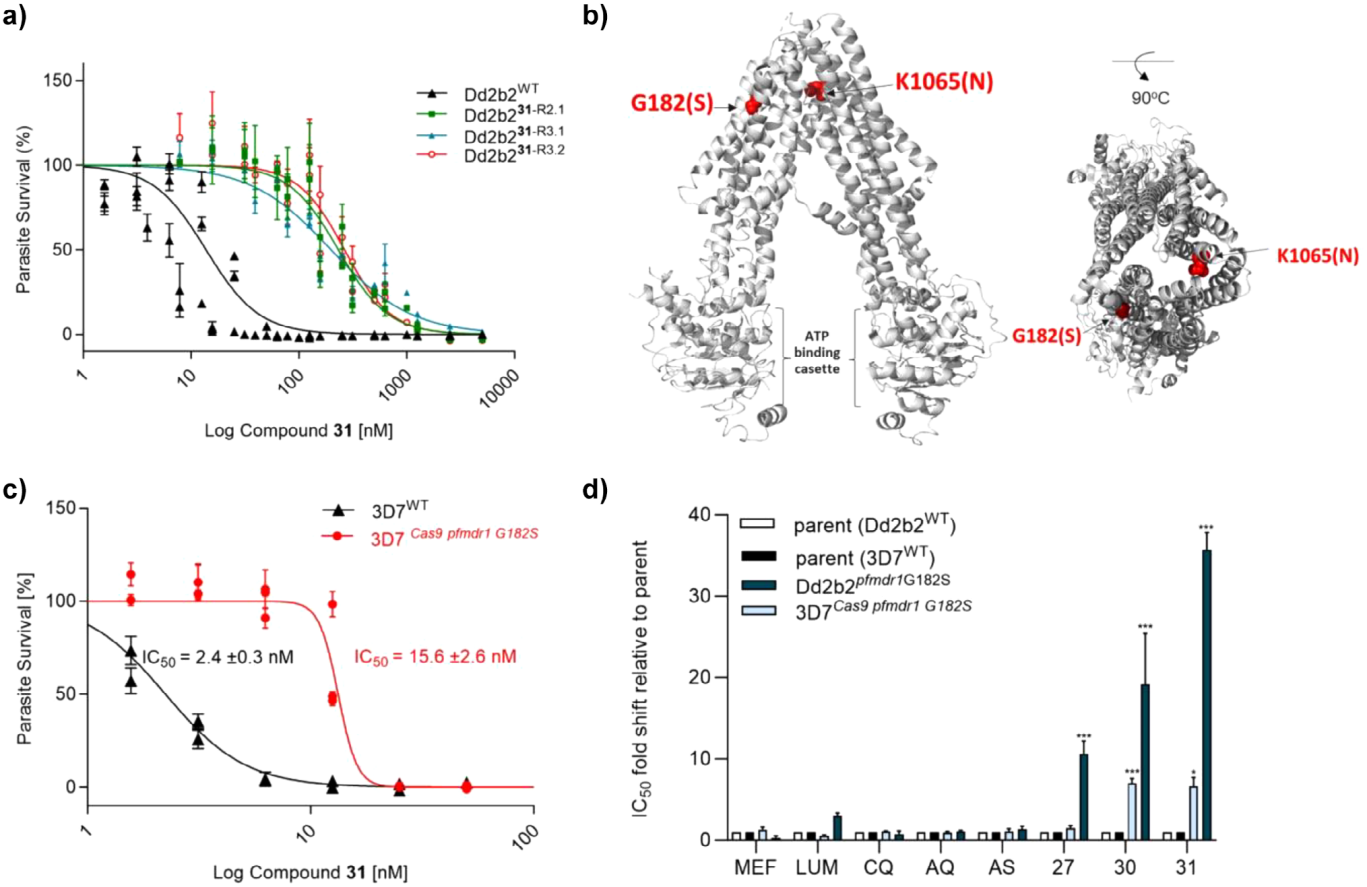
Mutations in PfMDR1 are responsible for the parasite`s resistance to compounds **27**, **30,** and **31**. **a)** Concentration‐response curves of the three clones with the highest IC_50_ fold shift relative to the parent clone. Graph shows that fold shifts within resistant clones (*Dd2b2* **^31^** ^‐R2.1^, *Dd2b2* **^31^** ^‐R3.1^, and *Dd2b2* **^31^** ^‐R3.2^) are in the similar range. Data are expressed as mean IC_50_ ± s.d. Three independent assays were performed. **b)** Cryo‐EM structure of PfMDR1(PDB ID 8jvh). The ATP binding cassette is located on the cytosolic side. The amino acid residues where mutations were identified are represented as red spheres and are located within the inner pore of the *Pf*MDR1 close to the digestive vacuole side. The right panel shows the same structure rotated 90° for spatial context. **c)** Parasites Cas9‐edited to express the *pfmdr1* carrying the G182S point mutation (3D7^Cas9^ *^pfmdr1 G182S^*) shows resistance to **31**. Data are expressed as mean IC_50_ ± s.d. Three independent biological experiments were performed. **d)** *pfmdr1* ^G182S^ mutation in both Cas9‐edited clone (3D7^Cas9^ *^pfmdr1 G182S^*) and **31**‐pressured clone (Dd2b2*^pfmdr1 G182S^*) confer *P. falciparum* resistance to 2‐hydroxyphenyl benzamides (**27**, **30**, and **31**) and does not reduce sensitivity to standard antimalarials mefloquine (MEF), lumefantrine (LUM), chloroquine (CQ), amodiaquine (AQ), and artesunate (AS). Statistical analysis was done with a student`s *t*‐test to compare resistant to parental clones; ***P–value <0.001, **P–value < 0.002, and *P–value < 0.033; *N* = 3, two biological replicates.

Three independent cultures were exposed to sublethal concentrations of **31**. After 19 days of compound pressure, the IC_50_ values had increased to 14‐ to 20‐fold for parasites selected with **31** relative to the parental clone (Figure 2a; Tables S3–S4). These selected clones were also resistant to close analogues of **31**, with 9‐ to 19‐ fold increased IC_50_ values (**27** and **30**, Table S7).

To determine the molecular basis of the in vitro resistance against **31**, genomic DNA (gDNA) of the three clones with the highest IC_50_ fold shift (Dd2b2**^31^** ^−R2.1^, Dd2b2**^31^** ^−3.1^, and Dd2b2**^31^** ^−3.2^) and the parent strain (Dd2b2^WT^, from day 0 of the selection experiments) was isolated and sequenced on the Illumina platform. Mapping of the sequencing reads to a *P. falciparum* reference genome identified shared mutations of two genes in the resistant clones, which were not present in the parental clone: PF3D7_0523000, which encodes for the multidrug resistance protein 1 (*pfmdr1*), and PF3D7_0305500, which encodes for the protein dopey homolog (*pfdopey*). All three clones carried heterozygous mutations in *pfmdr1* (G182S was present in all resistant clones and K1065N in clone Dd2b2**^31^** ^−3.1^) (Figure 2b and S1A,B). Further, all three resistant clones had a conservative in‐frame insertion (N3349 duplication) in *pfdopey* (Table S6, Figure S1C). These mutations were confirmed by Sanger sequencing except for the G182S mutation in *pfmdr1* for the clone Dd2b2**^31^** ^−3.1^.

To validate the causal role of the *pfmdr1* ^G182S^, *pfmdr1* ^K1065N^, and *pfdopey* ^N3449^ variants in *P. falciparum* resistance to **31**, a CRISPR/Cas9 system was utilized. We attempted to individually introduce the nucleotide substitutions leading to G182S and K1065N in *pfmdr1* (PF3D7_0523000) and the insertion leading to the N3449 duplication in *pfdopey* (PF3D7_0305500).

No mutants could be generated for parasites transfected with the *pfmdr1* ^K1065N^ and *pfdopey* ^N3449^ variants despite the use of three different single guide RNAs (sgRNAs). While the reason for this failure is unknown, a possible explanation could be that the individual introduction of *pfmdr1* ^K1065N^ and *pfdopey* ^N3449dup^ resulted in nonviable parasites without the other compensatory mutations identified in the resistant clones. On the other hand, parasites harboring the *pfmdr1* ^G182S^ variant were successfully generated by incorporating the nonsynonymous mutation G182S.

Concentration‐response assay with **31** confirmed that the G182S mutation in the *pfmdr1* gene resulted in resistance to this chemical class, with a 7‐fold increase in the IC_50_ for compound **31** observed in the 3D7^Cas9^ *^pfmdr1 G182S^* clone compared to the 3D7 parental clone (IC_50 _± s.d. = 15.6 ± 2.3 nM, Figure 2c; Table S9). As expected, resistance was also observed to close analogues of **31**, with **27** and **30**, which resulted in a 2–7‐fold shift of the IC_50_ in the 3D7^Cas9^ *^pfmdr1 G182S^* clone relative to the parental clone (IC_50 _± s.d. = 3.3 ± 0.7 nM, IC_50 _± s.d. = 18.2 ± 0.9 nM, respectively, Table S9).

Previous studies have shown that polymorphisms in the *pfmdr1* gene are associated with varying susceptibility to ACT partner drugs.^[^ ^19^, ^20^, ^21^ ^]^ To explore whether the *pfmdr1* ^G182S^ mutation has an impact on the parasite sensitivity to ACT partner drugs, aryl amino alcohols such as mefloquine (MEF), lumefantrine (LUM), and 4‐aminoquinolines such as amodiaquine (AQ), chloroquine (CQ), and artesunate (AS) were tested in the 3D7^Cas9^ *^pfmdr1 G182S^* clone and the **31**‐selected clones (Dd2b2*^pfmdr G182S^*). No significant changes of the IC_50_ values were observed for these standard antimalarials in both 3D7^Cas9^ *^pfmdr1 G182S^* (Table S9) and Dd2b2*^pfmdr G182S^* clones (Table S7) relative to the parental clone. In addition, the same standard antimalarials were also tested on the Dd2b2*^pfmdr K1065N^* clone and showed no significant changes in the IC_50_ values obtained relative to the parental clone. This suggests that the mutations identified in *pfmdr1* do not reduce the parasite's susceptibility to these compounds.

Overall, these data suggest that new mutations identified in *pfmdr1* provide a resistance pathway in *P. falciparum* specific to 2‐hydroxyphenyl benzamides. This resistance mechanism does not reduce the parasite`s susceptibility to other antimalarials, allowing **31** to be a good partner drug for combination therapies.

PfMDR1 belongs to the ATP‐binding cassette (ABC) transporter superfamily and is an orthologue of the mammalian P‐glycoprotein 1 transporter.^[^ ^22^ ^]^ PfMDR1 is localized in the parasite`s digestive vacuole (DV) and is predicted to facilitate the import of solutes into the DV.^[^ ^23^ ^]^ The protein contains two symmetric parts, each with six transmembrane domains and a nucleotide‐binding domain (Figure S2). Interpro scan and topology model^[^ ^19^ ^]^ were used to predict the transmembrane regions of the protein where the mutations are located (Figure S2), while the resolved crystal structure of the PfMDR1 protein (PDB ID 8jvh) ^[^ ^24^ ^]^ was used to map the mutated amino acids (Figure 2b). The protein comprises two symmetric parts, each with six transmembrane domains and a nucleotide‐binding domain (Figure S2). The G182S mutation is located in the transmembrane region (TM) 3, whereas K1065N is located in TM12 (Figure S2). Both mutations are at the periphery close to the transporter's outer surface toward the DV (Figure 2b). TM3 is predicted to be involved in the protein`s transport kinetics into the DV whereas TM12 is predicted to be involved in the ligand‐binding of the protein.^[^ ^24^ ^]^ Indeed, a change to serine at position 182 might result in the accumulation of **31** in the DV, reducing its cytosolic concentration. Such a resistance pathway would point to a target in the cytosol. This sequestration mechanism is a model also proposed for the antimalarial MEF.^[^ ^19^, ^25^ ^]^ Although further functional characterizations at residue 182 are needed to determine transport kinetics and substrate specificity to PfMDR1, the identified mutation will be a useful molecular marker of drug resistance should **31** be used for further development en route to a new antimalarial agent.

### Compound 31 Inhibits Protein Synthesis in *P. falciparum*

To further elucidate the MoA of **31**, an integral solvent‐induced protein precipitation (iSPP) assay was employed as recently described.^[^ ^26^ ^]^ iSPP utilizes a solvent mixture (acetone/ethanol/acetic acid) to induce protein denaturation, allowing the detection of drug‐protein interactions by quantitative mass spectrometry based on changes in protein stability due to compound binding using cellular lysates from in vitro blood‐stage *P. falciparum* parasites.

**Figure 3:**
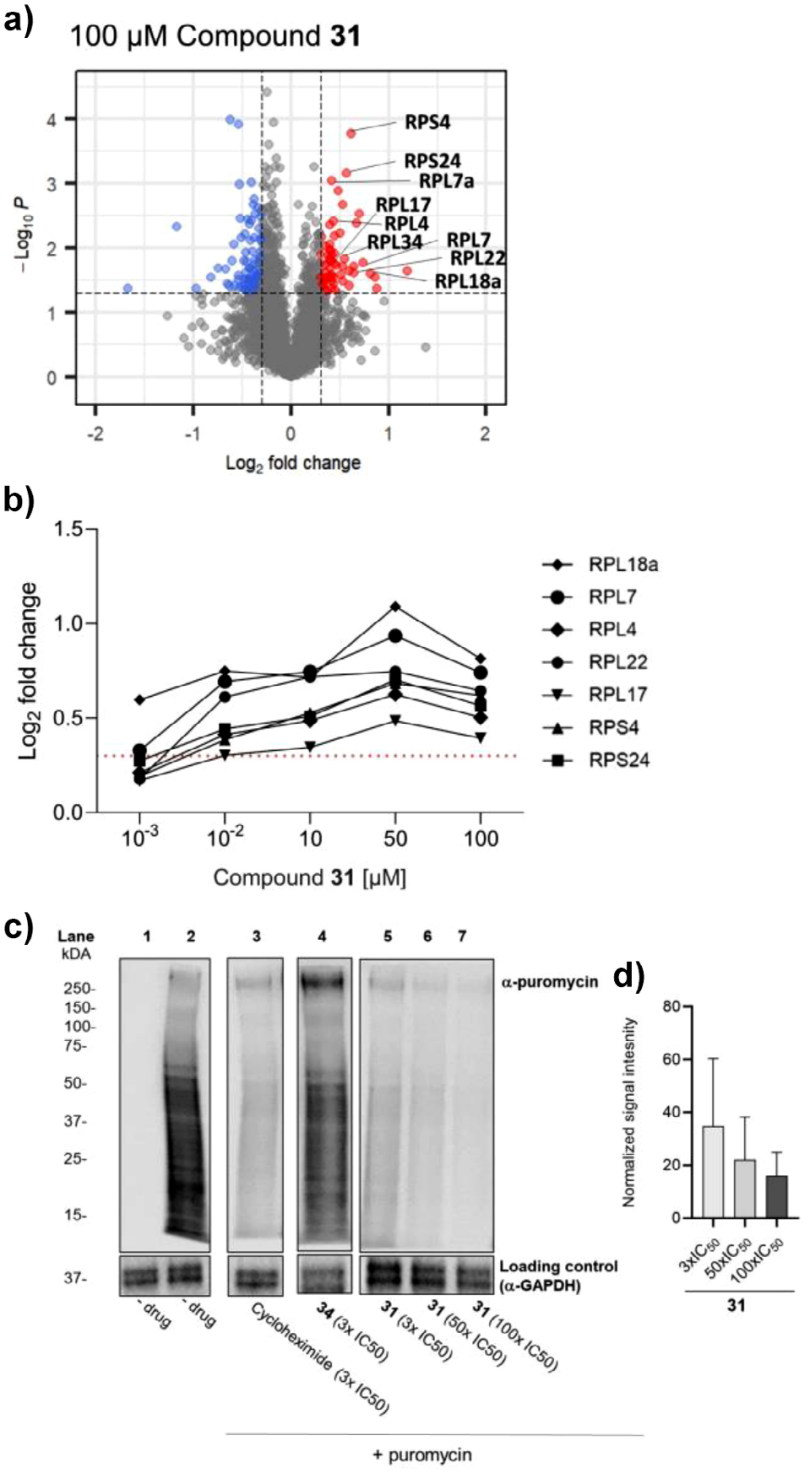
Compound **31** interacts with the cytosolic ribosomal complex and interferes with protein synthesis. **a)** Volcano plot from the integral solvent induced protein precipitation assay showing protein abundance changes in *Plasmodium falciparum* lysates incubated with **31** at 100 µM concentration compared to vehicle control. Out of the 104 (de) stabilized proteins, several ribosomal proteins (RP) were significantly stabilized. Data are shown as volcano plot where the threshold criteria for the identification of proteins exhibiting statistically significant changes in response to the compound treatment was set to a log_2_ fold change (log_2_FC) > |0.3| and p‐value <0.05. Stabilized proteins with log_2_FC > 0.3 and p‐value <0.05 are shown in red; destabilized proteins with log_2_FC < ‐0.3 and p‐value < 0.05 are shown in blue; Proteins with ‐0.3 < log_2_FC < 0.3 and p‐value < 0.05 and proteins with p‐value > 0.05 are shown in gray. **b)** Dose‐dependent stabilization of several RPs upon protein lysate treatment with **31**; *N* = 1 in triplicates; Data are expressed as log_2_FC (y‐axis) versus concentration (µM, x‐axis); Red inline is set at log_2_FC = 0.3; 60S ribosomal proteins (RPL) and 40S ribosomal protein (RPS). **c)** Assessment of protein synthesis levels using the SUnSET assay. Compound incubation was done at the trophozoite‐stage parasites (30–35 hpi). The mRNA translation levels were assessed by incorporation of puromycin, which is detected using an α‐puromycin antibody. α‐GAPDH served as a loading control. Assay was done in two independent experiments. An uncropped version of the nitrocellulose membrane is available at Figure S4A,B. d) Graph shows quantification of western blot for lanes 5–7. Data are presented as average normalized signal intensity ± s.d. from the two independent experiments. The quantified blots for all the lanes are shown in Figure S4C.

After lysate incubation with 100 µM of **31**, analysis of soluble protein fractions revealed that, out of 104 significantly stabilized proteins, 12 belonged to cytosolic ribosomal subunits, whereas no mitochondrial or apicoplast ribosomal subunits were detected (Figure 3a). In contrast, incubation with the control compound **34**, exhibiting 1000‐fold lower activity (*Pf*NF54 IC_50 _= 3,731 ± 536 nM), failed to stabilize any cytosolic ribosomal proteins (Table S11).

A concentration‐dependent iSPP profiling was performed to validate potential protein targets, where stabilization of a protein in response to increasing compound concentrations indicated direct binding. A concentration‐dependent stabilization of several cytosolic ribosomal protein subunits was observed when *P. falciparum* lysates were treated with five concentrations of **31** (0.01–100 µM). Across the four highest concentrations, seven ribosomal proteins (RP) consistently exhibited stabilization after lysate treatment with **31** (Figure 3b). Among them, the 60S ribosomal protein subunit L18a (RPL18a) (*UniProt ID: W7JPN9*) consistently showed the highest fold‐change at all concentrations (Figure 3b; Table S12). Furthermore, subunits of the eukaryotic initiation factor 3 (eIF3) complex were also stabilized. In addition, elF3 A along with six ribosomal subunits was already detectable even at just 0.1 µM of **31** (Table S12). These findings clearly indicate that **31** targets the cytosolic ribosomal complex.

To confirm the role of **31** as a translation inhibitor, the surface sensing of translation (SUnSET) assay was employed. This involves incubating the culture with a translation inhibitor, puromycin, a structural analogue of aminoacyl tRNA. Puromycin is incorporated in the nascent polypeptide chain and prevents further elongation. When puromcyin is used in sub‐lethal concentrations, incorporation of puromycin in neosynthesized protein correlates with the levels of mRNA translation (Figure 3c, lane2).^[^ ^27^ ^]^ Following a 5 h exposure, trophozoite‐stage parasites (30–35 hpi) incubated with **31** showed a concentration‐dependent reduction in translation (Figure 3c, lane 5–7). This trend was further confirmed by the quantified western blot signals (Figures 3d and S4C) that are consistent with the iSPP profiling results. This was observed at 3*x*, 50*x*, and 100*x* the IC_50_ of **31**, reaching levels comparable to those seen when parasites were treated with the protein synthesis inhibitor, cycloheximide (Figure 3c, lane 3). In contrast, exposure with **34**, acting as a negative control compound, did not reduce mRNA translation, suggesting that the ─OH group in the C‐ring is crucial for interaction within the ribosomal complex (Figure 3C, lane 4). Pyrimethamine, included as a negative control, also did not reduce mRNA translation (Figure S4A,B, lane 4). These results confirm that **31** interacts with the cytosolic ribosomal complex, disrupting protein synthesis and thus impairing parasite survival.

## Conclusions

The 2‐hydroxyphenyl benzamide are a novel potent chemical class that could serve as a good starting point for the development of a next‐generation antimalarial drug. The frontrunner **31** has a potent activity against the asexual blood stage (sub‐5 nM) *P. falciparum* and late‐stage gametocytes (sub‐500 nM), underlining its potential as a therapeutic and transmission‐blocking agent.

Compound **31** demonstrated low‐nanomolar killing potency against drug‐resistant malaria parasites, confirming no cross‐resistance with standard antimalarials. Mutations in *pfmdr1* led to the parasite's resistance to **31**. However, mutations identified in the *pfmdr1* gene do not decrease the sensitivity to other antimalarials, including the aryl amino alcohols, 4‐amnoquinolines and artesunate, positioning **31** as a valuable partner drug for combination therapies. This is an important attribute given that malaria treatment relies on drugs with different resistance profiles and molecular targets to reduce the onset of resistance in the field.

The MoA of **31** against *P. falciparum* was studied by target engagement and resistance selection. These studies supported the notion that **31** inhibits protein synthesis by engagement of multiple cytosolic ribosomal subunits. Indeed, a similar model was proposed for the antimalarial MEF where the drug was proposed to bind to cytosolic ribosomes and that the *P. falciparum* resistance to MEF is attributed to copy number increase in *pfmdr1*.^[^ ^19^, ^25^ ^]^ However, the lack of cross resistance between MEF and **31** indicates a specific resistance mechanism and subtle difference in how **31** interacts within the ribosomal complex. This mechanism also explains **31′**s activity against the asexual and sexual stages. *Plasmodium* parasites exhibit ribosomal heterogeneity, utilizing distinct ribosomal RNA (rRNA) types at various life stages.^[^ ^28^ ^]^ Specifically, the asexual A‐type rRNA predominates during the asexual blood stage, while the sporozoite S‐type rRNA is more abundant in sexual and mosquito stages.^[^ ^28^ ^]^ This ribosomal heterogeneity likely contributes to the observed differences in **31′**s potency between the asexual blood‐stage and stage V gametocytes. Further investigations of **31′**s efficacy against liver and mosquito stages remain to be done.

Finally, the high selective toxicity of **31** to the parasite over human cells implies that cytosolic ribosomes are an effective and safe target for antimalarial drug discovery.

## Supporting Information

The authors have cited additional references within the Supporting Information.^[29–59]^

Whole genome sequencing data was deposited on the European Nucleotide Archive, accession number PRJEB90281. The proteomics data from the iSPP assay was deposited at the ProteomeXchange with the accession number PXD065399.

## Conflict of Interests

The authors declare no conflict of interest.

## Supporting information



Supporting Information

## Data Availability

The data that support the findings of this study are available in the Supporting Information of this article.
