# Supplementary material for: A Novel Antimalarial Agent that Inhibits Protein Synthesis in Plasmodium falciparum
Source: Angew Chem Int Ed Engl. 2025 Oct 20;64(49):e202514085. doi: 10.1002/anie.202514085 (PMC12668308; doi:10.1002/anie.202514085)
Supplement: Supplementary file 1 — Supporting Information [file ANIE-64-e202514085-s001.docx]

**A Novel Antimalarial Agent that Inhibits Protein Synthesis in *Plasmodium falciparum***

Patricia Bravo^+^,^[a,b]^ Eleonora Diamanti^+^,^[c]^ Mostafa M. Hamed^‡^,^[c]^ Lorenzo Bizzarri^‡^,^[d]^ Natalie Wiedemar,^[a,b]^ Armin Passecker, ^[a,b]^ Nicolas M.B. Brancucci, ^[a,b]^ Anna Albisetti, ^[a,b]^ Christin Gumpp,^[a,b]^ Boris Illarionov,^[e]^ Markus Fischer, ^[e]^ Matthias Witschel,^[f]^ Tobias Schehl,^[f]^ Hannes Hahne,^[d]^ Pascal Mäser,^[a,b]^ Matthias Rottmann*,^[a,b]^ and Anna K. H. Hirsch* ^[c,g]^

[a] Dr. P. Bravo, Dr. N. Wiedemar, A. Passecker, Prof. Dr. N. Brancucci, Dr. A. Albisetti, C. Gumpp, Prof. Dr. P. Mäser, Dr. M. Rottmann
Medical Parasitology and Infection Biology
Swiss Tropical and Public Health Institute

Kreuzstrasse 2, 4123 Allschwil, Switzerland

Email: matthias.rottmann@swisstph.ch

[b] Dr. P. Bravo, Dr. N. Wiedemar, A. Passecker, Prof. Dr. N. Brancucci, Dr. A. Albisetti, C. Gumpp, Prof. Dr. P. Mäser, Dr. M. Rottmann

Universität Basel

Petersplatz 1, Basel 4003, Switzerland

[c] Prof. Dr. A. K. H. Hirsch, Dr. E. Diamanti, Dr. M. M. Hamed

Helmholtz Institute for Pharmaceutical Research Saarland (HIPS)─Helmholtz Centre for Infection Research (HZI),

PharmaScienceHub

Campus Building E8.1,

Saarbrücken 66123, Germany

[d] Dr. L Bizzarri, Dr. H. Hahne

OmicScouts GmbH, Lise-Meitner-Straße 30, D-85354 Freising, Germany

[e] Dr. Markus Fischer, Dr. Boris Illarionov

Hamburg School of Food Science, Institute of Food Chemistry, Grindelallee 117, 20146 Hamburg, Germany.

[f] Dr. M. Witschel, T. Schehl

BASF-SE Carl-Bosch-Strasse 38, 67056 Ludwigshafen, Germany

[g] Prof. Dr. A. K. H. Hirsch

Saarland University, Department of Pharmacy,

Helmholtz Institute for Pharmaceutical Research Saarland (HIPS)─Helmholtz Centre for Infection Research (HZI),

PharmaScienceHub

Campus Building E8.1,

Saarbrücken 66123, Germany.

Email: Anna.Hirsch@helmholtz-hips.de

[+] P.B and E.D contributed equally to this work.

[‡] M.M.H. and L.B. contributed equally to this work as second authors

[*] M.R. and A.H. contributed equally to this work

Table of Contents

[1 Supporting material 3](#_Toc209722947)

[1.1 Tables and Figures 3](#_Toc209722948)

[2 Chemistry 14](#_Toc209722949)

[2.1 Synthetic scheme 14](#_Toc209722950)

[2.2 General procedures 19](#_Toc209722951)

[2.3 Synthesis and Characterization of Compounds 1–61. 19](#_Toc209722952)

[2.4 Chemicals, Materials and Methods 35](#_Toc209722953)

[2.5 Abbreviations 35](#_Toc209722954)

[2.6 NMR spectra 35](#_Toc209722955)

[3 Biological Testing 94](#_Toc209722956)

[3.1 Materials 94](#_Toc209722957)

[3.2 Cytotoxicity assays 94](#_Toc209722958)

[3.3 Metabolic Stability in Liver S9 Fractions 94](#_Toc209722959)

[3.4 *In vitro* *P. falciparum* cell culture and [^3^H]-hypoxanthine incorporation assay 94](#_Toc209722960)

[3.5 Resistance selection 94](#_Toc209722961)

[3.6 Cloning via Limiting Dilution 95](#_Toc209722962)

[3.7 Illumina Whole Genome Sequencing Analysis and Sanger Sequencing 95](#_Toc209722963)

[3.8 Transfection construction 95](#_Toc209722964)

[3.9 Transfection and transgenic lines 96](#_Toc209722965)

[3.10 Surface sensing of translation (SUnSET) and Western Blotting 96](#_Toc209722966)

[3.11 Dose-response assay on gametocytes using the MitoTracker-based readout 96](#_Toc209722967)

[3.12 Statistical Analysis 97](#_Toc209722968)

[4 Proteomics 97](#_Toc209722969)

[4.1 *P. falciparum* lysate extraction 97](#_Toc209722970)

[4.2 iSPP profiling 97](#_Toc209722971)

[4.3 Sample preparation for LC-MS/MS analysis 97](#_Toc209722972)

[4.4 Liquid Chromatography and mass spectrometry data acquisition 98](#_Toc209722973)

[4.5 Peptide and protein identification and quantification 98](#_Toc209722974)

[4.6 iSPP profiling data analysis 98](#_Toc209722975)

[5 References 98](#_Toc209722976)

# Supporting material

## Tables and Figures

**Table S1.** *Pf*N54 IC_50_ values, and *in silico* physiochemical properties: calculated logarithim of the partition coefficient (*cLogP*), calculated logarithim of the distribution coefficient (*cLogD*), topological polar surface area (tPSA), hydrogen bond donors (HBD), and hydrogen bond acceptors (HBA) were calculated using StarDrop (www.optibrium.co.uk).

| **Code** | **Structure** | ***Pf*NF54**  IC_50_ ± s.d_._ [nM] | ***cLogP*** | ***cLogD*** | ***tPSA*** | ***HBD*** | ***HBA*** |
| --- | --- | --- | --- | --- | --- | --- | --- |
| **(1)** |  | 592±77.6 | 2.918 | 2.918 | 95.94 | 2 | 7 |
| **(2)** |  | >10000 | 3.065 | 3.065 | 84.94 | 1 | 7 |
| **(3)** |  | >10000 | 2.607 | 2.607 | 76.15 | 0 | 7 |
| **(4)** |  | >10000 | 2.56 | 2.56 | 87.15 | 1 | 7 |
| **(5)** |  | 4927±97.8 | 3.2 | 3.2 | 75.71 | 1 | 6 |
| **(6)** |  | 777±86 | 2.126 | 2.126 | 118.7 | 3 | 7 |
| **(7)** |  | 217±42.4 | 3.11 | 3.11 | 92.7 | 2 | 6 |
| **(8)** |  | 1910±254 | 3.72 | 3.72 | 104.7 | 3 | 7 |
| **(9)** |  | 574±66.53 | 3.292 | 3.292 | 95.94 | 2 | 7 |
| **(10)** |  | 1409±147 | 3.493 | 3.493 | 95.94 | 2 | 7 |
| **(11)** |  | 1026±116 | 3.449 | 3.449 | 95.94 | 2 | 7 |
| **(12)** |  | 197±28.2 | 2.497 | 2.497 | 105.2 | 2 | 8 |
| **(13)** |  | >10000 | 4.597 | 4.597 | 104.7 | 3 | 7 |
| **(14)** |  | 2783±389 | 3.935 | 3.935 | 104.7 | 3 | 7 |
| **(15)** |  | 539±92.1 | 2.918 | 2.918 | 95.94 | 2 | 7 |
| **(16)** |  | 361±83 | 3.005 | 3.005 | 95.94 | 2 | 7 |
| **(17)** |  | 2315±8.5 | 3.994 | 3.994 | 95.94 | 2 | 7 |
| **(18)** |  | 1495±81.2 | 4.054 | 4.054 | 95.94 | 2 | 7 |
| **(19)** |  | 1446±80 | 2.343 | 2.343 | 108.8 | 2 | 8 |
| **(20)** |  | 746±21.3 | 2.714 | 2.714 | 108.8 | 2 | 8 |
| **(21)** |  | >10000 | 3.056 | 3.056 | 108.8 | 2 | 8 |
| **(22)** |  | 216±32.3 | 3.353 | 3.353 | 108.8 | 2 | 8 |
| **(23)** |  | 69* | 3.056 | 3.056 | 108.8 | 2 | 8 |
| **(24)** |  | 17±3 | 4.78 | 4.78 | 71.45 | 2 | 5 |
| **(25)** |  | 34±4 | 4.427 | 4.427 | 71.45 | 2 | 5 |
| **(26)** |  | 158 ± 15.6 | 3.136 | 3.136 | 102.2 | 2 | 8 |
| **(27)** |  | 11±3.4 | 4.098 | 2.737 | 74.69 | 2 | 6 |
| **(28)** |  | 41±17.2 | 4.092 | 2.21 | 86.72 | 3 | 7 |
| **(29)** |  | 7.6±1.8 | 5.05 | 2.941 | 74.69 | 2 | 6 |
| **(30)** |  | 10±1.8 | 3.248 | 2.82 | 83.92 | 2 | 7 |
| **(31)** |  | 3.9±0.4 | 3.161 | 2.456 | 77.93 | 2 | 7 |
| **(32)** |  | 21.6±2.65 | 3.391 | 2.891 | 83.92 | 2 | 7 |
| **(33)** |  | 63±7.4 | 3.724 | 3.354 | 83.92 | 2 | 7 |
| **(34)** |  | 3731±536 | 3.747 | 3.363 | 63.69 | 1 | 6 |
| **(35)** |  | > 10000 | 2.737 | 2.698 | 76.58 | 1 | 7 |
| **(36)** |  | 808±172 | 3.505 | 2.919 | 66.85 | 2 | 6 |
| **(37)** |  | 21.0±1.13 | 3.575 | 3.262 | 85.53 | 1 | 8 |
| *n=2 experiments | | | | | | | |

**Table S2**. **31** effectiveness against drug-resistant *Plasmodium falciparum* strains

| Strain | Resistance-related locus | **31** IC_50_  [nM]^[a]^ | IC_50_ ratio relative to NF54 strain | IC_50_ fold shift relative to  Dd2 strain | Artesunate IC_50_  [nM]^[a]^ | IC_50_ ratio relative to NF54 strain | IC_50_ fold shift relative to  Dd2 strain |
| --- | --- | --- | --- | --- | --- | --- | --- |
| NF54 | Sensitive strain | 1.8±0.3 | 1.00 |  | 0.4±0.07 | 1.0 |  |
| K1 | *pfmdr1*^[b]^, *pfcrt*^[c]^, *pfdhfr*^[d]^, *pfdhps*^[e]^ | 1.3±0.2 | 0.7 |  | 0.2±0.01 | 0.3 |  |
| Dd2 | *pfmdr1*, *pfcrt*, *pfdhfr*, *pfdhps* | 9.3±0.4 | 5.1 | 1.0 | 0.3±0.2 | 0.5 | 1.0 |
| Dd2 DDD107458 | Dd2 + *pfeEF2*^[f]^ | 5.6±0.9 |  | 0.6 | 0.2±0.2 |  | 0.8 |
| Dd2 MMV390048 | Dd2 + *pfpI4k*^[g]^ | 4.8±1.5 |  | 0.5 | 0.3±0.1 |  | 0.9 |
| Dd2 GNF156 | Dd2 + *pfcarl*^[h]^ | 6.6±0.5 |  | 0.7 | 0.2±0.02 |  | 0.5 |
| Dd2 NITD609 | Dd2 + *pfatp4*^[i]^ | 6.2±2.5 |  | 0.7 | 0.2±0.01 |  | 0.6 |
| Dd2 ELQ300 | Dd2 + *pfcytb*^[j]^ | 4.4±0.1 |  | 0.5 | 0.2±0.01 |  | 0.7 |
| Dd2 DSM265 | Dd2 + *pfdhodh*^[k]^ | 2.8±0.5 |  | 0.3 | 0.2±0.01 |  | 0.6 |
| Dd2 Fosmidomycin | Dd2 + *pfdxr*^[l]^ | 2.4±0.6 |  | 0.3 | 0.2±0.01 |  | 0.8 |

^[a]^72-h [^3^H] hypoxanthhine incorporation assay. Data is expressed as mean±s.d.. The assay was performed in two independent biological experiments, each in biological duplicates; ^[b]^multidrug resistance protein-1; ^[c]^chloroquine resistance transporter; ^[d]^dihydrofolate reductase;  ^[e]^dihydropteroate synthase; ^[f]^translation elongation factor 2;^[g]^phosphatidylinositol 4-kinase;^[h]^cyclic amine resistance locus;^[i]^atpase 4;^[j]^cytochrome bc1complex;^[k]^dihydroorotate dehydrogenase;^[l]^1-deoxy-D-xylulose-5-phosphate reductoisomerase

**Table S3**. Frequency of recrudescence in **31**-pressured *Plasmodium falciparum* lines

| Parasite | Flask | Selection pressure | | | Initial inoculum | Day positive^[a]^ |
| --- | --- | --- | --- | --- | --- | --- |
|  |  |  | Day | **31** [nM] |  |  |
| Dd2b2 | 1 | 3x IC_50_  5x IC_50_  7x IC_50_ | 1-5  7-11  Until recrudescence | 58.4  97.3  136.2 | 2x10^9^ | 19 |
| Dd2b2 | 2 | 3x IC_50_  5x IC_50_  7x IC_50_ | 1-5  7-11  Until recrudescence | 58.4  97.3  136.2 | 2x10^9^ | 22 |
| Dd2b2 | 3 | 3x IC_50_  5x IC_50_  7x IC_50_ | 1-5  7-11  Until recrudescence | 58.4  97.3  136.2 | 2x10^9^ | 19 |

^[a]^ first ring was observed in the culture

**Table S4. 31** resistance to Dd2b2 parental and cloned resistance line

| Parasite | Flask | **31**  mean IC_50_ value ± s.d.  [nM] | p-value^[a]^ | Clones | IC_50_ fold change relative to Dd2b2^WT^ | Artesunate  mean IC_50_ value ± s.d.  [nM] | IC_50_ fold change relative to Dd2b2^WT^ | p-value^[a]^ | Number of assays^[b]^ |
| --- | --- | --- | --- | --- | --- | --- | --- | --- | --- |
| Dd2b2^WT^ | - | 13.3±1.7 | - | - | 1.0 | 1.7±0.5 | 1.0 | -- | 3 |
| Dd2b2**^31^**^-R1.1^ | 1 | 35.8±11.2 | 0.04 (ns) | Yes | 2.7 | 1.6±0.3 | 1.0 | 0.98 (ns) | 2 |
| Dd2b2**^31^**^-R1.2^ | 1 | 158.9±32.9 | ***<0.001 | Yes | 11.9 | 2.2±0.6 | 1.3 | 0.41 (ns) | 2 |
| Dd2b2**^31^**^-R2.1^ | 2 | 225.1±35.3 | ***<0.001 | Yes | 16.9 | 2.6±1.5 | 1.6 | 0.52 (ns) | 3 |
| Dd2b2**^31^**^-R2.2^ | 2 | 154.2±26.3 | ***<0.001 | Yes | 11.6 | 3.7±1.2 | 2.2 | 0.06 (ns) | 2 |
| Dd2b2**^31^**^-R3.1^ | 3 | 192.5±35.3 | ***<0.001 | Yes | 14.5 | 1.2±0.6 | 0.7 | 0.48 (ns) | 3 |
| Dd2b2**^31^**^-R3.2^ | 3 | 264.3±40.5 | ***<0.001 | Yes | 19.8 | 2.4±0.3 | 1.4 | 0.30 (ns) | 3 |

[a] Student`s *t*-test was performed when comparing parental clone and the resistant clones. ***P-value < 0.001, **P-value < 0.002, and *P-value < 0.033; ns = not significant; [b] The assay was performed in three independent biological experiments

**Table S5**. Illumina whole genome sequencing and variant filtration

| **Parasite clones** | Dd2b2 | Dd2b2 | Dd2b2 | Dd2b2 |
| --- | --- | --- | --- | --- |
| Name | Dd2b2^WT^ | Dd2b2**^31^**^-R2.1^ | Dd2b2**^31^**^-R3.1^ | Dd2b2**^31^**^-3.2^ |
| % reads mapped | 96% | 93% | 95% | 96% |
| **Variants identified (SNPs and INDEL)** |  |  |  |  |
| raw variants | 156`127 | | | |
| variants that pass GATK default quality filtering | 90`778 | | | |
| nonsynonymous variants | 25`593 | | | |
| variants absent in Dd2b2^WT^ | 2`220 | | | |
| variants with QUAL > 500^[a]^ | 511 | | | |
| variants with a genotype quality = 99 for Dd2b2^WT^ | 59 | | | |
| variants that pass IGV visual inspection | 3 | | | |
| genes mutated in all samples | PF3D7_0523000 (pfmdr1), PF3D7_0305500 (protein dopey homolog) | | | |

^[a]^ Expressed as the Phred-scale probability that a given reference or alternative allele is present in a given genomic location

**Table S6**. Variants that pass filtering criteria and IGV visual inspection

| **Gene Name** |  | ***Pf*3D7_0523000** | | ***Pf*3D7_0305500** |
| --- | --- | --- | --- | --- |
| Chromosome | | *Pf*3D7_05_v3 | *Pf*3D7_05_v3 | *Pf*3D7_03_v3 |
| Position | | 958433 | 961084 | 262939 |
| Gene Description | | multidrug resistance protein 1 | multidrug resistance protein 1 | protein dopey homolog |
| Quality |  | 9540.11 | 886.75 | 6340.27 |
| Ref_Base |  | G | A | C |
| Alt_Base |  | A | T | CAAT |
| Type |  | SNP | SNP | INDEL |
| Effect |  | missense_variant | missense_variant | conservative_inframe_insertion |
| Codon Change | | c.544G>A | c.3195A>T | c.10345_10347dupAAT |
| Amino Acid Change | | p.Gly182Ser | p.Lys1065Asn | p.Asn3449dup |
| **Dd2b2^WT^** | Genotype^[a]^ | 0/0 | 0/0 | 0/0 |
|  | Total Reads | 146 | 90 | 67 |
|  | AlleleFreqAlt | 0% | 0% | 0% |
| **Dd2b2^31-R2.1^** | Genotype^[a]^ | 0/1 | 0/0 | 1/1 |
|  | Total Reads | 281 | 113 | 72 |
|  | AlleleFreqAlt | 63% | 0% | 96% |
| **Dd2b2^31-R3.1^** | Genotype^[a]^ | 0/1 | 0/1 | 0/1 |
|  | Total Reads | 179 | 200 | 66 |
|  | AlleleFreqAlt | 30% | 25% | 30% |
| **Dd2b2^31-R3.2^** | Genotype^[a]^ | 0/1 | 0/0 | 1/1 |
|  | Total Reads | 249 | 133 | 66 |
|  | AlleleFreqAlt | 66% | 0% | 100% |

^[a]^ Assigned genotype: 1/1 for > 90% Allele frequency alternative; 0/1 for 20-90% allele frequency alternative; 0/0 same as parent


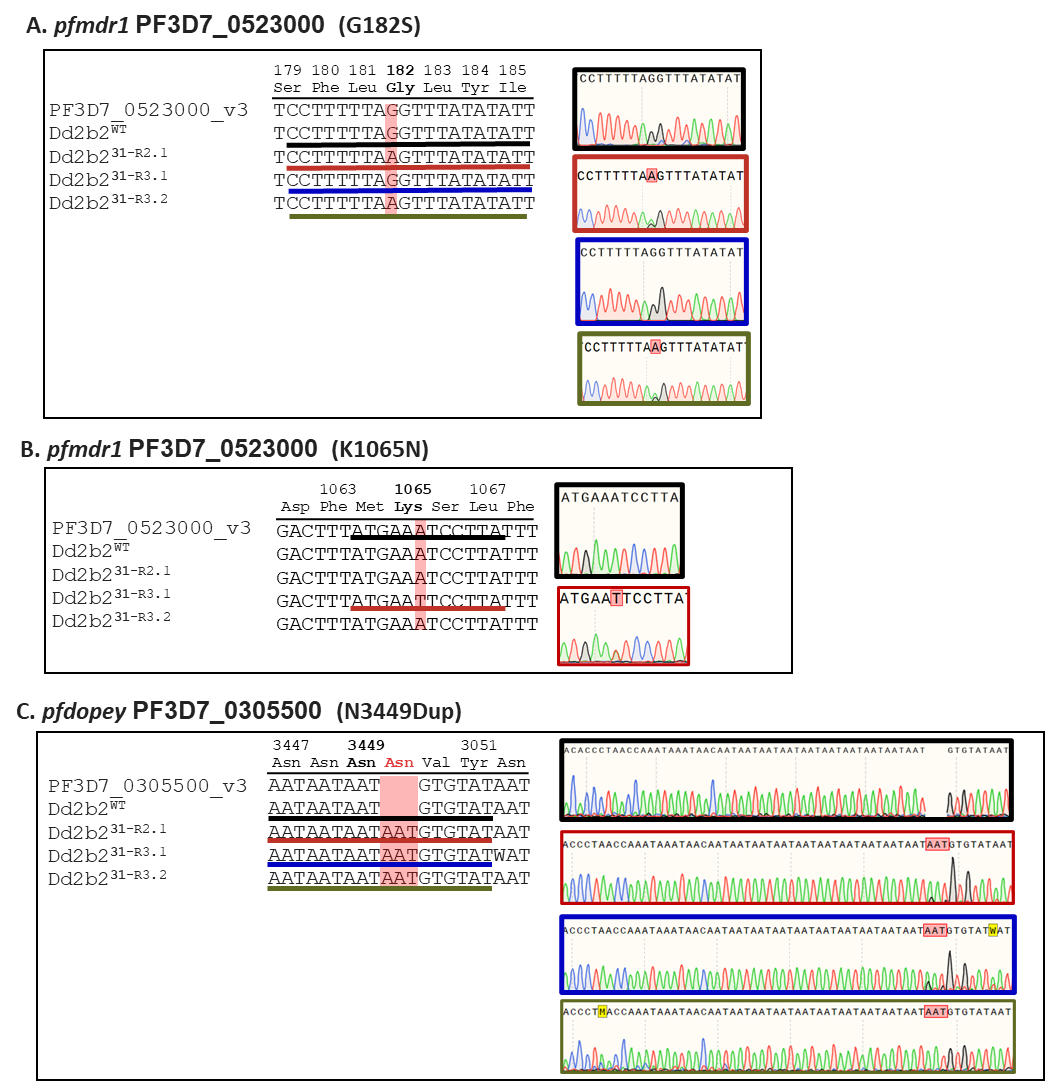


**Figure S1.** Sanger sequencing confirms variants identified from Illumina whole genome sequencing analysis of Dd2b2 **31-** selected clones except for the G182S mutation in *pfmdr1* for the clone Dd2b2**^31^**^-R3.1^


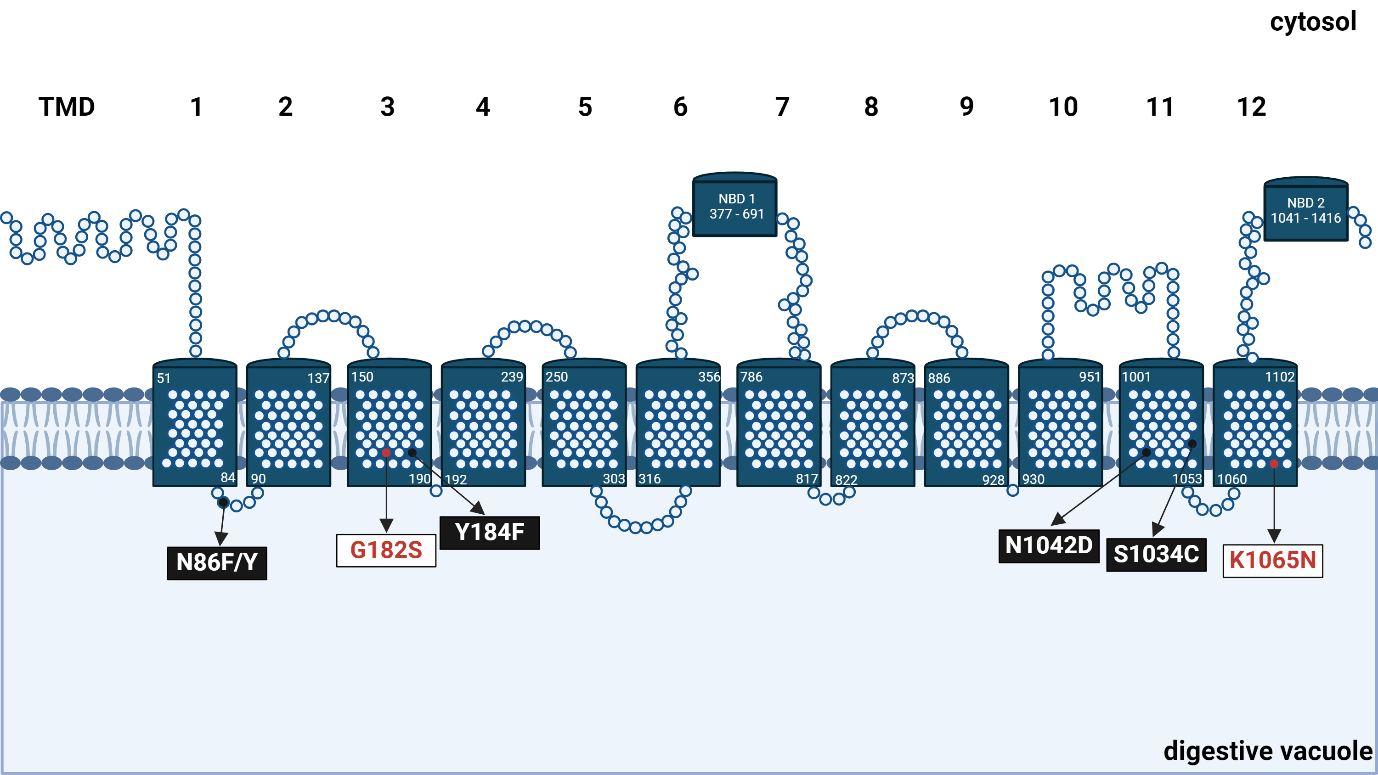


**Figure S2**. Localization of the polymorphisms identified in the PfMDR1 gene from **31-**selected clones (red) and polymorphisms associated with resistance to several antimalarial drugs (black). Figure is adapted from ^[29,30]^

**Table S7**. Several antimalarials do not have any cross-resistance to **31**-selected clones. All resistant strains bear the mutation *Pf*DOPEY^N3449dup^

| Compound | | Parent  (Dd2b2^WT)^  IC_50_  [nM]^[a]^ | | Dd2b2*^pfmdr1 G182S^*  IC_50_  [nM]^[a]^ | | IC_50_ fold shift relative to parental clone | p-value^[b]^ | Dd2b2 *^pfmdr1^* ^K1065N^  IC_50_  [nM]^[a]^ | | IC_50_ fold shift relative to parental clone | | p-value^[b]^ | |
| --- | --- | --- | --- | --- | --- | --- | --- | --- | --- | --- | --- | --- | --- |
| Mefloquine | | 1.1±0.2 | | 0.3±0.3 | 0.3 | 0.950 | | 0.90±0.2 | | 0.8 | | 0.990 | |
| Lumefantrine | | 1.6±0.8 | | 0.7±0.04 | 0.4 | 0.960 | | 1.23±0.6 | | 0.8 | | 0.990 | |
| Chloroquine | | 22.3±1.3 | | 17.7±8.0 | 0.8 | 0.660 | | 20.56±4.7 | | 0.9 | | 0.860 | |
| Artesunate | | 0.5±0.1 | | 0.6±0.2 | 1.3 | 0.990 | | 0.90±0.07 | | 2.5 | | 0.950 | |
| Amodiaquine | | 0.8±0.2 | | 0.8±0.01 | 1.1 | >0.99 | | 0.91±0.1 | | 1.2 | | 0.990 | |
| **27** | | 3.5±0.7 | | 36.9±4.1 | 10.6 | ***<0.001 | | 32.55±0.9 | | 9.4 | | ***<0.001 | |
| **30** | | 5.2±0.3 | | 68.8±19.6 | 19.1 | ***<0.001 | | 60.85±23 | | 11.8 | | ***<0.001 | |
| **31** | | 4.5±0.7 | | 159.4±8.7 | 35.7 | ***<0.001 | | 91.00±38.6 | | 20.2 | | ***<0.001 | |

^[a]^ 72-h [^3^H] hypoxanthhine incorporation assay; Data is expressed as mean±s.d. The assay was performed in three independent biological experiments, each in biological duplicates

^[b]^ Student`s *t*-test was performed when comparing parental and the resistant clones. ***P-value < 0.001, **P-value < 0.002, and *P-value < 0.033

**
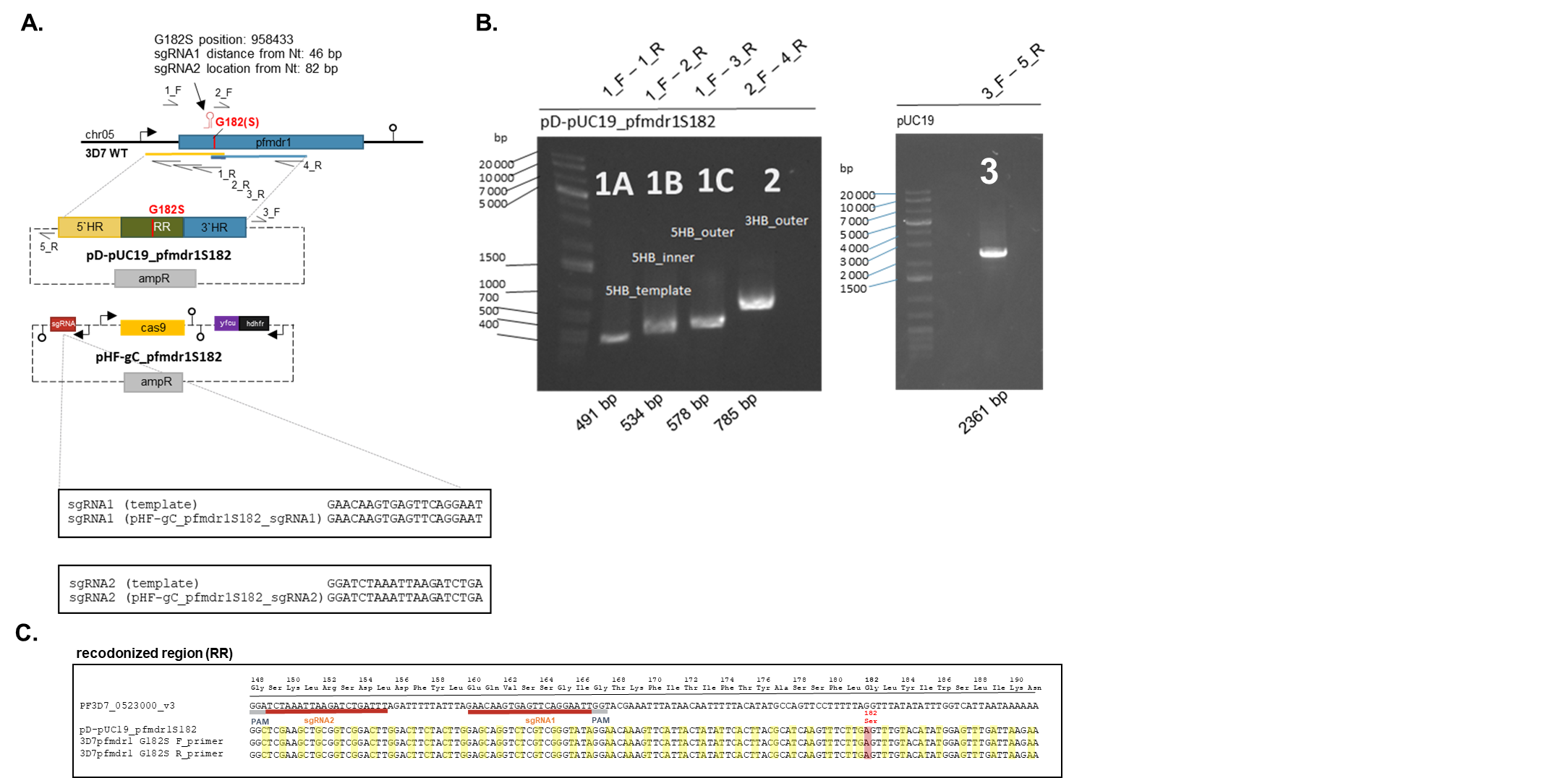
**

**Figure S3.** CRISPR/Cas9-based gene editing of the 3D7 *^pfmdr1^* ^G182S^ line using the two-plasmid CRISPR/Cas9 system.

**A**) Shows the donor (pD-pUC19_pfmdr1S182) and Cas9 (pHF-gC_pfmdr1S182) plasmids for the 3D7 ^pfmdr1G182S^ line. The residue 182 is in the genomic location 958433 of the *pfmdr1* gene (PF3D7_0523000). The sgRNA1 is 46 bp upstream of G182(S) whereas sgRNA2 is 82 bp upstream of G182(S). The pHF-gC_pfmdr1S182 plasmid contains the sgRNA construct which shows the sanger alignment of the sgRNA template and the sanger sequenced sgRNA in the pHF-gC_pfmdr1S182 plasmid. Both alignments are shown for sgRNA 1 and sgRNA 2.; **B**) Gels from the PCR amplification of the fragments used to design the pD-pUC19_pfmdr1S182 plasmid. The pD-pUC19_pfmdr1S182 plasmid is constructed consisting of a 5` and 3` homology regions (HR) which is on either side of the recodonized region (RR, 133 bp). The 5`HR and half of the RR are constructed from fragments 1A , 1B, and 1C. Fragment 1A (491 bp, amplified with primers 1_F and 1_R) was used as a template to amplify fragment 1B. Fragment 1B (534 bp, amplified with primers 1_F and 2_R) was used as a template to amplify fragment 1C. Fragment 1C was amplified using primers 1_F and 3_R). Fragment 1C (578 bp) was assembled with Fragment 2 which is half of the RR and the 3`HR (785 bp). The two fragments were assembled in a Gibson Assembly reaction with the PCR-amplified pUC19 vector (fragment 3, 2361 bp).; **C**) The alignment of the RR from the *Plasmodium falciparum 3D7* reference genome (PF3D7_0523000_v3), pD-pUC19_pfmdr1S182 plasmid, and the sanger sequenced gDNA of the 3D7 *^pfmdr1^* ^G182S^ line, which was sanger sequenced with a forward (F_primer) and reverse primer (R_primer). PAM motif is labelled and underlined in blue, sgRNAs are labelled and underlined in red, and G182S variant in 3D7 *^pfmdr1^* ^G182S^ line is highlighted in red. All primer sequences are listed in Table S11.

**Table S8.** sgRNAs and sequences with silent mutations

| sgRNA | Sequence | Sequence with silent mutations | Distance (#nt) away from | Doensch score | Editing success |
| --- | --- | --- | --- | --- | --- |
| sgRNA1 | GAACAAGTGAGTTCAGGAATTGG | gaGcaGgtCTCGtcGggTatAgg | 46 bp | 0.55 | 0/2 |
| sgRNA2 | GGATCTAAATTAAGATCTGATTT | gaGcaGgtCTCGtcGggTatAgg | 82 bp | 0.35 | 2/2 |

**Table S9.** Sensitivity to hydroxy derivatives and several antimalarials of parental and CRSIPR-Cas9 edited clone.

| Compound | 3D7^WT^  IC_50_ [nM]^[a]^ | 3D7^Cas9^ *^pfmdr1 G182S^*  IC_50_ [nM]^[a]^ | IC_50_ fold shift relative to 3D7^WT^  strain | Number of assays | p-value |
| --- | --- | --- | --- | --- | --- |
| Mefloquine | 1.4±0.5 | 1.9±0.2 | 1.3 | 3 | 0.53 |
| Lumefantrine | 1.6±0.8 | 1.33±0.6 | 0.8 | 3 | 0.990 |
| Chloroquine | 1.9±0.1 | 2.1±0.1 | 1.1 | 3 | 0.25 |
| Amodiaquine | 0.8±0.2 | 0.90±0.1 | 1.2 | 3 | 0.990 |
| Artesunate | 0.6±0.1 | 0.6±0.2 | 1.1 | 3 | 0.83 |
| **27** | 2.2±0.2 | 3.3±0.7 | 1.5 | 2 | 0.18 |
| **30** | 2.6±0.5 | 18.2±0.9 | 7.0 | 3 | ***<0.001 |
| **31** | 2.4±0.3 | 15.6±2.3 | 6.6 | 3 | *0.005 |

^[a]^ 72-h [^3^H] hypoxanthhine incorporation assay; Data is expressed as mean±s.d.. The assay was performed in three independent biological experiments, each in biological duplicates.

^[b]^ Student`s *t*-test was performed when comparing parental clone and the Cas9-edited clone. ***P-value < 0.001, **P-value < 0.002, and *P-value < 0.033

**Table S10**. Primers used in this study

| Primer name | Oligonucleotide sequence (5` 🡪3`) | Assay/Purpose |
| --- | --- | --- |
| 1 | ATGGGTAAAGAGCAGAAAGAG | *pfmdr1* G182(S) genotyping, forward primer |
| 2 | CACAACCTGATTCTCCCACAAAT | *pfmdr1* G182(S) genotyping, reverse primer |
| 3 | CACAACCTGATTCTCCCACAAAT | *pfmdr1* K1065(N) genotyping, forward primer |
| 4 | CACTACCTGTTTCTCCAACGAT | *pfmdr1* K1065(N) genotyping, reverse primer |
| 5 | GAAATGTGTTCTGATCTAATAGATG | *pfdopey* N3449(dup) genotyping, forward primer |
| 6 | GCTTACGTATGAAGATAAATCCTC | *pfdopey* N3449(dup) genotyping, reverse primer |
| 1_F | CGTTGGCCGATTCATTAATGATGGGTAAAGAGCAGAAAGAG | 5`HB *pfmdr1* G182S |
| 1_R | GAAGTCCAAGTCCGACCGCAGCTTCGAGCCAGGATTATTATCATGAAATTGTCCATCTTG | 5`HB *pfmdr1* G182S |
| 2_F | TTTCTTGAGTTTGTACATATGGAGTTTGATTAAGAACGCACGTTTGACTTTATGTATTAC | 3`HB *pfmdr1* G182S |
| 2_R | TAATGAACTTTGTTCCTATACCCGACGAGACCTGCTCCAAGTAGAAGTCCAAGTCCGACC | 5`HB *pfmdr1* G182S |
| 3_F | CTGGCGTAATAGCGAAGAGG | pUC19 amplification, forward primer |
| 3_R | CCATATGTACAAACTCAAGAAACTTGATGCGTAAGTGAATATAGTAATG | 5`HB *pfmdr1* G182S |
| 4_R | CCTCTTCGCTATTACGCCAGCGGTTGGATCATAAAGTCT | 3`HB *pfmdr1* G182S |
| 5_R | CATTAATGAATCGGCCAACG | pUC19 amplification, reverse primer |
| sgRNA1_F | TATTGAACAAGTGAGTTCAGGAAT | sgRNA1 for Cas9_plasmid_sgRNA1 |
| sgRNA1_R | AAACATTCCTGAACTCACTTGTTC | sgRNA1 for Cas9_plasmid_sgRNA1 |
| sgRNA2_F | TATTGGATCTAAATTAAGATCTGA | sgRNA2 for Cas9_plasmid_sgRNA2 |
| sgRNA2_R | AAACTCAGATCTTAATTTAGATCC | sgRNA2 for Cas9_plasmid_sgRNA2 |

**Table S11**. List of stabilized and destabilized proteins upon *P. falciparum* lysate treatment with 100 µM of the control compound **34.** The list of proteins shown are those proteins that pass our cut off (log2FC) > |0.3| and P-value < 0.05.

|  |  | **log2FC** |  |
| --- | --- | --- | --- |
| **Uniprot Protein.Ids** | **First.Protein.Description** | **100 μM c*ompound* 34** | |
| W7JRY1 | Antigen UB05 | 0.565050443 | |
| W7K941 | Pre-mRNA-splicing factor BUD31 | 0.519919078 | |
| W7KDE4 | Uncharacterized protein | 0.509712696 | |
| W7K9N9 | Phosphatidylinositol 4-kinase | 0.502393087 | |
| W7JSC9 | Uncharacterized protein | 0.456990878 | |
| W7KMX9 | Ribosome associated membrane protein RAMP4 | 0.445298831 | |
| W7KAG3 | RRM domain-containing protein | 0.444897493 | |
| W7K0B9 | 3-hydroxyacyl-[acyl-carrier-protein] dehydratase | 0.430964947 | |
| W7K5E1 | Aquaglyceroporin | 0.419904073 | |
| W7K576 | Small nuclear ribonucleoprotein Sm D1 | 0.401013374 | |
| W7JXH4 | Uncharacterized protein | 0.390377998 | |
| W7KAV2 | SPATR | 0.383458455 | |
| W7JQ66;W7K7Z8;W7KA08;W7KI56;W7KJ57 | Erythrocyte membrane protein 1 | 0.380375226 | |
| W7KMZ2 | Uncharacterized protein | 0.376235326 | |
| W7JPX7 | Ubiquitin-like protein nedd8-like | 0.365768433 | |
| W7K9U2 | Stevor | 0.364207586 | |
| W7KFM4 | Plasmodium RESA N-terminal domain-containing protein | 0.362263997 | |
| W7JRR7 | Pre-mRNA branch site protein p14 (Fragment) | 0.360066096 | |
| W7KFE6 | Uncharacterized protein | 0.353494644 | |
| W7KBT1 | Uncharacterized protein | 0.317988078 | |
| W7JVD0 | Dihydrolipoyl dehydrogenase | 0.311898867 | |
| W7K268 | Sugar phosphate phosphatase | -0.38131539 | |
| W7K0G9 | Uncharacterized protein | -0.38806057 | |
| W7JUC8 | PCI domain-containing protein | -0.388478756 | |
| W7KAK7 | Uncharacterized protein | -0.409343878 | |
| W7KAL3 | Uncharacterized protein | -0.485324542 | |
| W7KAW5 | Dual specificity protein phosphatase | -0.567771912 | |
| W7K1H5 | WD repeat-containing protein 82 | -0.651562373 | |
| W7JM37 | Protein RFT1 homolog | -0.6689442 | |
| W7K6H8 | DNA repair and recombination protein RAD54 | -0.756439845 | |
| W7JZL0 | Uncharacterized protein | -1.173489253 | |
| W7KNE8 | 2-C-methyl-D-erythritol 4-phosphate cytidylyltransferase | -1.33554697 | |

**Table S12**. List of proteins that overlap in the four highest doses of compound **31** incubated in *P.falciparum* lysate. All protein hits are significantly stabilized (P-value < 0.05) that pass the cutoff criteria (log2FC ≥ |3|).

|  |  | **log2FC** | | | | |
| --- | --- | --- | --- | --- | --- | --- |
| **Uniprot Protein.Ids** | **First.Protein.Description** | **100 μM** | **50 μM** | **10 μM** | **10^-2^ μM** | **10^-3^ μM** |
| W7KAD9 | 40S ribosomal protein S24 | 0.57 | 0.70 | 0.51 | 0.44 | 0.27 |
| W7JT49 | 40S ribosomal protein S4 | 0.62 | 0.68 | 0.53 | 0.39 | 0.19 |
| W7KA36 | 60S ribosomal protein L17 | 0.40 | 0.49 | 0.35 | 0.30 | 0.17 |
| W7JPN9 | 60S ribosomal protein L18a | 0.81 | 1.09 | 0.72 | 0.75 | 0.60 |
| W7K6D9 | 60S ribosomal protein L22 | 0.65 | 0.75 | 0.72 | 0.61 | 0.18 |
| W7KB54 | 60S ribosomal protein L4 | 0.50 | 0.63 | 0.49 | 0.41 | 0.21 |
| W7KAU4 | 60S ribosomal protein L7 | 0.74 | 0.94 | 0.75 | 0.69 | 0.33 |
| W7JWU4 | AAA family ATPase | 0.35 | 0.46 | 0.33 | 0.29 | 0.18 |
| W7JKR1 | ATP-dependent zinc metalloprotease FTSH | 0.46 | 0.64 | 0.49 | 0.46 | 0.20 |
| W7KFW7 | DNA primase large subunit | 0.41 | 0.51 | 0.33 | 0.50 | 0.39 |
| W7JVI6 | DnaJ protein | 0.40 | 0.51 | 0.38 | 0.30 | 0.12 |
| W7K3H0 | dolichyl-diphosphooligosaccharide--protein glycotransferase | 0.44 | 0.72 | 0.53 | 0.59 | 0.57 |
| W7KD62 | Eukaryotic translation initiation factor 3 subunit A | 0.31 | 0.45 | 0.35 | 0.30 | 0.15 |
| W7KAC4 | FACT complex subunit | 0.41 | 0.55 | 0.30 | 0.48 | 0.30 |
| W7JSF3;W7K4C4 | Phospholipid-transporting ATPase | 0.30 | 0.36 | 0.33 | 0.36 | 0.20 |
| W7K7J6 | PI3K/PI4K catalytic domain-containing protein | 0.41 | 0.44 | 0.30 | 0.42 | 0.17 |
| W7JWT0 | Ubiquitin-protein ligase | 0.59 | 0.87 | 0.52 | 0.50 | 0.25 |
| W7JXH4 | Uncharacterized protein | 0.70 | 0.61 | 1.06 | 0.54 | 0.61 |
| W7JXC3 | Uncharacterized protein | 0.40 | 0.70 | 0.50 | 0.36 | 0.02 |
| W7JQ86 | Uncharacterized protein | 0.37 | 0.58 | 0.49 | 0.40 | 0.28 |
| W7KIB1 | Uncharacterized protein | 0.88 | 1.03 | 1.08 | 1.16 | 0.39 |

**
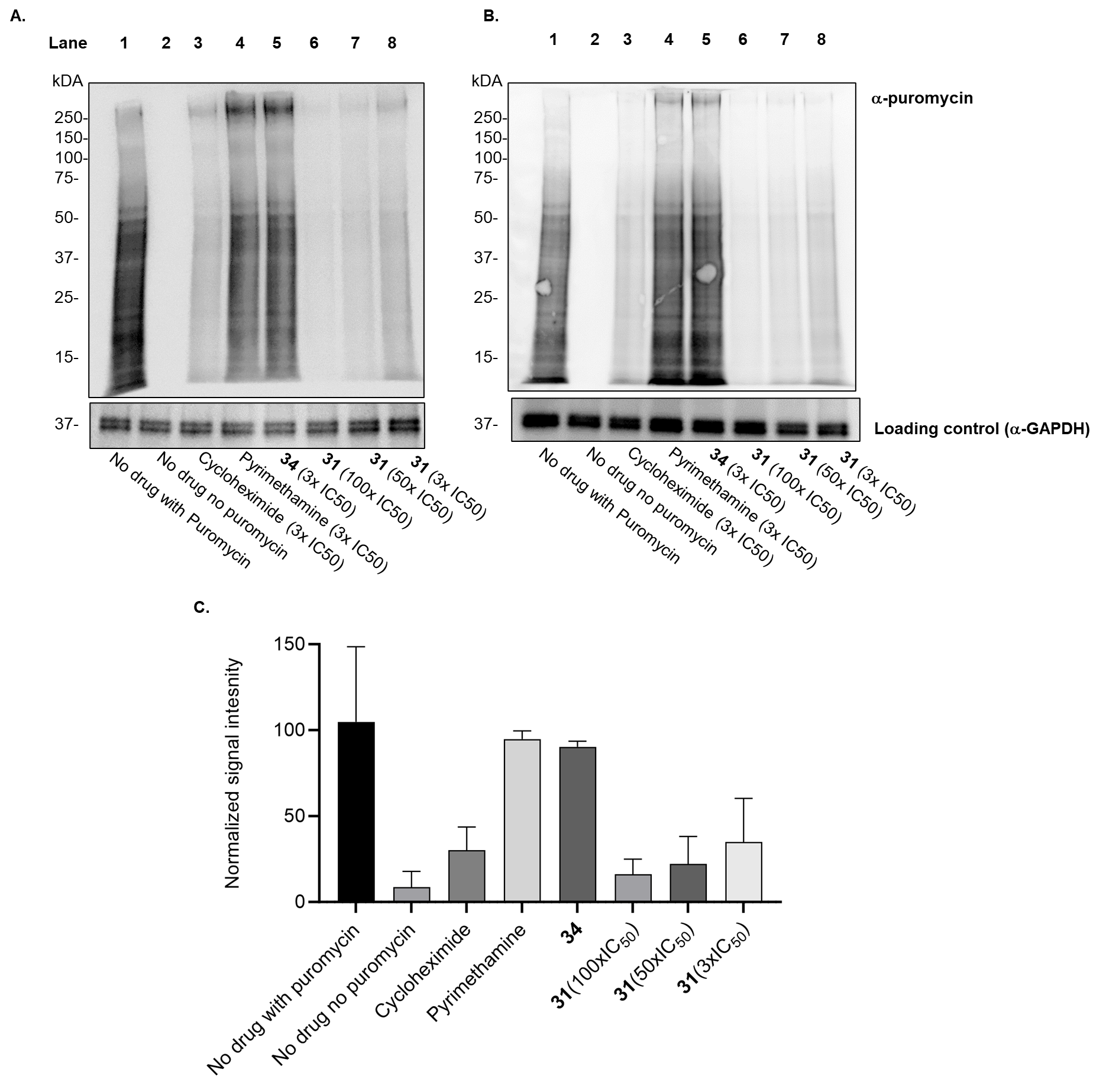
**

**Figure S4**. SUnSET assay confirms the role of **31** in interfering with protein translation in *Plasmodium falciparum.* Tightly synchronized NF54 parasites at 30-35 hpi (trophozoite stage) were treated with compounds for 4h. Incorporation of puromycin was detected using an α-puromycin antibody after 1h of incubation. The α-GAPDH served as a loading control. The assay was done in two independent biological replicates (4A, 4B). Replicate 4A is shown in main figure 3B (cropped version). Untreated control NF54 parasites were cultured in the absence of puromycin (lanes 2), confirming the specificity of α-puromycin antibody. Cycloheximide served as a positive control for protein translation inhibiton (lanes 3) whereas pyrimethamine (lane 4) and **34** (lane 5) served as negative controls. A dose-dependent decrease in protein translation was observed in lanes 6-8 for parasites treated with 100x, 50x and 3x IC_50_ of **31**. **C)** Representative quantification graph of the western blot signal intensity for the two independent biological experiments. Each signal was first normalized to the corresponding GAPDH loading control signal. The resulting normalized values were then averaged across replicates. Data are presented as average normalized values $\pm$ s.d.


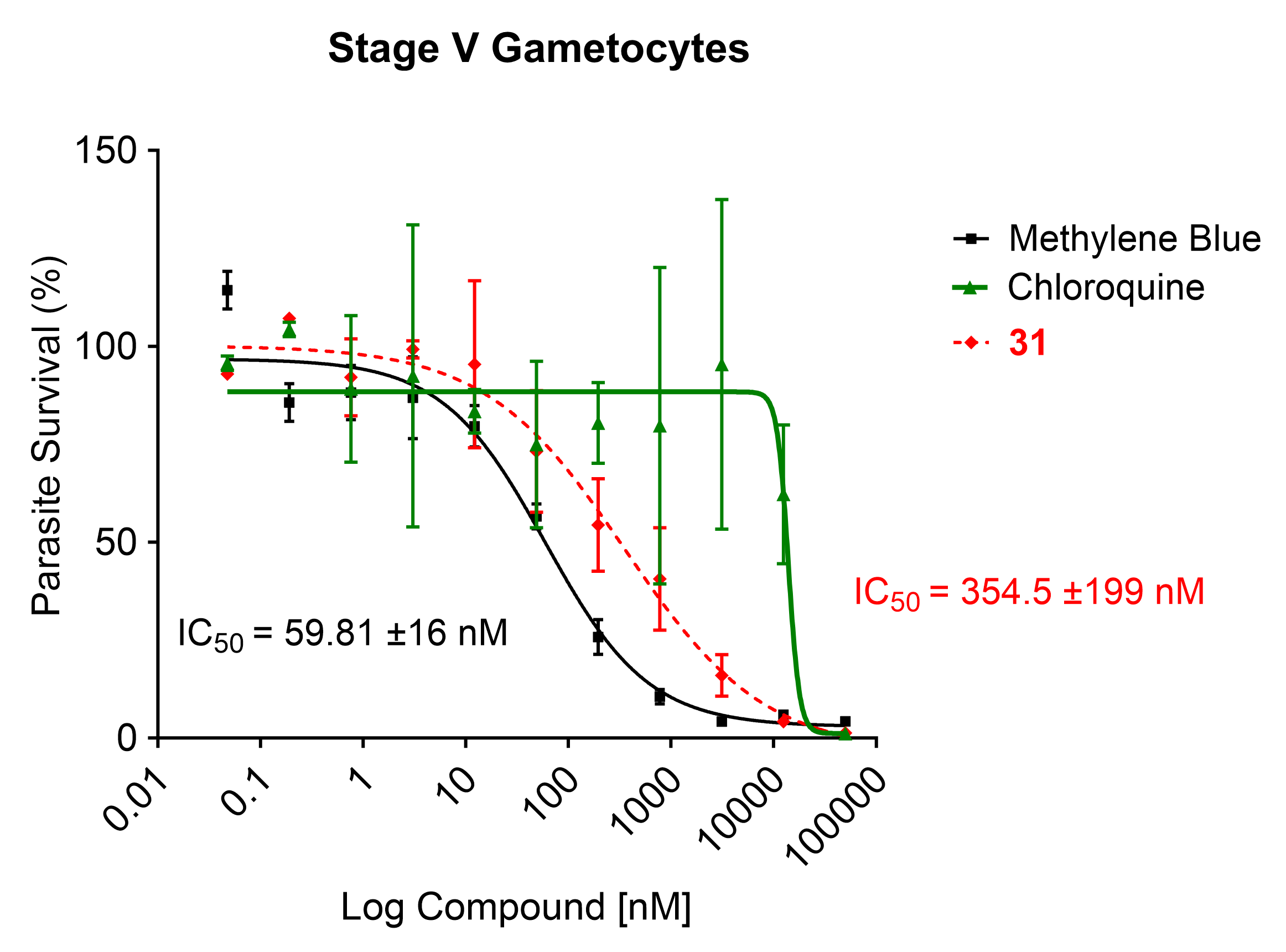


**Figure S5**. Compound **31** is active on stage V gametocytes. Dose-response curve is shown for tested against synchronous NF54/iGP1_RE9H^ulg8^ stage V gametocytes treated with compound **31** (red), control compounds Methylene Blue (black), and negative control chloroquine (green). Values on the y-axis represent normalized mean relative luminescence units (RLU) obtained from three biological replicates (mean ± s.e.m.). The mean IC_50_  ± S.E.M. value is also shown for methylene blue (black) and 31 (red). Assay was done in three independent assays (N=3).

# Chemistry

## Synthetic scheme

**Scheme S1**: Synthesis of compounds **1** and **5**.

**Scheme S2**: Synthesis of compounds **2**–**4**.

**Scheme S3**: Synthesis of **8**, **10**–**11**, **13**–**14**.

**Scheme S4:** Synthesis of **15**–**23**.

**Scheme S5:** Synthesis of **24** and **25**.

**Scheme S6**: Synthesis of **27**–**31**.

**Scheme S7**: Synthesis of **32**–**33**.

**Scheme S8:** Synthesis of **34** and **35**.

**Scheme S9:** Synthesis of **36**.

**Scheme S10:** Synthesis of **37**.

## General procedures

General procedure 1 (GP1): Sulfonamide formation *via* peptide coupling

Aromatic carboxylic acid (0.100 g, 1.0 equiv.) was dissolved in dry DMF (0.1 M), and TEA (2 equiv.) was added. After 10 minutes, HOBt (1.5 equiv.), EDC (1.5 equiv.) and were added, followed by the respective amine (1 equiv.). The reaction mixture was stirred at room temperature overnight. Upon completion of the reaction, the solution was poured onto water and the crude product extracted with EtOAc, washed with water and brine solution, and dried with Na_2_SO_4_. After removal of the solvent under reduced pressure, the crude product was purified by column chromatography (cyclohexane: EtOAc = 2:1) to yield the pure sulfonamide anilide products.

General procedure 2 (GP2): Sulfonamide formation *via* acyl chloride

To a stirred solution of 4-methoxy-phenoxybenzamide chloride (1.3 equiv) in dry DCM (0.2 M), Na_2_CO_3_ (1.5 equiv) and the corresponding amine (1.0 equiv) are added. The reaction mixture is left stirring at RT on.

General procedure 3 (GP3): De-methoxylation

Boron tribromide (1.0 M in DCM, 5.0 equiv) is added at -60°C to a stirring solution of the differently substituted methoxy benzamide (1.0 equiv) in DCM (0.1 M). The reaction mixture is left to stirring at RT on. The resulting solution was quenched with H_2_O and extracted with DCM (3 x 10 mL). The combined organic layers were dried over Na_2_SO_4_, filtered, concentrated in vacuo and purified by flash chromatography.

General procedure 4 (GP4): Reductive amination

To a stirred solution of the aldehyde (1.0 equiv), in DCE (0.2 M) the desired amine (1.3 equiv) and STAB (1.6 equiv) were added. The reaction mixture is left stirring at RT on. Upon completion of the reaction, H_2_O was added, and the aqueous phase was extracted with DCM (3x10 mL). The combined organic layers were dried over Na_2_SO_4_, filtered, concentrated in vacuo and purified by flash chromatography to afford the desired molecule.

## Synthesis and Characterization of Compounds 1–61.

***N*-(2-Hydroxy-5-(pyrrolidin-1-ylsulfonyl)phenyl)-3-phenoxybenzamide (1)**

Following GP1, 3-phenoxybenzoic acid (0.100 g, 1.0 equiv, 0.46 mmol), TEA (0.093 g, 0.92 mmol, 2.0 equiv), HOBt (0.093 g, 0.69 mmol, 1.5 equiv.), EDC (0.132 g, 0.69 mmol, 1.5 equiv.) and 2-amino-4-(pyrrolidin-1-ylsulfonyl)phenol 1a (0.111 g, 0.46 mmol, 1.0 equiv) reacted at RT on to afford after FCC **1** (Cyclohexane/EtOAc 2:1) as white solid (0.072 g, 0.164 mmol, 20%). ^1^H NMR (500 MHz, DMSO-*d_6_*) *δ* 10.94 (s, 1H), 9.66 (s, 1H), 8.15 (d, *J* = 1.8 Hz, 1H), 7.76 (d, *J* = 7.7 Hz, 1H), 7.60 – 7.52 (m, 2H), 7.48 (dd, *J* = 8.5, 2.2 Hz, 1H), 7.44 (t, *J* = 7.9 Hz, 2H), 7.26 (dd, *J* = 8.1, 2.3 Hz, 1H), 7.19 (t, *J* = 7.4 Hz, 1H), 7.09 (dd, *J* = 8.4, 2.5 Hz, 3H), 3.11 (m, 4H), 1.72 – 1.57 (m, 4H). ^13^C NMR (126 MHz, DMSO-*d_6_*) *δ* 164.6, 156.9, 156.2, 153.5, 136.0, 130.3, 130.2, 125.8, 125.7, 125.5, 123.9, 123.6, 122.4, 121.9, 119.0, 117.4, 115.7, 47.7, 24.6. HRMS (ESI) *m/z* calcd for C_23_H_21_N_2_O_5_S [*M*–H]^-^: 437.1177, found: 437.1179.

***N*-(2-Methoxy-5-(pyrrolidin-1-ylsulfonyl)phenyl)-3-phenoxybenzamide (2)**

Following GP2, **2b** (0.050 g, 0.12 mmol), pyrrolidine (0.006 mg, 0.09 mmol) and Na_2_CO_3_ (0.014 g, 0.13 mmol) in DCM (1.0 mL) afforded after purification by column chromatography (Hexane/EtOAc 7:3) the product **2** as colourless powder (0.035g, 87 %). ^1^H NMR (500 MHz, DMSO-*d_6_*) *δ* 9.73 (s, 1H), 8.20 (d, *J* = 2.1 Hz, 1H), 7.75 (d, *J* = 7.8 Hz, 1H), 7.63 (dd, *J* = 8.6, 2.3 Hz, 1H), 7.55 (t, *J* = 7.9 Hz, 2H), 7.44 (t, *J* = 7.9 Hz, 2H), 7.30 (d, *J* = 8.7 Hz, 1H), 7.26 (dd, *J* = 8.1, 2.4 Hz, 1H), 7.20 (t, *J* = 7.4 Hz, 1H), 7.08 (d, *J* = 7.9 Hz, 2H), 3.92 (s, 3H), 3.13 (t, *J* = 6.5 Hz, 4H), 1.66 (t, *J* = 6.6 Hz, 4H). ^13^C NMR (126 MHz, DMSO-*d_6_*) *δ* 164.6, 156.9, 156.1, 154.6, 135.9, 130.3, 130.2, 127.2, 127.0, 125.5, 123.9, 123.1, 122.4, 121.9, 119.0, 117.4, 111.6, 56.3, 47.8, 24.6. HRMS (ESI) *m/z* calcd for C_24_H_25_N_2_O_5_S [*M*+H]^+^: 453.1484, found: 453.1473.

***N*-(2-methoxy-5-(pyrrolidin-1-ylsulfonyl)phenyl)-*N*-methyl-3-phenoxybenzamide (3)**

To a solution of **2** (0.070 g, 0.154 mmol, 1.0 equiv) in dry THF (1 mL), NaH 60% (0.010 g, 0.154 mmol, 1.0 equiv) and CH_3_I (0.022 g, 0.154 mmol, 1.0 equiv) are added. The reaction is stirred at room temperature for 3 h and then washed with HCl 2M. The aqueous phase was extracted with EtOAc (3x10 mL), and the combined organic layers were dried over Na_2_SO_4_, filtered, concentrated in vacuo and purified by flash chromatography (Cyclohexane/EtOAc 1:1) to afford **3** as a white powder (0.045 g, 63%). ^1^H NMR (500 MHz, DMSO-*d_6_*) *δ* 7.69 (dd, *J* = 8.7, 2.3, 1H), 7.47 – 7.37 (m, 3H), 7.26 (t, *J* = 7.8, 1H), 7.19 (dd, *J* = 14.1, 8.0, 2H), 7.05 (d, *J* = 7.5, 1H), 6.96 (d, *J* = 8.0, 1H), 6.75 (d, *J*=7.6, 2H), 6.60 (s, 1H), 3.74 (s, 3H), 3.19 (s, 3H), 2.93 (s, 2H), 2.71 (s, 2H), 1.50 (s, 4H). ^13^C NMR (126 MHz, DMSO-*d_6_*) *δ* 169.1, 157.4, 156.1, 155.6, 137.7, 130.3, 129.8, 128.7, 128.6, 127.5, 124.0, 122.4, 119.7, 118.9, 116.1, 113.0, 56.2, 47.5, 24.5. HRMS (ESI) *m/z* calcd for C_25_H_27_N_2_O_5_S [*M*+H]^+^: 467.1641, found: 467.1630.

***N*-(2-Hydroxy-5-(pyrrolidin-1-ylsulfonyl)phenyl)-*N*-methyl-3-phenoxybenzamide (4)**

Following GP3, **3** (0.04 g, 0.086 mmol, 1.0 equiv) in DCM (1 mL) and BBr_3_ (5.0 equiv, 0.43 mL) afforded after flash chromatography (Cyclohexane/EtOAc 2:1) **4** as a white powder (0.029 g, 75%). ^1^H NMR (500 MHz, DMSO-*d_6_*) *δ* 11.11 (s, 1H), 7.54 (dd, *J* = 8.6, 2.1 Hz, 1H), 7.37 (t, *J* = 7.9 Hz, 2H), 7.31 (s, 1H), 7.25 (t, *J* = 7.9 Hz, 1H), 7.15 (t, *J* = 7.4 Hz, 1H), 7.07 (d, *J* = 7.3 Hz, 1H), 7.03 (d, *J* = 8.6 Hz, 1H), 6.93 (d, *J* = 7.9 Hz, 1H), 6.81 – 6.69 (m, 3H), 3.20 (s, 1H), 2.89 (m, 2H), 2.63 (m, 2H), 1.45 (m, 4H). ^13^C NMR (126 MHz, DMSO-*d_6_*) *δ* 169.1, 156.8, 155.9, 155.8, 138.0, 131.5, 130.2, 129.8, 129.3, 128.5, 125.9, 123.8, 122.5, 119.8, 118.5, 117.0, 116.8, 47.5, 35.7, 24.5. HRMS (ESI) *m/z* calcd for C_24_H_25_N_2_O_5_S [*M*+H]^+^: 453.1484, found: 453.1476.

**3-Phenoxy-*N*-(3-(pyrrolidin-1-ylsulfonyl)phenyl)benzamide (5)**

Following GP1, 3-phenoxybenzoic acid (0.214 g, 1.0 equiv, 1.0 mmol), TEA (0.203 g, 2.0 mmol, 2.0 equiv), HOBt (0.230 g, 1.5 mmol, 1.5 equiv.), EDC (0.289 g, 1.5 mmol, 1.5 equiv.), and 3-(pyrrolidin-1-ylsulfonyl)aniline **2a** (0.226 g, 1.0 mmol, 1.0 equiv) in DMF (2 mL, 0.5 M) afforded after FCC **5** as white solid (0.140 g, 32%). ^1^H NMR (500 MHz, DMSO-*d_6_*) *δ* 10.58 (s, 1H), 8.28 (s, 1H), 8.09 (d, *J* = 8.1 Hz, 1H), 7.78 (d, *J* = 7.6 Hz, 1H), 7.65 – 7.55 (m, 3H), 7.52 (d, *J* = 7.6 Hz, 1H), 7.44 (t, *J* = 7.1 Hz, 2H), 7.25 (d, *J* = 8.0 Hz, 1H), 7.19 (t, *J* = 7.2 Hz, 1H), 7.08 (d, *J* = 7.5 Hz, 2H), 3.16 (s, 4H), 1.66 (s, 4H). ^13^C NMR (126 MHz, DMSO-*d_6_*) *δ* 165.0, 156.8, 156.3, 139.8, 136.4, 136.2, 130.3, 130.2, 129.8, 124.2, 123.9, 122.7, 122.2, 122.0, 118.9, 118.7, 117.7, 47.9, 24.7. HRMS (ESI) *m/z* calcd for C_23_H_23_N_2_O_4_S [*M*+H]^+^: 423.1379, found: 423.1365.

***N*-(2-Hydroxy-5-sulfamoylphenyl)-3-phenoxybenzamide (6)**

The compound has been bought from Enamine (CAS = 2248944-62-7). ^1^H NMR (500 MHz, DMSO-*d_6_*) *δ* 9.67 (s, 1H), 8.15 (d, *J* = 2.3 Hz, 1H), 7.75 (d, *J* = 7.8 Hz, 1H), 7.59 – 7.57 (m, 1H), 7.55 (t, *J* = 8.0 Hz, 1H), 7.50 (dd, *J* = 8.5, 2.3 Hz, 1H), 7.46 – 7.40 (m, 2H), 7.25 (dd, *J* = 8.0, 2.2 Hz, 1H), 7.20 (d, *J* = 7.4 Hz, 1H), 7.18 (s, 2H), 7.08 (d, *J* = 7.7 Hz, 2H), 7.01 (d, *J* = 8.5 Hz, 1H). ^13^C NMR (126 MHz, DMSO-*d_6_*) *δ* 164.5, 156.9, 156.2, 152.9, 136.1, 134.4, 130.3, 130.2, 125.4, 123.9, 122.6, 122.4, 121.8, 119.0, 117.4, 115.4. HRMS (ESI) *m/z* calcd for C_19_H_17_N_2_O_5_S [*M*+H]^+^: 385.0858, found: 385.084.

***N*-(5-(Ethylsulfonyl)-2-hydroxyphenyl)-3-phenoxybenzamide (7)**

The compound has been bought from Enamine (CAS = [2249055-48-7](https://scifinder-n.cas.org/navigate/?appId=c76503db-a72b-4e35-9668-dc6394ef74f8&clearSearch=true&isFromAllResults=false&ordinal=0&previousInitiatingActionId=fbebfda9-e9e4-41d9-8fa4-cc45cdfb892f&resultType=substance&resultView=DETAIL&state=searchDetail.substance&suppressNavigation=true&uiContext=367&uiSubContext=678&uriForDetails=substance%2Fpt%2F2249055487&uriList=substance%2Fpt%2F2249055487)). ^1^H NMR (500 MHz, DMSO-*d_6_*) *δ* 11.06 (s, 1H), 9.69 (s, 1H), 8.20 (d, *J*=2.4 Hz, 1H), 7.75 (dd, *J* = 7.5, 1.5 Hz, 1H), 7.58 – 7.53 (m, 3H), 7.44 (t, *J* = 7.9 Hz, 2H), 7.25 (dd, J = 8.2, 2.5 Hz, 1H), 7.19 (t, *J* = 7.4 Hz, 1H), 7.10 (dd, *J* = 12.6, 8.2 Hz, 3H), 3.19 (q, *J* = 7.3 Hz, 2H), 1.10 (t, *J* = 7.3 Hz, 3H). ^13^C NMR (126 MHz, DMSO-*d_6_*) *δ* 164.6, 156.9, 156.2, 154.3, 135.9, 130.3, 130.2, 128.1, 126.0, 125.9, 124.2, 123.9, 122.4, 121.8, 118.9, 117.4, 115.8, 49.7, 7.4. HRMS (ESI) *m/z* calcd for C_21_H_20_NO_5_S [*M*+H]^+^: 398.1062, found: 398.1044.

***N*-(5-(*N*-Butylsulfamoyl)-2-hydroxyphenyl)-3-phenoxybenzamide (8)**

Following GP3, **8a** (0.075 g, 0.165 mmol) and BBr_3_ (0.82 mmol, 0.82 mL) in DCM (2 mL) afforded after prep HPLC (product at 65% MeCN + 0.1% FA) **8** as yellowish powder (0.053 g, 73%). ^1^H NMR (500 MHz, DMSO-*d_6_*) *δ* 10.81 (s, 1H), 9.65 (s, 1H), 8.14 (t, *J*=5.1, 1H), 7.75 (d, *J*=7.7, 1H), 7.60 – 7.52 (m, 2H), 7.48 – 7.40 (m, 3H), 7.35 (t, *J*=5.9, 1H), 7.25 (dd, *J*=8.1, 2.4, 1H), 7.19 (t, *J*=7.4, 1H), 7.08 (d, *J*=8.4, 2H), 7.04 (d, *J*=8.5, 1H), 2.70 (dd, *J*=13.3, 6.7, 2H), 1.39 – 1.29 (m, 2H), 1.27 – 1.18 (m, 2H), 0.79 (t, *J*=7.3, 3H). ^13^C NMR (126 MHz, DMSO-*d_6_*) *δ* 164.5, 156.9, 156.2, 153.1, 136.0, 130.4, 130.3, 130.2, 125.6, 124.7, 123.9, 123.1, 122.4, 121.8, 118.9, 117.4, 115.5, 42.1, 31.0, 19.2, 13.4. HRMS (ESI) *m/z* calcd for C_23_H_25_N_2_O_5_S [*M*+H]^+^: 441.1484, found: 441.1474.

***N*-(2-Hydroxy-5-(piperidin-1-ylsulfonyl)phenyl)-3-phenoxybenzamide (9)**

The compound has been bought from Enamine (CAS = 380165-04-8).^1^H NMR (500 MHz, DMSO-*d_6_*) *δ* 10.94 (s, 1H), 9.66 (s, 1H), 8.08 (d, *J*=1.6 Hz, 1H), 7.75 (d, *J*=7.8 Hz, 1H), 7.58 – 7.52 (m, 2H), 7.45 – 7.38 (m, 3H), 7.26 (dd, *J*=8.1, 2.3, 1H), 7.19 (t, *J*=7.4 Hz, 1H), 7.09 (dd, *J*=8.2, 5.7, 3H), 2.89 – 2.80 (m, 4H), 1.56 – 1.50 (m, 4H), 1.37 – 1.31 (m, 2H). ^13^C NMR (126 MHz, DMSO-*d_6_*) *δ* 164.6, 156.9, 156.2, 153.5, 135.9, 130.3, 130.2, 125.8, 125.7, 125.1, 123.9, 123.6, 122.4, 121.9, 118.9, 117.4, 115.7, 46.6, 24.6, 22.9. HRMS (ESI) *m/z* calcd for C_24_H_25_N_2_O_5_S [*M*+H]^+^: 453.1479, found: 453.1469.

***N*-(5-((3,3-*di*Methylpyrrolidin-1-yl)sulfonyl)-2-hydroxyphenyl)-3-phenoxybenzamide (10)**

Following GP3, **10a** (0.030 g, 0.062 mmol) and BBr_3_ (0.312 mmol, 0.312 mL) in DCM (2 mL) afforded after prep HPLC **10** as white powder (0.012 g, 41%). ^1^H NMR (500 MHz, Acetone-*d_6_*) *δ* 9.49 (s, 1H), 8.44 (d, *J* = 2.4 Hz, 1H), 7.81 (d, *J* = 7.7 Hz, 1H), 7.68 (t, *J* = 2.1 Hz, 1H), 7.60 – 7.52 (m, 2H), 7.43 (dd, *J* = 8.6, 7.3 Hz, 2H), 7.26 (dd, *J* = 8.2, 2.5 Hz, 1H), 7.19 (t, *J* = 7.4 Hz, 1H), 7.14 (d, *J* = 8.5 Hz, 1H), 7.12 – 7.05 (m, 2H), 3.31 (t, *J* = 7.1 Hz, 2H), 2.97 (s, 2H), 1.57 (t, *J* = 7.1 Hz, 2H), 0.90 (s, 6H). ^13^C NMR (126 MHz, Acetone-*d_6_*) *δ* 166.5, 158.9, 157.7, 153.2, 136.9, 131.4, 131.1, 129.1, 127.8, 127.7, 126.3, 125.0, 123.2, 123.2, 122.8, 122.7, 120.2, 118.6, 117.6, 61.1, 48.0, 39.8, 39.3, 26.4. HRMS (ESI) *m/z* calcd for C_25_H_27_N_2_O_5_S [*M*+H]^+^: 467.1635, found: 467.1628.

***N*-(2-Hydroxy-5-((4-methylpiperidin-1-yl)sulfonyl)phenyl)-3-phenoxybenzamide (11)**

Following GP3, **11a** (0.042 g, 0.087 mmol) and BBr_3_ (0.43 mmol, 0.43 mL) in DCM (2 mL) afforded after prep HPLC (product at 70% MeCN + 0.1% FA) **11** as white powder (0.023 g, 57%). ^1^H NMR (500 MHz, DMSO-*d_6_*) *δ* 11.18 (s, br, 1H), 9.66 (s, 1H), 8.09 (d, *J*=1.5, 1H), 7.74 (d, *J*=7.7, 1H), 7.55 (t, *J*=7.9, 2H), 7.44 (t, *J*=7.8, 2H), 7.39 (dd, *J*=8.5, 2.2, 1H), 7.25 (dd, *J*=8.2, 2.4, 1H), 7.19 (t, *J*=7.4, 1H), 7.07 (dd, *J*=10.5, 8.6, 3H), 3.55 (d, *J*=11.5, 2H), 2.18 (t, *J*=11.0, 2H), 1.64 (d, *J*=11.1, 2H), 1.35 – 1.19 (m, 1H), 1.13 (qd, *J*=12.4, 3.9, 2H), 0.85 (d, *J*=6.4, 3H). ^13^C NMR (126 MHz, DMSO-*d_6_*) *δ* 164.5, 156.9, 156.2, 154.0, 136.0, 130.3, 130.2, 126.0, 125.7, 124.5, 123.9, 123.3, 122.3, 121.9, 119.0, 117.4, 115.6, 46.1, 32.8, 29.3, 21.3. HRMS (ESI) *m/z* calcd for C_25_H_27_N_2_O_5_S [*M*+H]^+^: 467.1641, found: 467.16315.

***N*-(2-Hydroxy-5-(morpholinosulfonyl)phenyl)-3-phenoxybenzamide (12)**

The compound has been bought from Enamine (CAS =2249037-54-3). ^1^H NMR (500 MHz, DMSO-*d_6_*) *δ* 9.69 (s, 1H), 8.09 (d, *J*=1.9, 1H), 7.75 (d, *J*=7.8, 1H), 7.56 (dd, *J*=10.2, 5.3, 2H), 7.42 (dt, *J*=7.8, 5.2, 3H) 7.26 (dd, *J*=8.1, 2.2, 1H), 7.19 (t, *J*=7.4, 1H), 7.09 (dd, *J*=11.6, 8.2, 3H), 3.68 – 3.59 (m, 4H), 2.90 – 2.77 (m, 4H). ^13^C NMR (126 MHz, DMSO-*d_6_*) *δ* 164.7, 157.0, 156.3, 154.4, 136.1, 130.4, 130.3, 126.1, 124.0, 123.9, 123.7, 122.4, 122.1, 119.0, 117.4, 115.8, 65.3, 45.9. HRMS (ESI) *m/z* calcd for C_23_H_21_N_2_O_6_S [*M*-H]^-^: 453.1126, found: 453.1134.

***N*-(5-(*N*-(Cyclohexylmethyl)sulfamoyl)-2-hydroxyphenyl)-3-phenoxybenzamide (13)**

Following GP3, **13a** (0.090 g, 0.182 mmol) and BBr_3_ (0.91 mmol, 0.91 mL) in DCM (2 mL) afforded after prep HPLC **13** as white powder (0.028 g, 32%). ^1^H NMR (500 MHz, DMSO-*d_6_*) *δ* 10.78 (s, 1H), 9.65 (s, 1H), 8.13 (dd, *J*=10.4, 2.1, 1H), 7.75 (d, *J*=7.7, 1H), 7.56 (dd, *J*=13.2, 4.9, 2H), 7.48 – 7.40 (m, 3H), 7.38 (t, *J*=6.1, 1H), 7.25 (dd, *J*=8.1, 2.3, 1H), 7.19 (t, *J*=7.3, 1H), 7.08 (d, *J*=8.3, 2H), 7.04 (d, *J*=8.5, 1H), 2.57 – 2.51 (m, 2H), 1.78 – 1.48 (m, 5H), 1.41 – 1.25 (m, 1H), 1.18 – 0.98 (m, 3H), 0.90 – 0.69 (m, 2H). ^13^C NMR (126 MHz, DMSO-*d_6_*) *δ* 164.5, 156.9, 156.2, 153.0, 136.0, 130.6, 130.3, 130.2, 125.6, 124.7, 123.9, 123.1, 122.4, 121.8, 118.9, 117.4, 115.5, 48.7, 37.2, 30.2, 25.9, 25.3. HRMS (ESI) *m/z* calcd for C_26_H_27_N_2_O_5_S [*M*–H]^-^: 479.1646, found: 479.1651.

***N*-(5-(*N*-Benzylsulfamoyl)-2-hydroxyphenyl)-3-phenoxybenzamide (14)**

Following GP3, **14a** (0.042 g, 0.086 mmol) and BBr_3_ (0.43 mmol, 0.43 mL) in DCM (2 mL) afforded after prep HPLC **14** as white powder (0.018 g, 44%). ^1^H NMR (500 MHz, Acetone-*d_6_*) *δ* 10.08 (br, 1H), 9.44 (s, 1H), 8.44 (t, *J* = 2.7 Hz, 1H), 7.82 (d, *J* = 7.7 Hz, 1H), 7.68 (t, *J* = 2.1 Hz, 1H), 7.61 – 7.56 (m, 2H), 7.44 (t, *J* = 7.9 Hz, 2H), 7.33 – 7.24 (m, 5H), 7.21 (dd, *J*=14.5, 7.2, 2H), 7.10 (d, *J* = 8.3 Hz, 3H), 6.85 (t, *J* = 6.5 Hz, 1H), 4.11 (d, *J* = 6.7 Hz, 2H). ^13^C NMR (126 MHz, Acetone-*d_6_*) *δ* 166.5, 158.9, 157. 7, 152.6, 138.8, 136.9, 133.2, 131.4, 131.1, 129.3, 128.8, 128.2, 127.7, 125.8, 125.0, 123.2, 123.1, 122.4, 120.2, 118.6, 117.7, 47.9. HRMS (ESI) *m/z* calcd for C_26_H_21_N_2_O_5_S [*M*-H]^-^: 473.1177, found: 473.1179.

***N*-(2-Hydroxy-5-(pyrrolidin-1-ylsulfonyl)phenyl)-4-phenoxybenzamide (15)**

Following GP1, 4-phenoxybenzoic acid (0.100 g, 0.466 mmol, 1.0 equiv.), TEA (0.094 g, 0.93 mmol, 2.0 equiv), HOBt (0.094 g, 0.7 mmol, 1.5 equiv.), EDC (0.134 g, 0.7 mmol, 1.5 equiv.), and 3-(pyrrolidin-1-ylsulfonyl)aniline **1a** (0.113 g, 1.0 mmol, 1.0 equiv) in DMF (1 mL, 0.5 M) afforded after flash chromatography (Cyclohexane/EtOAc, product at 10% of EtOAc) **15** as white solid (0.080 g, 39%). ^1^H NMR (500 MHz, DMSO-*d_6_*) *δ* 9.55 (s, 1H), 8.24 (d, *J*=2.2, 1H), 8.01 (d, *J*=8.7, 2H), 7.50 – 7.41 (m, 3H), 7.24 (t, *J*=7.4, 1H), 7.15 – 7.04 (m, 5H), 3.18 – 3.06 (m, 4H), 1.70 – 1.59 (m, 4H). ^13^C NMR (126 MHz, DMSO-*d_6_*) *δ* 165.0, 160.5, 155.8, 153.9, 130.7, 130.3, 129.0, 126.7, 125.9, 125.6, 124.9, 123.4, 120.1, 117.9, 116.1, 48.2, 25.1. HRMS (ESI) *m/z* calcd for C_23_H_23_N_2_O_5_S [*M*+H]^+^: 439.1322, found: 439.1311.

**3-(3-Fluorophenoxy)-N-(2-hydroxy-5-(pyrrolidin-1-ylsulfonyl)phenyl)benzamide (16)**

Following GP1, 3-(4-fluorophenoxy)benzoic acid (0.100 g, 0.43 mmol, 1.0 equiv.), TEA (0.087 g, 0.86 mmol, 2.0 equiv), HOBt (0.086 g, 0.64 mmol, 1.5 equiv.), EDC (0.123 g, 0.64 mmol, 1.5 equiv.), and 3-(pyrrolidin-1-ylsulfonyl)aniline **1a** (0.104 g, 0.43 mmol, 1.0 equiv.) in DMF (1 mL, 0.5 M) afforded after flash chromatography (Cyclohexane/EtOAc, product at 10% of EtOAc) 16 as white solid (0.093 g, 22%). ^1^H NMR (500 MHz, DMSO-*d_6_*) *δ* 10.93 (s, 1H), 9.70 (s, 1H), 8.15 (d, *J*=1.9, 1H), 7.81 (d, *J*=7.8, 1H), 7.64 (s, 1H), 7.59 (t, *J*=7.9, 1H), 7.50 – 7.42 (m, 2H), 7.32 (dd, *J*=8.1, 2.2, 1H), 7.10 (dd, *J*=8.5, 5.2, 1H), 7.02 (td, *J*=8.5, 2.3, 1H), 6.96 (dt, *J*=10.4, 2.2, 1H), 6.90 (dd, *J*=8.2, 2.0, 1H), 3.13 – 3.09 (m, 4H), 1.68 – 1.63 (m, 4H). ^13^C NMR (126 MHz, DMSO) *δ* 164.5, 162.91 (d, *J*=244.9 Hz), 157.9, 157.8 (d, *J*=11.0 Hz), 156.0, 153.7, 136.2, 131.5, 131.4 (d, *J*=9.9), 130.5, 125.8, 125.7, 125.6, 123.8, 123.3, 122.5, 118.1, 115.8, 114.5 (d, *J* = 3.0 Hz), 110.5 (d, *J*= 21.0 Hz), 106.27 (d, *J*= 24.4 Hz), 47.8, 24.6. HRMS (ESI) *m/z* calcd for C_23_H_22_FN_2_O_5_S [*M*+H]^+^: 457.1228, found: 457.1221.

**3-(4-Chloro-3-(trifluoromethyl)phenoxy)-*N*-(2-hydroxy-5-(pyrrolidin-1-ylsulfonyl)phenyl)benzamide (17)**

Following GP1, 3-(4-chloro-3-(trifluoromethyl)phenoxy)benzoic acid (0.100 g, 0.316 mmol, 1.0 equiv.), TEA (0.064 g, 0.63 mmol, 2.0 equiv), HOBt (0.064 g, 0.47 mmol, 1.5 equiv.), EDC (0.091 g, 0.47 mmol, 1.5 equiv.), and 3-(pyrrolidin-1-ylsulfonyl)aniline **1a** (0.077 g, 0.316 mmol, 1.0 equiv) in DMF (2 mL, 0.5 M) afforded after flash chromatography (Cyclohexane/EtOAc, product at 10% of EtOAc) **17** as white solid (0.064 g, 26%). ^1^H NMR (500 MHz, DMSO-*d_6_*) *δ* 10.93 (s, 1H), 9.71 (s, 1H), 8.14 (d, *J*=2.0 Hz, 1H), 7.84 (d, *J*=7.8, 1H), 7.75 (d, *J*=8.8, 1H), 7.70 (s, 1H), 7.61 (t, *J*=8.0 Hz, 1H), 7.53 (d, *J*=2.9 Hz, 1H), 7.49 (dd, *J*=8.5, 2.3, 1H), 7.41 – 7.33 (m, 2H), 7.10 (d, *J*=8.5 Hz, 1H), 3.11 (t, *J*=6.6 Hz, 4H), 1.73 – 1.59 (m, 4H). ^13^C NMR (126 MHz, DMSO-*d_6_*) *δ* 164.4, 155.6, 155.5, 153.6, 136.3, 133.5, 130.6, 128.0 (q, *J*=31.3), 125.8, 125.7, 125.6, 124.8, 123.9, 123.8, 123.7, 122.9, 118.4, 117.9 (q, *J*=5.4), 115.8, 47.8, 24.6. HRMS (ESI) *m/z* calcd for C_24_H_21_ClF_3_N_2_O_5_S [*M*+H]^+^: 541.0806, found: 541.0800.

***N*-(2-hydroxy-5-(pyrrolidin-1-ylsulfonyl)phenyl)-3-(5-isopropyl-2-methylphenoxy)benzamide (18)**

Following GP1, 3-(5-isopropyl-2-methylphenoxy)benzoic acid (0.100 g, 0.37 mmol, 1.0 equiv.), TEA (0.075 g, 0.74 mmol, 2.0 equiv), HOBt (0.074 g, 0.55 mmol, 1.5 equiv.), EDC (0.106 g, 0.55 mmol, 1.5 equiv.), and 3-(pyrrolidin-1-ylsulfonyl)aniline **1a** (0.089 g, 0.37 mmol, 1.0 equiv) in DMF (2 mL, 0.5 M) afforded after flash chromatography (Cyclohexane/EtOAc, product at 10% of EtOAc) **18** as white solid (0.042 g, 23%). ^1^H NMR (500 MHz, DMSO-*d_6_*) *δ* 9.51 (s, 1H), 8.22 (d, *J*=2.2, 1H), 7.98 (d, *J*=8.8, 2H), 7.47 (dd, *J*=8.5, 2.3, 1H), 7.32 (t, *J*=7.2, 1H), 7.09 (d, *J*=8.5, 1H), 7.06 (d, *J*=8.1, 1H), 6.98 (d, *J*=8.8, 2H), 6.82 (s, 1H), 3.11 (t, *J*=6.6, 4H), 3.08 – 3.01 (m, 1H), 2.26 (s, 3H), 1.72 – 1.53 (m, 4H), 1.14 (s, 3H), 1.13 (s, 3H). ^13^C NMR (126 MHz, DMSO-*d_6_*) *δ* 164.7, 161.1, 153.1, 151.5, 137.0, 136.9, 129.9, 127.7, 127.1, 126.2, 126.3, 125.8, 125.1, 123.1, 121.2, 116.0, 115.7, 47.8, 26.4, 24.6, 23.0, 20.4. HRMS (ESI) *m/z* calcd for C_27_H_31_N_2_O_5_S [*M*+H]^+^: 495.1948, found: 495.1940.

***N*-(2-Hydroxy-5-(pyrrolidin-1-ylsulfonyl)phenyl)-3-(pyridin-2-yloxy)benzamide (19)**

Following GP1, 3-(pyridin-2-yloxy)benzoic acid (0.100 g, 0.46 mmol, 1.0 equiv.), TEA (0.094 g, 0.93 mmol, 2.0 equiv), HOBt (0.093 g, 0.69 mmol, 1.5 equiv.), EDC (0.132 g, 0.69 mmol, 1.5 equiv.), and 3-(pyrrolidin-1-ylsulfonyl)aniline **1a** (0.111 g, 0.46 mmol, 1.0 equiv) in DMF (2 mL, 0.5 M) afforded after flash chromatography (Cyclohexane/EtOAc, product at 10% of EtOAc) **17** as white solid (0.034 g, 17%). ^1^H NMR (500 MHz, DMSO-*d_6_*) *δ* 10.9 (br, 1H), 9.69 (s, 1H), 8.17 (dd, *J*=4.9 Hz, 1.5, 1H), 8.15 (d, *J*=2.1 Hz, 1H), 7.89 (td, *J*=8.4, 2.0 Hz, 1H), 7.84 (d, *J*=7.8 Hz, 1H), 7.73 (s, 1H), 7.58 (t, *J*=7.9, 1H), 7.49 (dd, *J*=8.5, 2.3, 1H), 7.38 (dd, *J*=8.0, 1.7 Hz, 1H), 7.17 (dd, *J*=6.9, 5.2, 1H), 7.11 (t, *J*=8.5 Hz, 2H), 3.22 – 3.01 (m, 4H), 1.74 – 1.59 (m, 4H). ^13^C NMR (126 MHz, DMSO-*d_6_*) *δ* 164.6, 162.8, 153.9, 153.7, 147.4, 140.4, 135.6, 129.9, 125.8, 125.8, 125.6, 124.8, 123.8, 123.7, 120.4, 119.4, 115.8, 111.7, 47.8, 24.6. HRMS (ESI) *m/z* calcd for C_22_H_22_N_3_O_5_S [*M*+H]^+^: 440.1275, found: 440.1266.

**3-((6-Chloropyridin-2-yl)oxy)-N-(2-hydroxy-5-(pyrrolidin-1-ylsulfonyl)phenyl)benzamide (20)**

Following GP1, 3-((6-chloropyridin-2-yl)oxy)benzoic acid (0.100 g, 0.401 mmol, 1.0 equiv.), TEA (0.081 g, 0.802 mmol, 2.0 equiv), HOBt (0.081 g, 0.60 mmol, 1.5 equiv.), EDC (0.115 g, 0.60 mmol, 1.5 equiv.), and 3-(pyrrolidin-1-ylsulfonyl)aniline **1a** (0.097 g, 0.401 mmol, 1.0 equiv) in DMF (2 mL, 0.5 M) afforded after flash chromatography (Cyclohexane/EtOAc, product at 10% of EtOAc) **20** as white solid (0.105 g, 28%). ^1^H NMR (500 MHz, DMSO-*d_6_*) *δ* = 10.94 (br, 1H), 9.72 (s, 1H), 8.15 (d, *J*=2.0 Hz, 1H), 7.95 (t, *J*=7.9 Hz, 1H), 7.88 (d, *J*=7.8 Hz, 1H), 7.77 (s, 1H), 7.62 (t, *J*=7.9 Hz, 1H), 7.49 (dd, *J*=8.5, 2.2, 1H), 7.44 (dd, *J*=8.1, 2.2, 1H), 7.29 (d, *J*=7.6, 1H), 7.10 (dd, *J*=8.3, 5.0 Hz, 2H), 3.12 (t, *J*=6.6 Hz, 4H), 1.66 (dd, *J*=8.0, 5.2, 4H). ^13^C NMR (126 MHz, DMSO-*d_6_*) *δ* 164.5, 162.3, 153.7, 153.3, 147.5, 143.3, 135.8, 130.1, 125.8, 125.7, 125.6, 124.7, 124.4, 123.8, 120.3, 119.1, 115.8, 110.4, 47.8, 24.6. HRMS (ESI) *m/z* calcd for C_22_H_21_ClN_3_O_5_S [*M*+H]^+^: 474.0885, found: 474.0877.

***N*-(2-Hydroxy-5-(pyrrolidin-1-ylsulfonyl)phenyl)-3-((6-(trifluoromethyl)pyridin-2-yl)oxy)benzamide (21)**

Following GP1, 3-((6-(trifluoromethyl)pyridin-2-yl)oxy)benzoic acid (0.100 g, 0.353 mmol, 1.0 equiv.), TEA (0.071 g, 0.706 mmol, 2.0 equiv), HOBt (0.071 g, 0.53 mmol, 1.5 equiv.), EDC (0.101 g, 0.53 mmol, 1.5 equiv.), and 3-(pyrrolidin-1-ylsulfonyl)aniline **1a** (0.085 g, 0.353 mmol, 1.0 equiv) in DMF (2 mL, 0.5 M) afforded after flash chromatography (Cyclohexane/EtOAc, product at 10% of EtOAc) **21** as white solid (0.029 g, 16%). ^1^H NMR (500 MHz, DMSO-*d_6_*) *δ* 9.70 (s, 1H), 8.20 – 8.13 (m, 2H), 7.89 (d, *J*=7.8, 1H), 7.81 (s, 1H), 7.68 (d, *J*=7.4, 1H), 7.63 (t, *J*=7.9, 1H), 7.48 (ddd, *J*=10.4, 8.4, 2.2, 2H), 7.41 (d, *J*=8.4, 1H), 7.09 (d, *J*=8.5, 1H), 3.18 – 3.03 (m, 4H), 1.74 – 1.54 (m, 4H). ^13^C NMR (126 MHz, DMSO-*d_6_*) *δ* 164.9, 163.2, 154.2, 153.5, 144.7 (q, *J*_CF_ = 34.5, *C*_quat_), 142.9, 136.3, 130.6, 126.3, 126.1, 126.0, 125.1, 124.9, 124.2, 120.7, 116.5 (d, *J*=3.1), 116.4, 116.2, 48.2, 25.1. HRMS (ESI) *m/z* calcd for C_23_H_21_F_3_N_3_O_5_S [*M*+H]^+^: 508.1149, found: 508.1147.

***N*-(2-hydroxy-5-(pyrrolidin-1-ylsulfonyl)phenyl)-3-((6-methyl-4-(trifluoromethyl)pyridin-2-yl)oxy)benzamide (22)**

Following GP1, 3-((6-methyl-4-(trifluoromethyl)pyridin-2-yl)oxy)benzoic acid (0.100 g, 0.336 mmol, 1.0 equiv.), TEA (0.068 g, 0.673 mmol, 2.0 equiv), HOBt (0.090 g, 0.67 mmol, 1.5 equiv.), EDC (0.128 g, 0.67 mmol, 1.5 equiv.), and 3-(pyrrolidin-1-ylsulfonyl)aniline **1a** (0.081 g, 0.336 mmol, 1.0 equiv) in DMF (2 mL, 0.5 M) afforded after flash chromatography (Cyclohexane/EtOAc, product at 10% of EtOAc) **22** as white solid (0.086 g, 25%). ^1^H NMR (500 MHz, DMSO-*d_6_*) *δ* 10.95 (s, 1H), 9.68 (s, 1H), 8.17 (d, *J*=2.1, 1H), 7.87 (d, *J*=7.8, 1H), 7.78 (s, 1H), 7.61 (t, *J*=7.9, 1H), 7.49 (dd, *J*=8.5, 2.2, 1H), 7.45 – 7.43 (m, 1H), 7.43 (s, 1H), 7.28 (s, 1H), 7.10 (d, *J*=8.5, 1H), 3.12 (t, *J*=6.6, 4H), 2.41 (s, 3H), 1.90 – 1.43 (m, 4H). ^13^C NMR (126 MHz, DMSO-*d_6_*) *δ* 164.5, 162.7, 159.3, 153.6, 153.4, 140.6 (q, *J*=33.1), 135.8, 130.1 (d, *J*=10.7), 125.8, 125.7, 125.6, 124.8, 124.2, 123.7, 120.3, 115.7, 113.93 (q, *J*=3.1), 104.89 (q, *J*=3.8), 47.8, 24.6, 23.7. HRMS (ESI) *m/z* calcd for C_24_H_23_F_3_N_3_O_5_S [*M*+H]^+^: 522.1305, found: 522.1298.

***N*-(2-Hydroxy-5-(pyrrolidin-1-ylsulfonyl)phenyl)-3-((4-(trifluoromethyl)pyridin-2-yl)oxy)benzamide (23)**

Following GP1, 3-((4-(trifluoromethyl)pyridin-2-yl)oxy)benzoic acid (0.100 g, 0.353 mmol, 1.0 equiv.), TEA (0.071 g, 0.706 mmol, 2.0 equiv), HOBt (0.071 g, 0.53 mmol, 1.5 equiv.), EDC (0.101 g, 0.53 mmol, 1.5 equiv.), and 3-(pyrrolidin-1-ylsulfonyl)aniline **1a** (0.085 g, 0.353 mmol, 1.0 equiv) in DMF (2 mL, 0.5 M) afforded after flash chromatography (Cyclohexane/EtOAc, product at 10% of EtOAc) **23** as white solid (0.011 g, 6%). ^1^H NMR (500 MHz, DMSO-*d_6_*) *δ* 9.70 (s, 1H), 8.42 (d, *J*=5.2, 1H), 8.16 (d, *J*=2.2, 1H), 7.88 (d, *J*=7.9, 1H), 7.81 – 7.78 (m, 1H), 7.62 (t, *J*=7.9, 1H), 7.56 (s, 1H), 7.53 (d, *J*=5.2, 1H), 7.47 (ddd, *J*=10.4, 8.3, 2.0, 2H), 7.07 (d, *J*=8.5, 1H), 3.11 (t, *J*=6.7, 5H), 1.73 – 1.59 (m, 4H). ^13^C NMR (126 MHz, DMSO-*d_6_*) *δ* 164.5, 163.5, 154.1, 153.2, 149.5, 135.9, 130.1, 125.9, 125.7, 125.1, 124.4, 123.7, 120.8, 115.8, 114.7, 108.2, 47.8, 24.7. ^19^F NMR (470 MHz, DMSO-*d_6_*) *δ* = -63.36. HRMS (ESI) *m/z* calcd for C_24_H_21_F_3_N_3_O_5_S [*M*+H]^+^: 508.1149, found: 508.1140.

***N*-(5-(*tert*-Butyl)-2-hydroxyphenyl)-3-((4-(trifluoromethyl)pyridin-2-yl)oxy)benzamide (24)**

Following GP1, 3-((4-(trifluoromethyl)pyridin-2-yl)oxy)benzoic acid (0.100 g, 0.35 mmol, 1.0 equiv.), TEA (0.071 g, 0.706 mmol, 2.0 equiv), HOBt (0.071 g, 0.52 mmol, 1.5 equiv.), EDC (0.81 g, 0.52 mmol, 1.5 equiv.), and 2-amino-4-(*tert*-butyl)phenol (0.058 g, 0.35 mmol, 1.0 equiv) in DMF (1 mL, 0.5 M) afforded after flash chromatography (Cyclohexane/EtOAc, product at 10% of EtOAc) **24** as white solid (0.075 g, 50% yield). ^1^H NMR (500 MHz, DMSO-*d_6_*) *δ* 9.66 (s, 1H), 9.39 (s, 1H), 8.43 (d, *J*=5.2, 1H), 7.88 (d, *J*=7.7, 1H), 7.80 (s, 1H), 7.61 (t, *J*=8.0, 1H), 7.58 (d, *J*=1.7, 1H), 7.56 (s, 1H), 7.53 (d, *J*=5.3, 1H), 7.47 – 7.41 (m, 1H), 7.08 (dd, *J*=8.4, 2.1, 1H), 6.84 (d, *J*=8.4, 1H), 1.25 (s, 9H). ^13^C NMR (126 MHz, DMSO-*d_6_*) *δ* 164.4, 163.5, 153.2, 149.4, 147.5, 141.3, 140.4 (q, *J*=33.3), 136.1, 130.0, 124.9, 124.8, 124.4, 122.8, 121.7, 120.7, 115.8, 114.6 (d, *J*=3.1), 108.1 (d, *J*=3.6), 33.8, 31.4. HRMS (ESI) *m/z* calcd for C_23_H_22_F_3_N_2_O_3_ [*M*+H]^+^: 431.1577, found: 431.1570.

***N*-(2-Hydroxy-5-isopropylphenyl)-3-((4-(trifluoromethyl)pyridin-2-yl)oxy)benzamide (25)**

Following GP1, 3-((4-(trifluoromethyl)pyridin-2-yl)oxy)benzoic acid **24a** (0.100 g, 0.35 mmol, 1.0 equiv.), TEA (0.071 g, 0.706 mmol, 2.0 equiv), HOBt (0.071 g, 0.52 mmol, 1.5 equiv.), EDC (0.81 g, 0.52 mmol, 1.5 equiv.), and 2-amino-4-isopropylphenol (0.053 g, 0.35 mmol, 1.0 equiv) in DMF (1 mL, 0.5 M) afforded after flash chromatography (Cyclohexane/EtOAc, product at 10% of EtOAc) **25** as white solid (0.017 g, 11% yield). ^1^H NMR (500 MHz, DMSO-*d_6_*) *δ* 9.61 (s, 1H), 9.41 (s, br, 1H), 7.88 (d, *J*=7.8, 1H), 7.79 (s, 1H), 7.60 (t, *J*=7.9, 1H), 7.56 (s, 1H), 7.53 (d, *J*=5.3, 1H), 7.48 – 7.42 (m, 2H), 6.92 (dd, *J*=8.3, 2.1, 1H), 6.83 (d, *J*=8.3, 1H), 2.80 (hept, *J*=6.9, 1H), 1.18 (s, 3H), 1.16 (s, 3H). ^13^C NMR (126 MHz, DMSO-*d_6_*) *δ* 164.3, 163.5, 153.2, 149.4, 147.8, 140.4 (q, *J*=33.5), 138.9, 136.1, 130.0, 125.2, 124.8, 124.3, 123.8, 122.5, 120.7, 115.9, 114.6 (d, *J*=3.0), 108.1 (q, *J*=3.7), 32.7, 24.1. HRMS (ESI) *m/z* calcd for C_22_H_20_F_3_N_2_O_3_ [*M*+H]^+^: 417.1421, found: 417.14124.

***N*-(2-Hydroxy-5-(4*H*-1,2,4-triazol-4-yl)phenyl)-3-((4-(trifluoromethyl)pyridin-2-yl)oxy)benzamide (26)**

The compound has been bought from Enamine (CAS =380165-61-7). ^1^H NMR (500 MHz, DMSO-*d_6_*) *δ* 10.25 (s, 1H), 9.75 (s, 1H), 8.97 (s, 2H), 8.43 (d, *J*=5.2, 1H), 7.92 (d, *J*=2.7, 1H), 7.90 (d, *J*=7.8, 1H), 7.81 (t, *J*=1.9, 1H), 7.62 (t, *J*=7.9, 1H), 7.56 (s, 1H), 7.53 (d, *J*=5.3, 1H), 7.46 (dd, *J*=8.1, 2.3, 1H), 7.34 (dd, *J*=8.6, 2.7, 1H), 7.05 (d, *J*=8.7, 1H). ^13^C NMR (126 MHz, DMSO-*d_6_*) *δ* 164.5, 163.5, 153.2, 150.0, 149.4, 141.7, 140.5 (q, *J*=33.5), 135.8, 130.1, 126.3, 125.4, 125.1, 124.4, 120.7, 119.6, 118.7, 116.4, 114.7 (d, *J*=3.2), 108.2 (d, *J*=4.0). HRMS (ESI) *m/z* calcd for C_21_H_15_F_3_N_5_O_3_ [*M*+H]^+^: 442.1122, found: 442.1116.

***N*-(5-((*di*Ethylamino)methyl)-2-hydroxyphenyl)-3-((4-(trifluoromethyl)pyridin-2-yl)oxy)benzamide (27)**

Following GP3, **27a** (0.145 g, 0.306 mmol, 1.0 equiv) and BBr_3_ (1.53 mmol, 1.53 mL, 5.0 equiv) in DCM (3mL) afforded after prep HPLC **27** as light yellow solid (0.100 g, 71%). ^1^H NMR (500 MHz, DMSO-*d_6_*) *δ* 9.61 (s, 1H), 8.43 (d, *J*=5.2 Hz, 1H), 7.88 (dt, *J*=7.8, 1.4 Hz, 1H), 7.79 (t, *J* = 2.1 Hz, 1H), 7.60 (t, *J* = 8.0 Hz, 1H), 7.56 – 7.51 (m, 3H), 7.44 (ddd, *J* = 8.1, 2.5, 1.0 Hz, 1H), 6.97 (dd, *J* = 8.2, 2.1 Hz, 1H), 6.85 (d, *J* = 8.2 Hz, 1H), 3.43 (s, 2H), 2.44 (q, *J* = 7.1 Hz, 4H), 0.96 (t, *J* = 7.1 Hz, 6H). ^13^C NMR (126 MHz, DMSO-*d_6_*) *δ* 164.3, 163.5, 153.2, 149.4, 148.5, 140.46 (q, *J* = 33.5 Hz), 136.1, 130.1, 130.0, 126.3, 125.2, 124.9, 124.9, 124.3, 122.5 (q, *J* = 273.4 Hz), 120.7, 115.7, 114.7(q, *J* = 3.4 Hz), 108.1 (q, *J* = 4.1 Hz), 56.4, 45.9, 11.6. HRMS (ESI) *m/z* calcd for C_24_H_25_F_3_N_3_O_3_ [*M*+H]^+^: 460.1843, found: 460.1833.

***N*-(5-(((3-(*di*Ethylamino)propyl)amino)methyl)-2-hydroxyphenyl)-3-((4-(trifluoromethyl)pyridin-2-yl)oxy)benzamide (28)**

Following GP3, **28a** (0.070 g, 0.131 mmol, 1.0 equiv) and BBr_3_ (0.66 mmol, 0.66 mL, 5.0 equiv) in DCM (2 mL) afforded **28** as white powder after prep HPLC (0.035 g, 52%). ^1^H NMR (500 MHz, Acetone-*d_6_*) *δ* 8.45 (s, 1H), 8.36 (d, *J* = 5.3 Hz, 1H), 8.08 (s, 1H), 7.94 (d, *J* = 7.9 Hz, 1H), 7.88 (s, 1H), 7.59 (t, *J* = 7.9 Hz, 1H), 7.42 (t, *J* = 5.3 Hz, 2H), 7.37 (s, 1H), 7.09 (d, *J* = 7.9 Hz, 1H), 6.95 (d, *J* = 7.7 Hz, 1H), 4.88 (br, 2H), 3.95 (s, 2H), 3.01 (t, *J* = 6.1 Hz, 2H), 2.81(t, *J* = 6.2 Hz, 2H), 2.71 (q, *J* = 7.2 Hz, 4H), 1.90 (s, 2H), 1.03 (t, *J* = 7.0 Hz, 6H). ^13^C NMR (126 MHz, Acetone) *δ* 167.6, 165.6, 164.9, 154.8, 150.2, 149.8, 142.1 (q, *J* = 33.9 Hz), 137.2, 130.9, 127.6, 127.3, 126.0, 125.0, 123.8, 121. 8, 117.6, 115.3 (q, *J* = 3.3 Hz), 109.0 (q, *J* = 3.9 Hz), 52.2, 51.8, 47.6, 47.0, 23.5, 10.7. HRMS (ESI) *m/z* calcd for C_27_H_32_F_3_N_4_O_3_ [*M*+H]^+^: 517.2421, found: 517.2415.

***N*-(5-((Cyclohexyl(methyl)amino)methyl)-2-hydroxyphenyl)-3-((4-(trifluoromethyl)pyridin-2-yl)oxy)benzamide (29)**

Following GP3, **29a** (0.090 g, 0.17 mmol, 1.0 equiv) and BBr_3_ (0.87 mmol, 0.87 mL, 5.0 equiv) in DCM (2 mL) afforded **29** as a white powder after prep HPLC (0.065 g, 76%). ^1^H NMR (500 MHz, DMSO-*d_6_*) *δ* 9.61 (s, 1H), 9.54 (s, 1H), 8.42 (d, *J* = 5.2 Hz, 1H), 7.88 (dt, *J* = 7.8, 1.4 Hz, 1H), 7.79 (t, *J* = 2.1 Hz, 1H), 7.60 (t, *J* = 7.9 Hz, 1H), 7.55 (s, 1H), 7.54 – 7.51 (m, 2H), 7.44 (ddd, *J* = 8.1, 2.4, 0.9 Hz, 1H), 6.95 (dd, *J* = 8.2, 2.1 Hz, 1H), 6.84 (d, *J* = 8.2 Hz, 1H), 3.44 (s, 2H), 2.38 (td, *J* = 9.3, 8.0, 5.5 Hz, 1H), 2.08 (s, 3H), 1.75 (t, *J* = 13.7 Hz, 4H), 1.57 (d, *J* = 12.6 Hz, 1H), 1.25 – 1.11 (m, 5H). ^13^C NMR (126 MHz, DMSO-*d_6_*) *δ* 164.3, 163.5, 153.2, 149.4, 148.4, 140.4 (q, *J* = 33.5 Hz), 136.1, 130.8, 130.0, 126.1, 125.2, 124.8, 124.7, 124.3, 122.58 (q, *J* = 273.2 Hz), 120.7, 115.7, 114.64 (q, *J* = 3.4 Hz), 108.1 (q, *J*=4.0 Hz), 61.5, 57.0, 36.9, 28.1, 26.0, 25.5. HRMS (ESI) *m/z* calcd for C_27_H_29_F_3_N_3_O_3_ [*M*+H]^+^: 500.2156, found: 500.2141.

***N*-(2-Hydroxy-5-(morpholinomethyl)phenyl)-3-((4-(trifluoromethyl)pyridin-2-yl)oxy)benzamide (30)**

Following GP3, **30a** (0.048 g, 0.098 mmol, 1.0 equiv) and BBr_3_ (0.49 mL, 0.049 mmol, 5.0 equiv) in DCM (1 mL) afforded as white powder after prep HPLC **30** (0.030 g, 65%). ^1^H NMR (500 MHz, MeOD-*d_4_*) *δ* 8.34(d, *J* = 5.2 Hz, 1H), 7.84 (d, *J* = 7.8 Hz, 1H), 7.81 – 7.74 (m, 2H), 7.60 (t, *J* = 7.9 Hz, 1H), 7.44 – 7.38 (m, 2H), 7.36 (s, 1H), 7.03 (dd, *J* = 8.2, 2.1 Hz, 1H), 6.87 (d, *J* = 8.2 Hz, 1H), 3.67 (t, *J* = 4.7 Hz, 4H), 3.46 (s, 2H), 2.47 (d, *J* = 4.7 Hz, 4H). ^13^C NMR (126 MHz, DMSO-*d_6_*) *δ* 164.3, 163.5, 153.2, 149.5, 148.9, 140.50 (q, *J* = 33.2 Hz), 136.1, 130.1, 128.1, 126.8, 125.4, 125.2, 124.4, 124.4, 123.7 (q, *J* = 273.7 Hz), 120.7, 115.7, 114.7 (q, *J* = 3.4 Hz), 108.2(q, *J* = 3.8 Hz), 66.2, 62.1, 53.1. HRMS (ESI) *m/z* calcd for C_24_H_21_F_3_N_3_O_4_ [*M*-H]^-^: 472.1490, found: 472.1478.

***N*-(2-hydroxy-5-((4-methylpiperazin-1-yl)methyl)phenyl)-3-((4-(trifluoromethyl)pyridin-2-yl)oxy)benzamide (31)**

Following GP3, 31a (0.055 g, 0.11 mmol, 1.0 equiv) and BBr_3_ (0.55 mL, 0.55 mmol, 5.0 equiv) in DCM (1 mL) afforded as white powder after prep HPLC **31** (0.035 g, 66%). ^1^H NMR (500 MHz, Acetone-*d_6_*) *δ* 9.55 (s, 1H), 8.39 (d, *J* = 5.2 Hz, 1H), 7.99 – 7.95 (m, 1H), 7.90 (t, *J* = 2.1 Hz, 1H), 7.69 – 7.62 (m, 2H), 7.50 – 7.43 (m, 2H), 7.41 (s, 1H), 7.04 (dd, *J* = 8.2, 2.0 Hz, 1H), 6.91 (d, *J* = 8.2 Hz, 1H), 3.39 (s, 2H), 2.39 (s, 8H), 2.19 (s, 3H). ^13^C NMR (126 MHz, Acetone-*d_6_*) *δ* 166.4, 166.3, 164.9, 154.9, 150.3, 148.8, 148.7, 142.27 (q, *J*=33.9), 136.9, 136.8, 131.3, 131.0, 127.7, 127.2, 127.2, 127.7, 126.3, 125.2, 124.17, 124.3, 123.70 (q, J = 272.5 Hz), 121.9, 118.3, 115.5 (q, *J*=3.3), 109.1 (q, *J*=4.0). 63.0, 56.0, 53.7, 46.3. HRMS (ESI) *m/z* calcd for C_25_H_26_F_3_N_4_O_3_ [*M*+H]^+^: 487.1952, found: 487.1946.

***N*-(3-Fluoro-2-hydroxy-5-(morpholinomethyl)phenyl)-3-((4-(trifluoromethyl)pyridin-2-yl)oxy)benzamide (32)**

Following GP3, **32a** (0.070 g, 0.13 mmol, 1.0 equiv) and BBr_3_ (0.69 mL, 0.69 mmol, 5.0 equiv) in DCM (1 mL) afforded as white powder after prep HPLC **32** as white powder (0.010 g, 15%). ^1^H NMR (500 MHz, Acetone-*d_6_*) *δ* 9.74 (s, 1H), 8.40 (d, *J* = 5.2 Hz, 1H), 7.99 (d, *J*=7.7, 1H), 7.91 (t, *J* = 2.1 Hz, 1H), 7.66 (t, *J* = 7.9 Hz, 1H), 7.52-7.48 (m, 2H), 7.46 (d, *J* = 5.3 Hz, 1H), 7.42 (s, 1H), 7.02 (d, *J* = 11.2 Hz, 1H), 3.62 (m, 4H), 3.45 (s, 2H), 2.42 (s, 4H). ^13^C NMR (126 MHz, Acetone-*d_6_*) *δ* 166.8, 164.9, 154.9, 154.7, 152.8, 150.3, 142.29 (q, *J*=33.8), 137.5, 136.4, 131.1, 129.17 (d, *J*=4.5), 126.5, 125.4, 122.1, 119.7, 115.52 (q, *J*=3.3), 114.03 (d, *J*=19.6), 109.09 (q, *J*=4.0).67.4, 63.0, 54.4. HRMS (ESI) *m/z* calcd for C_24_H_22_F_4_N_3_O_4_ [*M*+H]^+^: 492.1541, found: 492.1535.

***N*-(3-Chloro-2-hydroxy-5-(morpholinomethyl)phenyl)-3-((4-(trifluoromethyl)pyridin-2-yl)oxy)benzamide (33)**

Following GP3, **33a** (0.200 g, 0.38 mmol, 1.0 equiv) and BBr_3_ (1.92 mL, 1.92 mmol, 5.0 equiv) in DCM (2 mL) afforded as white powder after prep HPLC **33** (0.068 g, 35%) as white powder. ^1^H NMR (500 MHz, DMSO-*d_6_*) *δ* 10.04 (s, 1H), 9.68 (s, 1H), 8.42 (d, *J*=5.3 Hz, 1H), 7.91 (d, *J*=7.7 Hz, 1H), 7.82 (d, *J*=2.0 Hz, 1H), 7.62 (t, *J*=7.9 Hz, 1H), 7.58 (s, 1H), 7.54 (d, *J*=5.2 Hz, 1H), 7.46 (dd, *J*=8.2, 2.4 Hz, 1H), 7.35 (d, *J*=2.1 Hz, 1H), 7.19 (d, *J*=1.9 Hz, 1H), 3.56 (t, *J*=4.5 Hz, 4H), 3.37 (s, 2H), 2.34 (s, 4H). ^13^C NMR (126 MHz, DMSO-*d_6_*) *δ* 165.1, 163.5, 153.1, 149.5, 145.8, 140.5 (q, *J*=33.5 Hz), 135.6, 130.0, 129.4, 127.3, 127.0, 125.3, 125.2, 124.8, 122.6 (q, *J*=273.4 Hz), 121.2, 121.1, 114.7 (q, *J*=3.4 Hz), 108.1 (q, *J*=4.0 Hz), 66.2, 61.3, 53.0. HRMS (ESI) *m/z* calcd for C_24_H_22_ClF_3_N_3_O_4_ [*M*+H]^+^: 508.1245, found: 508.1234.

***N*-(2-Fluoro-5-(morpholinomethyl)phenyl)-3-((4-(trifluoromethyl)pyridin-2-yl)oxy)benzamide (34)**

Following GP2, **41a** (0.100 g, 0.331 mmol, 1.3 equiv), Na_2_CO_3_ (0.033 g, 0.37 mmol, 1.5 equiv) 53 (0.053 g, 0.25 mmol, 1.0 equiv) in dry DMF (3 mL) afforded after prep HPLC **34** as white powder (0.027 g, 17%). ^1^H NMR (500 MHz, Acetone-*d_6_*) *δ* 9.31 (s, 1H), 8.39 (d, *J*=5.2 Hz, 1H), 8.01 (dd, *J*=7.7, 2.0 Hz, 1H), 7.94 (dt, *J*=7.8, 1.3 Hz, 1H), 7.86 (t, *J*=2.1 Hz, 1H), 7.63 (t, *J*= 7.9 Hz, 1H), 7.48 – 7.43 (m, 2H), 7.41 (s, 1H), 7.20 – 7.13 (m, 2H), 3.61 (t, *J*=4.6 Hz, 4H), 3.48 (s, 2H), 2.47 – 2.34 (m, 4H). ^13^C NMR (126 MHz, Acetone-*d_6_*) *δ* 165.4, 164.9, 155.7, 154.7, 153.8, 150.2, 142.1 (q, *J*=33.9 Hz), 137.3, 135.40 (d, *J*=3.6 Hz), 130.8, 127.1 (d, *J*=7.5 Hz), 126.8 (d, *J*=11.8 Hz), 126.3 (d, *J* =1.6 Hz), 126.0, 125.2, 123.68 (q, *J*=272.5 Hz), 121.8, 115.8 (d, *J*=19.9 Hz), 115.3 (q, *J*=3.3 Hz), 108.9 (q, *J*=4.1 Hz), 67.5, 63.2, 54.4. HRMS (ESI) *m/z* calcd for C_24_H_22_F_4_N_3_O_3_ [*M*+H]^+^: 476.1592, found: 476.1585.

***N*-(4-(Morpholinomethyl)pyridin-2-yl)-3-((4-(trifluoromethyl)pyridin-2-yl)oxy)benzamide (35)**

Following GP2, **41a** (0.100 g, 0.331 mmol, 1.3 equiv), Na_2_CO_3_ (0.033 g, 0.37 mmol, 1.5 equiv) **55** (0.053 g, 0.25 mmol, 1.0 equiv) in dry DMF (3 mL) afforded after prep HPLC **35** (0.010 g, 10%). ^1^H NMR (500 MHz, Acetone- *d_6_*) *δ* 9.68 (s, 1H), 8.39 (d, *J* = 5.2 Hz, 1H), 8.35 (s, 1H), 8.24 (d, *J*=5.0 Hz, 1H), 8.03 (dt, *J* = 7.8, 1.3 Hz, 1H), 7.96 (t, *J*=2.1 Hz, 1H), 7.64 (t, *J*=7.9 Hz, 1H), 7.50 – 7.43 (m, 2H), 7.41 (s, 1H), 7.14 (dd, *J*=5.1, 1.4 Hz, 1H), 3.64 (t, *J*=4.6 Hz, 4H), 3.56 (s, 2H), 2.44 (t, *J* = 4.6 Hz, 4H). ^13^C NMR (126 MHz, Acetone- *d_6_*) *δ* 165.6, 164.9, 154.8, 153.4, 150.9, 150.2, 148.8, 142.1 (q, *J*=33.8), 137.2, 130.8, 126.1, 125.3, 123.6 (q, *J*=272.5 Hz), 121.9, 121.0, 115.4 (q, *J* = 3.4 Hz), 115.0, 109.0 (q, *J*=4.1 Hz), 67.4, 62.9, 54.5. HRMS (ESI) *m/z* calcd for C_23_H_22_F_3_N_4_O_3_ [*M*+H]^+^: 459.1639, found: 459.1652.

**4-(Morpholinomethyl)-2-((3-((4-(trifluoromethyl)pyridin-2-yl)oxy)benzyl)amino)phenol (36)**

Following GP3, **36a** (0.300 g, 0.64 mmol, 1.0 equiv) and BBr_3_ (3.17 mL, 3.17 mmol, 5.0 equiv) in DCM (2 mL) afforded after prep **36** as white powder (0.074 g, 25%). ^1^H NMR (500 MHz, DMSO-*d_6_*) *δ* 8.37 (d, *J*=5.2 Hz, 1H), 8.33 (s, br 1H), 7.47 (dd, *J* = 5.2, 1.5 Hz, 1H), 7.41 (d, *J*=1.4 Hz, 1H), 7.36 (t, *J*=7.8 Hz, 1H), 7.22 (dt, *J*=7.8, 1.3 Hz, 1H), 7.11 (t, *J*=2.0 Hz, 1H), 7.02 (ddd, *J*= 8.0, 2.5, 1.0 Hz, 1H), 6.58 (d, *J*=7.8 Hz, 1H), 6.33 (d, *J*=1.9 Hz, 1H), 6.28 (dd, *J*=7.9, 1.9 Hz, 1H), 5.35 (s, 1H), 4.32 (s, 2H), 3.44 (t, *J*=4.6 Hz, 4H), 3.18 (s, 2H), 2.17 (s, 4H). ^13^C NMR (126 MHz, DMSO-*d_6_*) *δ* 163.7, 153.3, 149.5, 143.1, 143.3, 140.5 (q, *J*=33.7 Hz), 136.5, 129.6, 128.2, 123.9, 122.62 (q, *J*= 273.5 Hz), 119.9, 119.6, 116.5 114.3 (q, *J*=3.6 Hz), 113.0, 111.1, 107.8 (q, *J*=3.8 Hz), 66.2, 62.7, 53.0, 46.1. HRMS (ESI) *m/z* calcd for C_24_H_25_F_3_N_3_O_3_ [*M*+H]^+^: 460.1843, found: 460.1835.

**4-(Morpholinomethyl)-2-(4-(3-((4-(trifluoromethyl)pyridin-2-yl)oxy)phenyl)-1*H*-1,2,3-triazol-1-yl)phenol (37)**

Following GP3, **37a** (0.020 g, 0.039 mmol, 1.0 equiv) and BBr_3_ (0.19 mL, 0.19 mmol, 5.0 equiv) in DCM (2 mL) afforded as white powder after prep **37** (0.010 g, 52%). ^1^H NMR (500 MHz, Acetone-*d_6_*) *δ* 8.93 (s, 1H), 8.40 (d, *J*=5.2 Hz, 1H), 7.91 (dt, *J*= 7.8, 1.3 Hz, 1H), 7.84 (dd, *J*=2.4, 1.6 Hz, 1H), 7.75 (d, *J*= 2.1 Hz, 1H), 7.56 (t, *J*=7.9 Hz, 1H), 7.43 (dd, *J*=5.3, 1.5 Hz, 1H), 7.38 (dt, *J*= 1.6, 0.8 Hz, 1H), 7.33 (dd, *J*=8.3, 2.1 Hz, 1H), 7.21 (ddd, *J*= 8.1, 2.4, 1.0 Hz, 1H), 7.16 (d, *J*=8.4 Hz, 1H), 3.61 (t, *J*= 4.6 Hz, 4H), 3.50 (s, 2H), 2.42 (s, 4H). ^13^C NMR (126 MHz, Acetone-*d_6_*) *δ* 165.2, 155.3, 150.4, 149.3, 147.1, 143.04 (q, *J*=33.8),133.8, 131.5, 131.5, 131.2, 125.33, 125.20, 124.74 (q, *J*=272.5), 123.2, 122.9, 122.1, 119.6, 118.3, 115.2 (q, *J*=3.4), 108.8 (q, *J*=4.0), 67.5, 62.9, 54.5. HRMS (ESI) *m/z* calcd for C_25_H_23_F_3_N_5_O_3_ [*M*+H]^+^: 498.1748, found: 498.1735.

**4-Methoxy-3-(3-phenoxybenzamido)benzenesulfonic acid (2a)**

To a stirred solution of 3-amino-4-methoxybenzenesulfonic acid (0.134 g, 0.66 mmol, 1.0 equiv.) in DMF (4 mL, 0.2 M), Na_2_CO_3_ (0.084 g, 0.8 mmol, 1.5 equiv.) was added and the reaction mixture was left stirring for 30 min. At 0 °C a freshly prepared 3-phenoxybenzoyl chloride (0.200 g, 0.86, 1.3 equiv.) was added and the resulting mixture was stirred at RT on. Then, the pH was adjusted to acidic by the addition of HCl 2N and the aqueous layer was extracted with DCM (3x10 mL). The combined organic layers were dried over Na_2_SO_4_, filtered, concentrated in vacuo to afford **2a** without any further purification (0.115 g, 44%). ^1^H NMR (500 MHz, DMSO-*d_6_*) *δ* 9.52 (s, 1H), 7.89 (s, 1H), 7.74 (d, *J*=7.4 Hz, 1H), 7.59 – 7.49 (m, 2H), 7.47 – 7.37 (m, 3H), 7.25 – 7.15 (m, 2H), 7.09 (d, *J*=8.5 Hz, 2H), 7.00 (dd, *J*=8.5, 2.9 Hz, 1H), 3.80 (s, *J*=2.9 Hz, 3H).

***N*-(5-(*N*-Butylsulfamoyl)-2-methoxyphenyl)-3-phenoxybenzamide (8a)**

Following GP2, **2b** (0.125 g, 0.300 mmol), butylamine (0.017 g, 0.23 mmol), Na_2_CO_3_ (0.037 g, 0.35 mmol) in DCM (2 mL) afforded after flash chromatography (Cyclohexane/EtOAc 2:1) **8a** as white powder (0.078 g, 75%). ^1^H NMR (500 MHz, DMSO-*d_6_*) *δ* 9.70 (s, 1H), 8.19 (s, 1H), 7.74 (d, *J*=7.5 Hz, 1H), 7.61 (d, *J*=8.6 Hz, 1H), 7.54 (m, 2H), 7.44 (m, 3H), 7.26 (dd, *J*=10.7, 8.9 Hz, 2H), 7.20 (t, *J*=7.5 Hz, 1H), 7.09 (d, *J*=8.2 Hz, 2H), 3.91 (s, 3H), 2.71 (t, *J*=6.8 Hz, 2H), 1.39 – 1.30 (m, 2H), 1.28 – 1.18 (m, 2H), 0.80 (t, *J*= 7.3 Hz, 3H).

***N*-(5-((3,3-*di*Methylpyrrolidin-1-yl)sulfonyl)-2-methoxyphenyl)-3-phenoxybenzamide (10a)**

Following GP2, **2b** (0.150 g, 0.300 mmol), 3,3-dimethylpyrrolidine (0.022 g, 0.23 mmol), Na_2_CO_3_ (0.037 g, 0.35 mmol) in DCM (2 mL) afforded after flash chromatography (Cyclohexane/EtOAc 2:1) **10a** as white powder (0.035 g, 32%). ^1^H NMR (500 MHz, DMSO-*d_6_*) *δ* 9.73 (s, 1H), 8.22 (s, 1H), 7.75 (d, *J*=7.5, 1H), 7.63 (d, *J*=8.5, 1H), 7.58 – 7.52 (m, 2H), 7.44 (t, *J*=7.3, 2H), 7.30 (d, *J*=8.5, 1H), 7.25 (d, *J*=8.0, 1H), 7.20 (t, *J*=7.2, 1H), 7.09 (d, *J*=7.7, 2H), 3.92 (s, 3H), 3.24 (t, *J*=6.4, 2H), 2.91 (s, 2H), 1.52 (t, *J*=6.4, 2H), 0.83 (s, 6H).

***N*-(2-Methoxy-5-((4-methylpiperidin-1-yl)sulfonyl)phenyl)-3-phenoxybenzamide (11a)**

Following GP2, **2b** (0.138 g, 0.330 mmol), 4-methylpiperidine (0.025 g, 0.25 mmol), Na_2_CO_3_ (0.039 g, 0.37 mmol) in DCM (2 mL) afforded after flash chromatography (Cyclohexane/EtOAc 2:1) **11a** as white powder (0.042 g, 35%).^1^H NMR (500 MHz, DMSO-*d_6_*) *δ* 9.73 (s, 1H), 8.14 (d, *J*=1.5, 1H), 7.75 (d, *J*=7.7, 1H), 7.56 (ddd, *J*=7.6, 4.8, 2.6, 3H), 7.45 (t, *J*=7.8, 2H), 7.32 (d, *J*=8.7, 1H), 7.26 (dd, *J*=8.2, 2.4, 1H), 7.21 (t, *J*=7.4, 1H), 7.09 (d, *J*=8.4, 2H), 3.93 (s, 3H), 3.58 (d, *J*=11.6, 2H), 2.20 (t, *J*=11.1, 2H), 1.66 (d, *J*=11.2, 2H), 1.35 – 1.25 (m, 1H), 1.14 (qd, *J*=12.4, 3.8, 2H), 0.86 (d, *J*=6.4, 3H).

***N*-(5-(*N*-(Cyclohexylmethyl)sulfamoyl)-2-methoxyphenyl)-3-phenoxybenzamide (13a)**

Following GP2, **2b** (0.150 g, 0.360 mmol), cyclohexylmethanamine (0.031 g, 0.27 mmol), Na_2_CO_3_ (0.043 g, 0.40 mmol) in DCM (2 mL) afforded after flash chromatography (Cyclohexane/EtOAc 2:1) **13a** as white powder (0.095 g, 71%). ^1^H NMR (500 MHz, DMSO-*d_6_*) *δ* 9.69 (s, 1H), 8.18 (s, 1H), 7.74 (d, *J*=7.6, 1H), 7.60 (d, *J*=8.7, 1H), 7.55 (d, *J*=8.7, 2H), 7.45 (dd, *J*=17.0, 8.4, 3H), 7.26 (t, *J*=8.1, 2H), 7.20 (t, *J*=7.1, 1H), 7.09 (d, *J*=7.7, 2H), 3.90 (s, 3H), 2.53 (d, *J*=7.2, 2H), 1.71 – 1.52 (m,6H), 1.40 – 1.26 (m, 1H), 1.20 – 1.03 (m, 4H).

***N*-(5-(*N*-Benzylsulfamoyl)-2-methoxyphenyl)-3-phenoxybenzamide (14a)**

Following GP2, **2b** (0.075 g, 0.180 mmol), benzylamine (0.015 g, 0.14 mmol), Na_2_CO_3_ (0.022 g, 0.21 mmol) in DCM (2 mL) afforded after flash chromatography (Cyclohexane/EtOAc 1:2) **14a** as white powder (0.032 g, 47%). ^1^H NMR (500 MHz, DMSO-*d_6_*) *δ* 9.84 – 9.52 (m, 1H), 8.23 (d, *J*=2.3 Hz, 1H), 8.05 (s, 1H), 7.75 (dd, *J*=7.8, 1.5 Hz, 1H), 7.63 (dd, *J*=8.6, 2.4 Hz, 1H), 7.58 – 7.54 (m, 2H), 7.46 – 7.41 (m, 2H), 7.30 – 7.18 (m, 8H), 7.09 (dt, *J*=7.6, 1.0 Hz, 2H), 3.95 (s, 2H), 3.91 (s, 3H).

***N*-(5-((diEthylamino)methyl)-2-methoxyphenyl)-3-((4-(trifluoromethyl)pyridin-2-yl)oxy)benzamide (27a)**

Following GP4, **43** (0.180 g, 0.43 mmol, 1.0 equiv), diethylamine (0.041 g, 0.56 mmol, 1.3 equiv), STAB (0.145 g, 0.7 mmol, 1.6 equiv) in DCE afforded after flash chromatography (DCM/MeOH, product at 8% of MeOH) **27a** as a yellowish oil (0.160 g, 76%). ^1^H NMR (500 MHz, DMSO-*d_6_*) *δ* 9.53 (s, 1H), 8.42 (d, *J*=5.1 Hz, 1H), 7.86 (d, *J*=7.6 Hz, 1H), 7.77 (s, 1H), 7.66 – 7.58 (m, 2H), 7.57 (s, 1H), 7.54 (d, *J*=5.1, 1H), 7.43 (d, *J*=8.0 Hz, 1H), 7.11 (d, *J*=8.3 Hz, 1H), 7.01 (d, *J*=8.4 Hz, 1H), 3.79 (s, 3H), 2.44 (dd, *J*=14.1, 7.1, 4H), 0.96 (t, *J*=7.1 Hz, 6H).

***N*-(5-(((3-(*di*Ethylamino)propyl)amino)methyl)-2-methoxyphenyl)-3-((4-(trifluoromethyl)pyridin-2-yl)oxy)benzamide (28a)**

Following GP4, **43** (0.120 g, 0.288 mmol, 1.0 equiv), *N*,*N*-diethylpropane-1,3-diamine (0.048 g, 0.37 mmol, 1,3 equiv) and STAB (0.097 g, 0.46 mmol, 1.6 equiv) in DCE (3 mL) afforded after column chromatography (DCM/MeOH and NH_3_ 0.5%, product at 10% of MeOH) **28a** as slight yellow oil (0.07 g, 46%). ^1^H NMR (500 MHz, DMSO-*d_6_*) *δ* 9.54 (s, 1H), 8.43 (d, *J*=5.2 Hz, 1H), 7.64 (d, *J*=1.4, 1H), 7.60 (t, *J*=7.9 Hz, 1H), 7.57 (s, 1H), 7.54 (dd, *J*=5.2, 0.9 Hz, 1H), 7.44 (dd, *J*=8.1, 1.5 Hz, 1H), 7.13 (dd, *J*=8.3, 2.0, 1H), 7.02 (d, *J*=8.4, 1H), 3.79 (s, 3H), 3.68 (d, *J*=39.2 Hz, 2H), 3.08 – 2.97 (m, 1H), 2.44 – 2.38 (m, 4H), 1.56 – 1.49 (m, 2H), 1.18 – 1.25 (m, 4H), 0.93 – 0.88 (m, 6H).

***N*-(5-((Cyclohexyl(methyl)amino)methyl)-2-methoxyphenyl)-3-((4-(trifluoromethyl)pyridin-2-yl)oxy)benzamide (29a)**

Following GP4, **43** (0.100 g, 0.24 mmol, 1.0 equiv), *N*-methylcyclohexanamine (0.035 g, 0.31 mmol, 1.3 equiv) and STAB (0.081 g, 0.38 mmol, 1.6 equiv) in DCE (1.5 mL) afforded after flash chromatography (DCM/MeOH, product at 10% of MeOH **29a** as white solid (74%, 0.091 g). ^1^H NMR (500 MHz, DMSO-*d_6_*) *δ* = 9.53 (s, 1H), 8.43 (d, *J*=4.7, 1H), 7.86 (d, *J*=7.5 Hz, 1H), 7.77 (s, 1H), 7.68 – 7.56 (m, 3H), 7.53 (s, 1H), 7.43 (d, *J*=8.0 Hz, 1H), 7.10 (d, *J*=7.4 Hz, 1H), 7.01 (d, *J*=8.4 Hz, 1H), 3.80 (s, 2H), 2.09 (s, 3H), 1.88-1.97 (m, 3H), 1.69-1.85 (m, 4H), 1.57 (m, 1H), 1.43 – 1.01 (m, 6H).

***N*-(2-Methoxy-5-(morpholinomethyl)phenyl)-3-((4-(trifluoromethyl)pyridin-2-yl)oxy)benzamide (30a)**

Following GP4, **43** (0.125 g, 0.300 mmol, 1.0 equiv), morpholine (0.034 g, 0.39 mmol, 1.3 equiv), STAB (0.101 g, 0.48 mmol, 1.6 equiv) in DCE (2 mL) afforded after column chromatography (DCM/MeOH, product at 10% of MeOH) **30a** as slight yellow oil (0.09 g, 60%). ^1^H NMR (500 MHz, DMSO-*d_6_*) *δ* 9.52 (s, 1H), 8.43 (d, *J*=5.2, 1H), 7.86 (d, *J*=7.7, 1H), 7.77 (s, 1H), 7.64 (s, 1H), 7.60 (t, *J*=7.9, 1H), 7.56 (s, 1H), 7.53 (d, *J*=5.2, 1H), 7.44 (dd, *J*=7.9, 1.9, 1H), 7.11 (dd, *J*=8.4, 1.9, 1H), 7.03 (d, *J*=8.4, 1H), 3.80 (s, 3H), 3.59 – 3.54 (m, 4H), 3.40 (s, 2H), 2.34 (s, 4H).

***N*-(2-Methoxy-5-((4-methylpiperazin-1-yl)methyl)phenyl)-3-((4-(trifluoromethyl)pyridin-2-yl)oxy)benzamide (31a)**

Following GP4, **43** (0.049 g, 0.117 mmol, 1.0 equiv), methyl-piperazine (0.015 g, 0.153 mmol, 1.3 equiv), STAB (0.040 g, 0.187 mmol, 1.6 equiv) in DCE (5 mL) afforded after column chromatography (DCM/MeOH, product at 7% of MeOH) **31a** as a colourless oil (0.055 g, 94%). ^1^H NMR (500 MHz, Acetone) *δ* 8.88 (s, 1H), 8.40 (d, *J*=5.2, 1H), 8.34 (d, *J*=1.6, 1H), 7.87 (d, *J*=7.7, 1H), 7.84 – 7.79 (m, 1H), 7.62 (t, *J*=7.9, 1H), 7.45 (dd, *J*=7.5, 1.7, 2H), 7.41 (s, 1H), 7.06 (d, *J*=8.3, 1H), 7.00 (d, *J*=8.3, 1H), 3.90 (s, 3H), 3.44 (s, 2H), 2.60-2.25 (m, 8H), 2.20 (s, 3H).

***N*-(3-Fluoro-2-methoxy-5-(morpholinomethyl)phenyl)-3-((4-(trifluoromethyl)pyridin-2-yl)oxy)benzamide (32a)**

Following GP2, **41a** (0.210 g, 0.69 mmol, 1.3 equiv) in dry DMF (0.2 M), Na_2_CO_3_ (0.085 g, 0.79 mmol, 1.5 equiv) and **46** (0.129 g, 0.53 mmol, 1.0 equiv) afforded after flash chromatography (DCM/MeOH, product at 5% of MeOH) **32a** as white powder (0.090 g, 34%). ^1^H NMR (500 MHz, Acetone-*d_6_*) *δ* 9.05 (s, 1H), 8.40 (d, *J*=5.2 Hz, 1H), 8.13 (s, 1H), 7.87 (dd, *J*=7.8, 1.6 Hz, 1H), 7.82 (t, *J*=2.0 Hz, 1H), 7.63 (t, *J*=7.9 Hz, 1H), 7.48 – 7.45 (m, 2H), 7.41 (s, 1H), 6.98 (dd, *J*=12.4, 2.0 Hz, 1H), 3.98 (d, *J*= 1.7 Hz, 3H), 3.63 (t, *J*=4.6 Hz, 4H), 3.47 (s, 2H), 2.42 (t, *J*=4.6 Hz, 4H).

***N*-(3-Chloro-2-methoxy-5-(morpholinomethyl)phenyl)-3-((4-(trifluoromethyl)pyridin-2-yl)oxy)benzamide (33a)**

Following GP2, **41a** (0.213 g, 0.71 mmol, 1.3 equiv), Na_2_CO_3_ (0.086 g, 0.81 mmol, 1.5 equiv) **51** (0.139 g, 0.54 mmol, 1.0 equiv) in dry DMF (3 mL) afforded after flash chromatography (DCM/MeOH, product at 5% of MeOH) **33a** as white powder (0.20 g, 38%). ^1^H NMR (500 MHz, DMSO-*d_6_*) *δ* 9.91 (s, 1H), 8.43 (d, *J*=5.2, 1H), 7.88 (d, *J*=7.9, 1H), 7.82 – 7.74 (m, 1H), 7.65 – 7.61 (m, 1H), 7.60 (s, 1H), 7.57 (s, 1H), 7.54 (d, *J*=5.3, 1H), 7.46 (dd, *J*=8.1, 1.6, 1H), 7.28 (d, *J*=1.9, 1H), 3.75 (s, 3H), 3.62 – 3.53 (m, 4H), 2.44 – 2.29 (m, 4H).

**2-Methoxy-5-(morpholinomethyl)-*N*-(3-((4-(trifluoromethyl)pyridin-2-yl)oxy)benzyl)aniline (36a)**

Following GP4, **59** (0.400 g, 2.0 mmol, 1.0 equiv), **57** (0.23 g, 2.6 mmol, 1.3 equiv), STAB (0.68 g, 3.2 mmol, 1.6 equiv) in DCE (2 mL) afforded after column chromatography (DCM/MeOH, product at 4% of MeOH) **36a** as a white solid (0.400 g, 42%). ^1^H NMR (500 MHz, DMSO-*d_6_*) *δ* 8.37 (d, *J*= 5.1 Hz, 1H), 7.47 (dd, *J*=5.3, 1.5 Hz, 1H), 7.37 (dd, *J*=16.1, 8.3 Hz, 2H), 7.21 (dt, *J* = 7.6, 1.3 Hz, 1H), 7.10 (t, *J*=2.0 Hz, 1H), 7.02 (ddd, *J*=8.1, 2.6, 1.0 Hz, 1H), 6.72 (d, *J*=8.0 Hz, 1H), 6.42 (dd, *J* = 8.0, 1.9 Hz, 1H), 6.37 (d, *J*=1.9 Hz, 1H), 5.59 (t, *J*=6.3 Hz, 1H), 4.34 (d, *J*=6.3 Hz, 2H), 3.76 (s, 3H), 3.44 (t, *J*=4.6 Hz, 4H), 3.22 (s, 2H), 2.18 (d, *J*=4.6 Hz, 4H).

**4-(4-Methoxy-3-(4-(3-((4-(trifluoromethyl)pyridin-2-yl)oxy)phenyl)-1*H*-1,2,3-triazol-1-yl)benzyl)morpholine (37a)**

To a stirring solution of **60** (0.050 g, 0.18 mmol, 1.0 equiv.) and **61** (0.047 g, 0.18 mmol, 1.0 equiv) in H_2_O/*t*-BuOH (1:1, 3 mL), a freshly prepared solution of sodium ascorbate (0.004 g, 0.018 mmol, 0.1 equiv.) in water was added, followed by CuSO_4_•5H_2_O (0.004 g, 0.00018 mmol, 0.01 equiv.) and the resulting solution was stirred at RT for 2 days. Then, NH_4_OH solution was added and extracted with EtOAc (3x10 mL). The combined organic layers were dried over Na_2_SO_4_, filtered, concentrated *in vacuo* to release 36a that was used directly in the next step (0.050 g, 54%). ^1^H NMR (500 MHz, Acetone-*d_6_*) *δ* 8.81 (s, 1H), 8.40 (d, *J*=5.3 Hz, 1H), 7.90 (dt, *J*=7.8, 1.2 Hz, 1H), 7.83 (dd, *J*=2.4, 1.6 Hz, 1H), 7.76 (d, *J*=2.1 Hz, 1H), 7.56 (t, *J*= 7.9 Hz, 1H), 7.48 (dd, *J*=8.5, 2.2 Hz, 1H), 7.43 (dd, *J*=5.3, 1.5 Hz, 1H), 7.38 (dt, *J*= 1.7, 0.8 Hz, 1H), 7.27 (d, *J* = 8.5 Hz, 1H), 7.20 (ddd, *J*=8.1, 2.4, 1.0 Hz, 1H), 3.94 (s, 3H), 3.61 (t, *J*= 4.7 Hz, 4H), 3.54 (s, 2H), 2.43 (t, *J*= 4.7 Hz, 4H).

**2-(4-Methoxy-3-nitrophenyl)-1,3-dioxolane (38)**

Into a dry flask charged with 4-methoxy-3-nitrobenzaldehyde (1.00 g, 5.52 mmol, 1.0 equiv) in dry toluene (28 mL, 0.2 M), ethylene glycol (0.685 g, 11.04 mmol, 2.0 equiv) was added. The mixture was stirred at reflux in a Dean-Stark apparatus for 24 h. Once the reaction was completed, the solvent was removed under reduced pressure and the crude mixture was used for the next step without any purification (**38**, 80 % as yellowish liquid). ^1^H NMR (500 MHz, DMSO-*d_6_*) *δ* 7.91 (d, *J*=2.1 Hz, 1H), 7.72 (dd, *J*=8.7, 2.1, 1H), 7.39 (d, *J*=8.7 Hz, 1H), 5.77 (s, 1H), 4.09 – 4.02 (m, 2H), 3.97 – 3.94 (m, 2H), 3.94 (s, 3H).

**5-(1,3-Dioxolan-2-yl)-2-methoxyaniline (39)**

To a stirring solution of **38** (0.513 g, 2.28 mmol, 1.0 equiv) dissolved in EtOAc (23 mL, 0.1 M), Pd/C 10 wt % (0.242 g, 0.227 mmol, 0.1 equiv) was added and the suspension was stirred for 2 h at RT under a hydrogen balloon. Once starting material was completed consumed, the crude material was passed through a bed of celite and washed several times with EtOAc. The organic phases were dried under reduced pressure to release **39** as a yellowish oil (0.376 g, 85%) ^1^H NMR (500 MHz, DMSO-*d_6_*) *δ* 7.16 (d, *J*=8.1 Hz, 1H), 7.10 (s, 1H), 6.99 (d, *J*=8.2 Hz, 1H), 5.77 (s, 1H), 4.09 – 4.02 (br, 2H), 3.97 – 3.94 (m, 2H), 5.11 (s, 2H), 3.87 (s, 3H).

**Methyl 3-((4-(trifluoromethyl)pyridin-2-yl)oxy)benzoate (40)**

To a stirring solution of methyl 3-hydroxybenzoate (0.500 g, 3.29 mmol, 1.0 equiv) and potassium *tert*-butoxide (0.406 g, 3.62 mmol, 1.1 equiv) in DMF (6.5 mL, 0.5 M), was added 2-chloro-4-(trifluoromethyl)pyridine (0.595 g, 3.29 mmol, 1.0 equiv) and the reaction mixture was heated to 95 °C overnight. Then, the mixture was diluted with H_2_O and washed with EtOAc several times. The combined organic layers were dried over Na_2_SO_4_, filtered, concentrated *in vacuo* and purified by flash chromatography (Cyclohexane/EtOAc, product at 10% of EtOAc) to release **40** as a colorless oil (0.350 g, 36%). ^1^H NMR (500 MHz, DMSO-*d_6_*) *δ* 8.40 (d, *J*=5.2, 1H), 7.89 – 7.82 (m, 1H), 7.73 – 7.69 (m, 1H), 7.61 (t, *J*=7.9 Hz, 1H), 7.55 (s, 1H), 7.54 – 7.50 (m, 2H), 3.86 (s, 3H).

**3-((4-(Trifluoromethyl)pyridin-2-yl)oxy)benzoic acid (41)**

Into a stirring solution of **40** (1.14 g, 3.83 mmol, 1.0 equiv) dissolved in MeOH (20 mL, 0.2 M), NaOH 10 % v/v (3 mL) was added and the solution was refluxed for 2 hours. Once the starting material was completed consumed, the solvent was removed, and the crude was washed with NH_4_Cl and extracted with EtOAc several times. The combined organic layers were dried over Na_2_SO_4_, filtered, concentrated *in vacuo* to afford **41** as a white powder (1.07 g, 99%). ^1^H NMR (500 MHz, DMSO-*d_6_*) *δ* 13.16 (br, 1H), 8.41 (d, *J*=5.2 Hz, 1H), 7.83 (d, *J*=7.7 Hz, 1H), 7.67 (s, 1H), 7.58 (t, *J*=7.9 Hz, 1H), 7.54 (s, 1H), 7.52 (d, *J*=5.3 Hz, 1H), 7.47 (d, *J*=8.0 Hz, 1H).

**3-((4-(Trifluoromethyl)pyridin-2-yl)oxy)benzoyl chloride (41a)**

Into a dry flask **41** (0.200 g, 0.706 mmol, 1.0 equiv) dissolved in DMF (3 mL) was refluxed for 3 h in presence of SOCl_2_ (3 mL). Then, the solvent was removed under reduced pressure to afford **41a** (0.213 g, quant.) that was used as crude directly in the next step.

***N*-(5-(1,3-Dioxolan-2-yl)-2-methoxyphenyl)-3-((4-(trifluoromethyl)pyridin-2-yl)oxy)benzamide (42)**

Into a dry flask, to a stirring solution of **41** (0.213 g, 0.706 mmol, 1.1 equiv) in dry EtOAc (3.5 mL, 0.2 M), 39 (0.125 g, 0.64 mmol, 1.0 equiv) and triethylamine (0.071 g, 0.706 mmol, 1.1 equiv) were added and the reaction was left stirring at RT on. Once the starting material was consumed, H_2_O was added to quench the reaction. The aqueous phase was extracted several times with EtOAc and the combined organic layers were dried over Na_2_SO_4_, filtered, concentrated *in vacuo* to afford **42** after flash chromatography (Cyclohexane/EtOAc, product at 10% of EtOAc) as a white solid (0.183 g, 62%). ^1^H NMR (500 MHz, DMSO-*d_6_*) *δ* 9.58 (s, 1H), 8.43 (d, *J*=5.2 Hz, 1H), 7.87 (d, *J*=7.8, 1H), 7.77 (d, *J*=1.9, 2H), 7.60 (t, *J*=7.9 Hz, 1H), 7.57 (s, 1H), 7.53 (d, *J*=5.2 Hz, 1H), 7.47 – 7.41 (m, 1H), 7.26 (dd, *J*=8.4, 2.1, 1H), 7.09 (d, *J*=8.5 Hz, 1H), 5.68 (s, 1H), 4.07 – 4.01 (m, 2H), 3.96 – 3.89 (m, 2H), 3.83 (s, 3H).

***N*-(5-formyl-2-methoxyphenyl)-3-((4-(trifluoromethyl)pyridin-2-yl)oxy)benzamide (43)**

To a stirred solution of 42 (0.115 g, 0.25 mmol, 1.0 equiv) in dioxane (5 mL) was added a solution of 10 % v/v HCl at RT. Then, the reaction mixture was stirred at 60 °C for 10 min. Then, the reaction was diluted with H_2_O and extracted several times with EtOAc. The combined organic layers were dried over Na_2_SO_4_, filtered, concentrated *in vacuo* to afford **43** as pure compound (0.100 g, quant).  ^1^H NMR (500 MHz, DMSO-*d_6_*) *δ* 9.90 (s, 1H), 9.74 (s, 1H), 8.43 (d, *J*=5.2 Hz, 1H), 8.26 (d, *J*=2.0 Hz, 1H), 7.88 (d, *J*=7.8 Hz, 1H), 7.81 (dd, *J*=8.5, 2.1 Hz , 1H), 7.80 – 7.78 (m, 1H), 7.62 (t, *J*=7.9 Hz, 1H), 7.57 (s, 1H), 7.54 (d, *J*=5.2 Hz, 1H), 7.49 – 7.44 (m, 1H), 7.32 (d, *J*=8.5 Hz, 1H), 3.94 (s, 3H).

**3-Fluoro-4-methoxy-5-nitrobenzaldehyde (44)**

To a stirring solution of H_2_SO_4_ conc. (10 mL), 3-fluoro-4-methoxybenzaldehyde (2.00 g, 12.97 mmol, 1.0 equiv) was slowly added at 0 °C. Then, HNO_3_ 70% w/w (1 mL) was added dropwise and the reaction was stirred at 0 °C for 2 h. Afterwards, the mixture was poured into ice and the formed precipitate was filtered off. The precipitate was partitioned between NaHCO_3_ ss and DCM (3 x 10 mL). The organic layers were combined, dried over Na_2_SO_4_, filtered, concentrated *in vacuo* and purified by column chromatography (Cyclohexane/Ethly Acetate, product at 20% of EtOAc) to afford **44** (1.2 g, 47%). ^1^H NMR (500 MHz, Acetone-*d_6_*) *δ* 10.02 (d, *J*= 2.0 Hz, 1H), 8.26 (t, *J*=1.7 Hz, 1H), 8.05 (dd, *J*=11.4, 2.0 Hz, 1H), 4.20 (d, *J*= 3.0 Hz, 3H). ^13^C NMR (126 MHz, Acetone-*d_6_*) *δ* 189.6, 156.4, 146.7, 145.3, 132.2, 122.6, 121.1, 63.3.

**4-(3-Fluoro-4-methoxy-5-nitrobenzyl)morpholine (45)**

Following GP4, **44** (0.400 g, 2.0 mmol, 1.0 equiv), morpholine (0.23 g, 2.6 mmol, 1.3 equiv), STAB (0.68 g, 3.2 mmol, 1.6 equiv) in DCE (2 mL) afforded after column chromatography (Cyclohexane/Ethly Acetate, product at 60% of EtOAc) **45** as a white solid (0.400 g, 74%). ^1^H NMR (500 MHz, DMSO-*d_6_*) *δ* = 7.69 (s, 1H), 7.62 (dd, *J*=12.0, 1.9 Hz, 1H), 3.96 (d, *J*=1.5 Hz, 3H), 3.61 – 3.55 (m, 4H), 3.50 (s, 2H), 2.39 – 2.34 (m, 4H). ^13^C NMR (126 MHz, DMSO-*d_6_*) *δ* 154.9, 143.7, 139.6, 135.2, 121.4, 119.9, 66.1, 62.6, 60.3, 52.9.

**3-Fluoro-2-methoxy-5-(morpholinomethyl)aniline (46)**

To a stirring solution of **45** (0.385 g, 1.42 mmol, 1.0 equiv) in EtOH/H_2_O (2:1, 0.1 M), Fe (0.396 g, 7.1 mmol, 5.0 equiv) and NH_4_Cl (0.076 g, 1.42 mmol, 1.0 equiv) were added at RT, and the mixture was stirred at reflux for 3 h. Once cooled, the reaction solution was passed through a bed of Celite® and washed with copious amounts of EtOH. The solvent was then removed under reduced pressure. The residue was taken up in EtOAc washed with NaHCO_3_ (3 x 10 mL), and the combined organic layers were dried over anhydrous Na_2_SO_4_, filtered and concentrated *in vacuo* to afford **46** as crude product (0.320 g, 94%). ^1^H NMR (500 MHz, DMSO-*d_6_*) *δ =* 6.44 (s, 1H), 6.28 (dd, *J*=11.8, 1.6 Hz, 1H), 5.18 (br, 2H), 3.69 (s, 3H), 3.53 – 3.58 (m, 4H), 3.24 (s, 2H), 2.32 – 2.28 (m, 4H).

**Chloro-4-methoxy-5-nitrobenzoic acid (47)**

To a fuming solution of HNO_3_ (12.5 mL), 3-chloro-4-methoxybenzoic acid (2.5 g, 13.4 mmol) was added portionwise at 0 °C and the mixture was stirred at RT for 2 h. Once the reaction was completed, cold H_2_O (25 mL) was added and the formed precipitate was filtered off. The solid product was redissolved in DCM, washed with brine and dried under reduced vacuum to release **47** as a white powder (1.85 g, 60%). ^1^H NMR (500 MHz, DMSO-*d_6_*) *δ* 8.35 (d, *J*=2.1 Hz, 1H), 8.26 (d, *J*=2.1 Hz, 1H), 3.99 (s, 3H).

**(3-Chloro-4-methoxy-5-nitrophenyl)methanol (48)**

A solution of borane dimethyl sulfide complex solution 2.0M in THF (8.3 mL, 16.6 mmol, 2.1 equiv.) was added dropwise to a solution of **47** (1.85 g, 7.9 mmol, 1.0 equiv.) in THF (26.3 mL, 0.3 M) at 0° C and the mixture was stirred at RT for 48 hours. The reaction was quenched by dropwise addition of MeOH at 0°C. The crude mixture was purified by flash chromatography (product at 20% EtOAc on Cyclohexane) to afford **48** as a slight yellow solid (1.20 g, 70%). ^1^H NMR (500 MHz, DMSO-*d_6_*) *δ* 7.87 – 7.83 (m, 1H), 7.81 – 7.76 (m, 1H), 5.57 (t, *J*=5.0, 1H), 4.53 (d, *J*=4.3 Hz, 2H), 3.91 (s, 3H).

**3-Chloro-4-methoxy-5-nitrobenzaldehyde (49)**

To a solution of **48** (0.940 g, 4.35 mmol, 1.0 equiv.) in DCM (22 mL, 0.2 M), pyridinium chlorochromate (1.87 g, 8.7 mmol, 2.0 equiv) was added and, the reaction was stirred at RT for 6 hours. Once the reaction was completed, it was passed through a bed of celite and washed several times with DCM. The collected solution was dried and purified by flash chromatography (product at 20% EtOAc on Cyclohexane) to release **49** as a slight yellow powder (0.682 g, 73%). ^1^H NMR (500 MHz, DMSO-*d_6_*) *δ* 9.98 (s, 1H), 8.45 (d, *J*=2.0 Hz, 1H), 8.37 (d, *J*=2.0 Hz, 1H), 4.02 (s, 3H). ^13^C NMR (126 MHz, DMSO-*d_6_*) *δ* 189.9, 152.9, 145.1, 134.9, 133.1, 130.0, 124.8, 62.9.

**4-(3-Chloro-4-methoxy-5-nitrobenzyl)morpholine (50)**

Following GP4, **49** (0.678 g, 3.14 mmol), morpholine (0.356 g, 4.08 mmol) and STAB (1.06 g, 5.024 mmol) in DCE (15 mL) afforded after column chromatography (Cyclohexane/EtOAc, product at 40% of EtOAc) **50** (0.660 g, 73%) as white powder. ^1^H NMR (500 MHz, DMSO-*d_6_*) *δ* 7.86 – 7.82 (m, 1H), 7.82 – 7.76 (m, 1H), 3.92 (s, 3H), 3.61 – 3.54 (m, 4H), 3.51 (s, 2H), 2.34 – 2.40 (m, 4H).

**3-Chloro-2-methoxy-5-(morpholinomethyl)aniline (51)**

To a stirring solution of **50** (0.660 g, 2.3 mmol, 1.0 equiv) in EtOH/H_2_O (2:1, 0.1 M), Fe (0.642 g, 11.5 mmol, 5.0 equiv) and NH_4_Cl (0.123 g, 2.3 mmol, 1.0 equiv) were added at RT, and the mixture was stirred at reflux for 3 h. Once cooled, the reaction solution was passed through a bed of Celite® and washed with copious amounts of EtOH. The solvent was then removed under reduced pressure. The residue was taken up in EtOAc washed with NaHCO_3_ (3 x 10 mL), and the combined organic layers were dried over anhydrous Na_2_SO_4_, filtered and concentrated *in vacuo* to afford **51** as crude product (0.320 g, 94%). ^1^H NMR (500 MHz, DMSO-*d_6_*) *δ* 6.60 (d, *J*=1.9 Hz, 1H), 6.48 (d, *J*=1.9 Hz, 1H), 5.21 (s, 2H), 3.65 (s, 3H), 3.62 – 3.49 (m, 4H), 3.24 (s, 2H), 2.31 (s, 4H). ^13^C NMR (126 MHz, DMSO-*d_6_*) *δ* 142.8, 140.5, 134.9, 126.1, 116.4, 114.1, 66.2, 61.9, 59.0, 53.1.

**4-(4-Fluoro-3-nitrobenzyl)morpholine (52)**

4-(Chloromethyl)-1-fluoro-2-nitrobenzene (1.00 g, 5.27 mmol, 1.0 equiv) was added to a stirring solution of morpholine (0.596 g, 6.85 mmol, 1.3 equiv) and TEA (0.800 g, 7.9 mmol, 1.5 equiv) in DCM (26 mL, 0.2M), and the reaction mixture was stirred at RT on. Once starting material was consumed, the mixture was washed with water and extracted with DCM (3 x 10 mL). The organic layers were combined, dried over Na_2_SO_4_, filtered, concentrated *in vacuo* and purified by flash prep to afford **52** (0.200 g, 16%). ^1^H NMR (500 MHz, Acetone-*d_6_*) *δ* 8.16 – 8.04 (m, 1H), 7.79 (t, *J*=6.8 Hz, 1H), 7.45 (dd, *J*=11.2, 8.5 Hz, 1H), 3.63 (t, *J*=4.5 Hz, 4H), 3.60 (s, 2H), 2.44 (t, *J*=4.6 Hz, 4H).

**2-Fluoro-5-(morpholinomethyl)aniline (53)**

To a stirring solution of **52** (0.180 g, 0.75 mmol, 1.0 equiv) in EtOH/H_2_O (2:1, 0.1 M), Fe (0.209 g, 3.74 mmol, 5.0 equiv) and NH_4_Cl (0.020 g, 0.37 mmol, 0.5 equiv) were added at RT, and the mixture was stirred at reflux for 1 h. Once cooled, the reaction solution was passed through a bed of Celite® and washed with copious amounts of EtOH. The solvent was then removed under reduced pressure. The residue was taken up in EtOAc washed with NaHCO_3_ (3 x 10 mL), and the combined organic layers were dried over anhydrous Na_2_SO_4_, filtered and concentrated *in vacuo.* The crude material (**53**) was used directly in the next step without any purification (0.115 g, 73%).

**4-(Chloromethyl)pyridin-2-amine (54)**

To a stirring solution of (2-aminopyridin-4-yl)methanol (1.0 g, 8.05 mmol, 1.0 equiv.), SOCl_2_ (2 mL) was slowly added at 0°C and the solution was stirred at 60 °C for 2 hours. Then, solvent was removed under reduced pressure with an azeotropic distillation with toluene. The crude material (**54**) was used directly in the next step without any purification (1.10 g, 99%).

**4-(Morpholinomethyl)pyridin-2-amine (55)**

To a stirring solution of morpholine (0.317 g, 3.64 mmol, 1.3 equiv) and TEA (0.340 g, 3.36 mmol, 1.2 equiv) in DMF (23 mL, 0.3 M), 54 (0.400 g, 2.8 mmol, 1.0 equiv) was added and the reaction was stirred at RT for 2 days. Then, the reaction was diluted with NH_4_Cl and extracted with DCM (3x10 mL). The combined organic layers were dried over Na_2_SO_4_, filtered, concentrated *in vacuo* and the crude product was purified by flash chromatography (DCM/MeOH) to afford **55** (0.300 g, 55%) ^1^H NMR (500 MHz, *Acetone*) *δ =* 7.82 (d, *J*=5.4 Hz, 1H), 6.64 (s, 1H), 6.60 (d, *J*=5.2 Hz, 1H), 3.67 – 3.57 (m, 4H), 3.38 (s, 2H), 2.42 - 2.42 (s, 4H).

**4-(4-Methoxy-3-nitrobenzyl)morpholine (56)**

Following GP4, 4-methoxy-3-nitrobenzaldehyde (0.300 g, 1.65 mmol, 1.0 equiv), morpholine (0.187 g, 2.14 mmol, 1.3 equiv), STAB (0.56 g, 2.64 mmol, 1.6 equiv) in DCE (8 mL) afforded after column chromatography (Cyclohexane/Ethly Acetate, product at 60% of EtOAc) **56** as a yellowish powder (0.360 g, 86%). ^1^H NMR (500 MHz, DMSO-*d_6_*) *δ* = 7.78 (d, *J*=2.1 Hz, 1H), 7.59 (dd, *J*=8.6, 2.2 Hz, 1H), 7.32 (d, *J*=8.6 Hz, 1H), 3.90 (s, 3H), 3.61 – 3.53 (m, 4H), 3.46 (s, 2H), 2.37 – 2.31 (m, 4H).

**2-Methoxy-5-(morpholinomethyl)aniline (57)**

To a stirring solution of **56** (0.342 g, 1.35 mmol, 1.0 equiv) dissolved in EtOAc (13 mL, 0.1 M), Pd/C 10 wt % (0.143 g, 0.135 mmol, 0.1 equiv) was added and the suspension was stirred for 2 h at RT under a hydrogen balloon. Once starting material was completed consumed, the crude material was passed through a bed of celite and washed several times with EtOAc. The organic phases were dried under reduced pressure to release **57** as a white powder (0.290 g, 97%). ^1^H NMR (500 MHz, DMSO-*d_6_*) *δ=* 6.69 (t, *J*=7.5 Hz, 1H), 6.60 (d, *J*=5.1 Hz, 1H), 6.43 (d, *J*=5.4 Hz, 1H), 4.62 (br, 2H), 3.72 (t, *J*=5.4 Hz, 3H), 3.56 – 3.48 – 3.56 (m, 4H), 3.24 (s, 2H), 2.33 – 2.25 (m, 4H).

**(3-((4-(Trifluoromethyl)pyridin-2-yl)oxy)phenyl)methanol (58)**

A solution of borane dimethyl sulfide complex solution 2.0 M in THF (0.9 mL, 1.78 mmol, 2.1 equiv.) was added dropwise to a solution of **41** (0.240 g, 0.85 mmol, 1.0 equiv.) in THF (3 mL, 0.3 M) at 0° C and the mixture was stirred at RT for 48 hours. The reaction was quenched by dropwise addition of MeOH at 0°C. The crude mixture was purified by flash chromatography (product at 50% EtOAc on Cyclohexane) to afford **58** as a colourless oil (1.33 g, 59 %). ^1^H NMR (500 MHz, DMSO-*d_6_*) *δ* = 8.40 (d, *J*=5.2 Hz, 1H), 7.49 (d, *J*=5.2 Hz, 1H), 7.46 (s, 1H), 7.39 (t, *J*=7.8 Hz, 1H), 7.20 (d, *J*=8.0 Hz, 1H), 7.11 (s, 1H), 7.04 (dd, *J*=7.9, 1.8 Hz, 1H), 5.29 (s, 1H), 4.51 (d, *J*=2.7 Hz, 2H).

**3-((4-(Trifluoromethyl)pyridin-2-yl)oxy)benzaldehyde (59)**

To a solution of **58** (0.120 g, 0.45 mmol, 1.0 equiv.) in DCM (3 mL, 0.2 M), pyridinium chlorochromate (0.192 g, 0.89 mmol, 2.0 equiv) was added and, the reaction was stirred at RT for 3 hours. Once the reaction was completed, it was passed through a bed of celite and washed several times with DCM. The collected solution was dried and purified by flash chromatography (product at 25% EtOAc on Cyclohexane) to release **59** as a colourless oil (0.682 g, 73%). ^1^H NMR (500 MHz, DMSO-*d_6_*) *δ* 10.02 (s, 1H), 8.68 (d, *J*=4.0 Hz, 1H), 8.41 (d, *J*=4.8 Hz, 1H), 7.83 (d, *J*=7.4 Hz, 1H), 7.73 – 7.66 (m, 2H), 7.58 (s, 2H).

**2-(3-Ethynylphenoxy)-4-(trifluoromethyl)pyridine (60)**

To a stirring solution of 3-ethynylphenol (0.100 g, 0.84 mmol, 1.0 equiv) and potassium carbonate (0.170 g, 0.93 mmol, 1.1 equiv) in DMF (6.5 mL, 0.5 M), was added 2-chloro-4-(trifluoromethyl)pyridine (0.129 g, 0.93 mmol, 1.1 equiv) and the reaction mixture was heated to 90 °C for 2 days. Then, the mixture was diluted with H_2_O and washed with EtOAc several times. The combined organic layers were dried over Na_2_SO_4_, filtered, concentrated *in vacuo* and purified by flash chromatography (Cyclohexane/EtOAc, product at 10% of EtOAc) to release **60** (0.075 g, 34%). ^1^H NMR (500 MHz, Acetone-*d_6_*) *δ* 8.38 (d, *J*= 5.2 Hz, 1H), 7.50 – 7.42 (m, 2H), 7.41 – 7.35 (m, 2H), 7.34 (t, *J*= 2.0 Hz, 1H), 7.27 (dd, *J*= 8.3, 2.3 Hz, 1H), 3.75 (s, 1H).

**4-(3-Azido-4-methoxybenzyl)morpholine (61)**

To a cooled solution of **57** (0.200 g, 0.89 mmol, 1.0 equiv.) in ACN (4.45 mL, 0.2M), *t*-BuNO_2_ (0.370 g, 3.59 mmol, 4.0 equiv.) and Me_3_SiN_3_ (0.311 g, 2.69 mmol, 3.0 equiv) were added dropwise. The reaction was stirred at RT on. Then, H_2_O was added, and the solution was diluted with DCM (3 x 10 mL). The combined organic layers were dried over Na_2_SO_4_, filtered, concentrated *in vacuo* to release **61** that was used directly in the next step (0.05 g, 25%).

## Chemicals, Materials and Methods

NMR experiments were run on a Bruker Ultrashield plus 500 (500 MHz) spectrometer. Spectra were acquired at 300 K, using deuterated dimethylsulfoxide (DMSO-*d*_6_) as solvent. Chemical shifts for ^1^H and ^13^C spectra were recorded in parts per million (ppm) using the residual non-deuterated solvent as the internal standard (for DMSO-*d*_6_: 2.50 ppm, ^1^H; 39.52 ppm, ^13^C; for CDCl_3_: 7.27 ppm, ^1^H; 77.00, ^13^C; for methanol-*d*_4_: 4.87 ppm, ^1^H; 49.15 ppm, ^13^C; for acetone-*d*_6_, 2.05 ppm, ^1^H; 29.32 ppm, ^13^C). Coupling constants (*J*) are given in Hertz (Hz). Data are reported as follows: chemical shift, multiplicity (s = singlet, d =doublet, t = triplet, m = multiplet, br = broad and combinations of these) coupling constants and integration. Flash chromatography was performed using the automated flash chromatography system CombiFlash Rf+ or NextGen 300+ (Teledyne Isco, Lincoln, NE, USA) equipped with RediSepRf silica columns (Axel S.D.rau, Sprockhövel Germany). TLC was performed with aluminium-backed silica TLC plates (Macherey-Nagel MN ALUGRAM Sheets SIL G/UV 254 20 x 20cm 818133) with a suitable solvent system and was visualized using UV fluorescence (254 & 366 nm). All reactions were carried out in oven-dried glassware under an atmosphere of argon. Anhydrous DMF was purchased from Aldrich and used directly.

Liquid chromatography-mass spectrometry was performed on a LC-MS system, consisting of a Dionex UltiMate 3000 pump, autosampler, column compartment and MWD or DAD detector (Thermo Fisher Scientific, Dreieich, Germany) and ESI quadrupole MS (MSQ Plus or ISQ EC, Thermo Fisher Scientific, Dreieich, Germany). Columns used: 1) Hypersil Gold column, 100 x 2.1 mm, 3 µm. At a flow rate of 700 µL/min, the gradient of H_2_O (0.1% FA) and ACN (0.1% FA) starting from 5% ACN and then increased to 100% over 7 min. 2) Hypersil Gold column, 100 x 1.9 mm, 2.1 µm. At a flow rate of 600 µL/min, the gradient of H_2_O (0.1% FA) and ACN (0.1% FA) starting from 5% ACN and then increased to 100% over 5.5 min. The mass spectrum was measured in positive and negative mode in a range from 100–600 m/z. The UV spectrum was recorded at 254 nm. High-resolution mass spectra (HR-MS) were recorded with a ThermoScientific system where a Dionex Ultimate 3000 RSLC was coupled to a Q Exactive Focus mass spectrometer with an electrospray ion (ESI) source. An Acquity UPLC® BEH C8, 150 x 2.1 mm, 1.7 µm column equipped with a VanGuard Pre-Column BEH C8, 5 x 2.1 mm, 1.7 µm (Waters, Germany) was used for separation. At a flow rate of 250 µL/min, the gradient of (A) H_2_O + 0.1% FA and (B) ACN + 0.1% FA was held at 10% B for 1 min and then increased to 95% B over 4 min. It was held there for 1.2 min before the gradient was decreased to 10% B over 0.3 min where it was held for 1 min. The mass spectrum was measured in positive mode in a range from 120–1000 m/z. UV spectrum was recorded at 254 nm.

Preparative RP-HPLC was performed using an UltiMate 3000 S.D.i-Preparative System (Thermo Fisher Scientific) with nucleodur® C18 Gravity (250 mm × 16 mm, 5 μm). Separation was done using gradient 5–100% CH_3_CN + 0.05% HCOOH in water + 0.05% HCOOH in 53 min at a flow rate of 10 mL/min and end with a 5 min step at 100% CH_3_CN. The sample was dissolved in DMSO and manually injected to the HPLC system.

## Abbreviations

Acetonitrile (ACN), Azidotrimethylsilane (Me_3_SiN_3_), BMS (Borane dimethyl sulfide complex solution), copper(II) sulfate pentahydrate (CuSO_4_•5H_2_O), dichloromethane (DCM), *N*-diisopropylethylamine (DIPEA), dimethylformamide (DMF), dimethylsulfoxide (DMSO), *N*-ethyl-*N*′-(3-dimethylaminopropyl)carbodiimide hydrochloride (EDC), ethyl acetate (EtOAc), formic acid (FA), flash column chromatography (FCC), high-performance liquid chromatography (HPLC), Hydroxybenzotriazole (HOBt), liquid chromatography–mass spectrometry (LCMS), *tert*-Butanol (*t*-BuOH), *tert*-Butyl nitrite (*t*-BuNO_2_), trifluoroacetic acid (TFA), tetrahydrofuran (THF), Thionyl chloride (SOCl_2_), triethylamine (TEA), thin-layer chromatography (TLC), tetramethylsilane (TMS). Other abbreviations used are: aqueous (aq.), calculated (calcd.), hours (h), minutes (min), room temperature (RT), overnight (on), saturated (sat.).

## NMR spectra

**Figure S6: ^1^H-NMR, ^13^C-NMR, HRMS analysis of compound 1**

**Figure S7: ^1^H-NMR, ^13^C-NMR, HRMS analysis of compound 2**

**Figure S8: ^1^H-NMR, ^13^C-NMR, HRMS analysis of compound 3**

**Figure S9: ^1^H-NMR, ^13^C-NMR, HRMS analysis of compound 4**

**Figure S10: ^1^H-NMR, ^13^C-NMR, HRMS analysis of compound 5**

**Figure S11: ^1^H-NMR, ^13^C-NMR, HRMS analysis of compound 6**

**Figure S12: ^1^H-NMR, ^13^C-NMR, HRMS analysis of compound 7**

**Figure S13: ^1^H-NMR, ^13^C-NMR, HRMS analysis of compound 8**

**Figure S14: ^1^H-NMR, ^13^C-NMR, HRMS analysis of compound 9**

**Figure S15: ^1^H-NMR, ^13^C-NMR, HRMS analysis of compound 10**

**
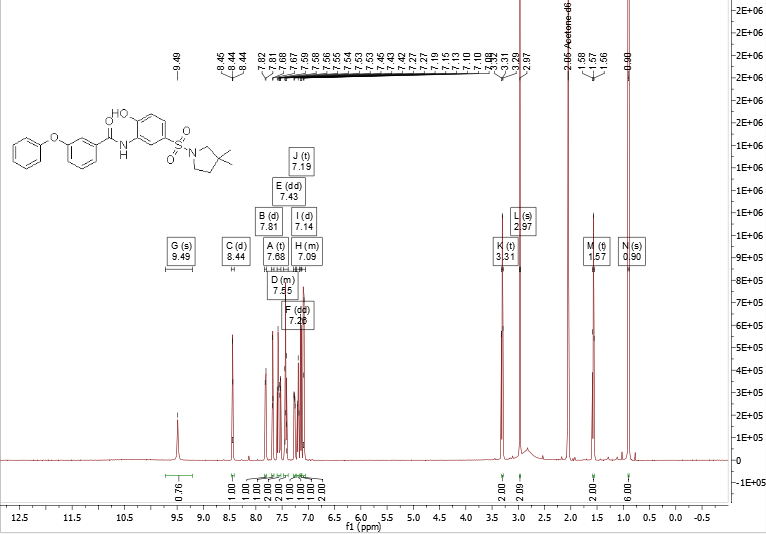
**

**
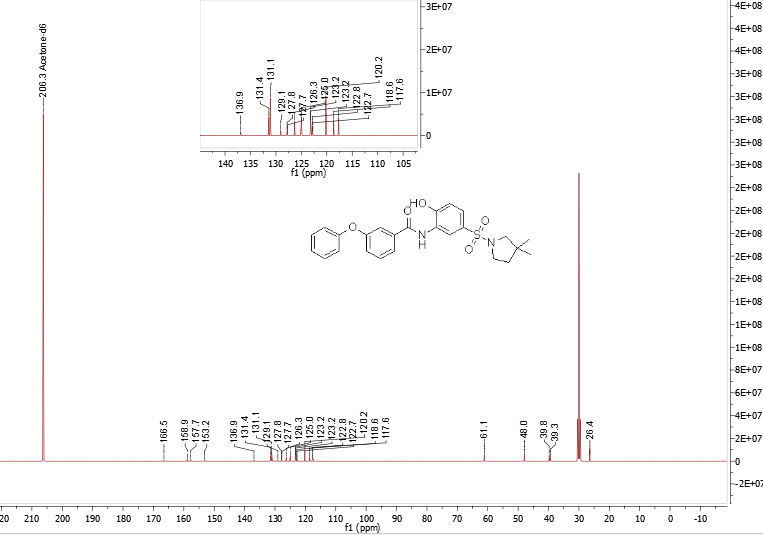
**

**Figure S16: ^1^H-NMR, ^13^C-NMR, HRMS analysis of compound 11**

**Figure S17: ^1^H-NMR, ^13^C-NMR, HRMS analysis of compound 12**

**Figure S18: ^1^H-NMR, ^13^C-NMR, HRMS analysis of compound 13**

**Figure S19: ^1^H-NMR, ^13^C-NMR, HRMS analysis of compound 14**

**
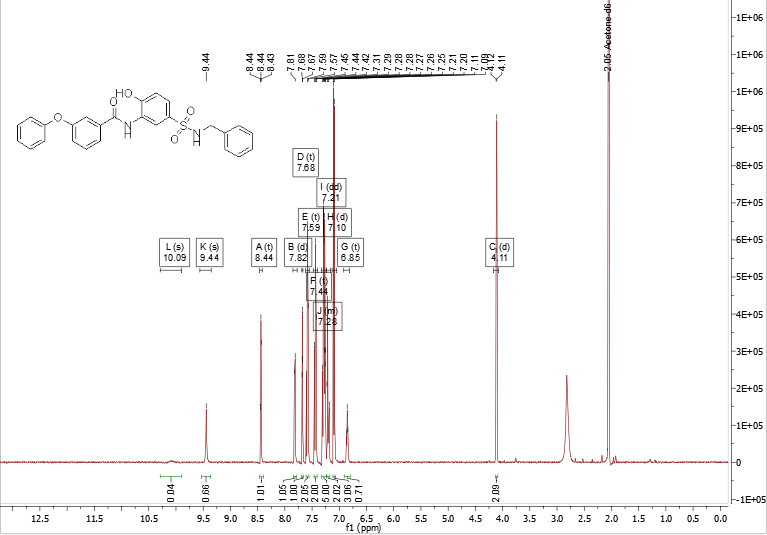
**

**
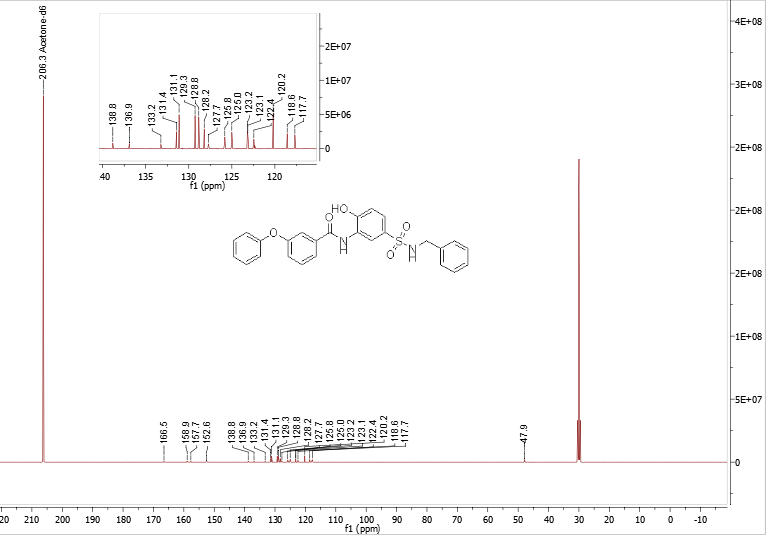
**

**Figure S20: ^1^H-NMR, ^13^C-NMR, HRMS analysis of compound 15**


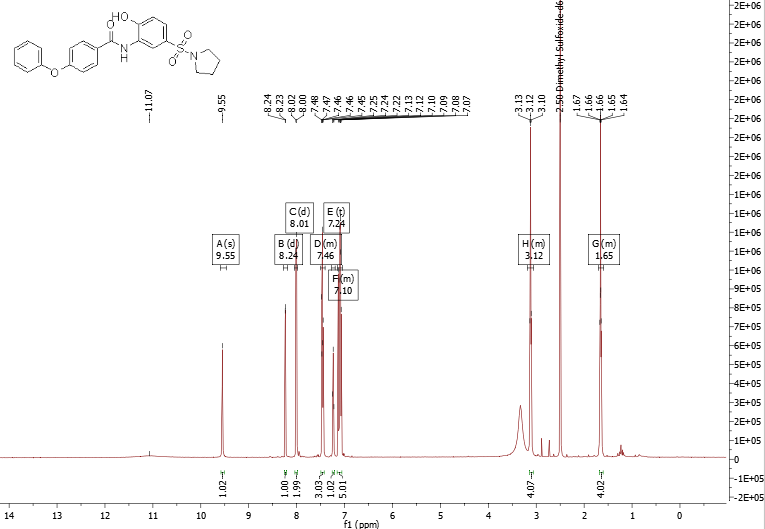


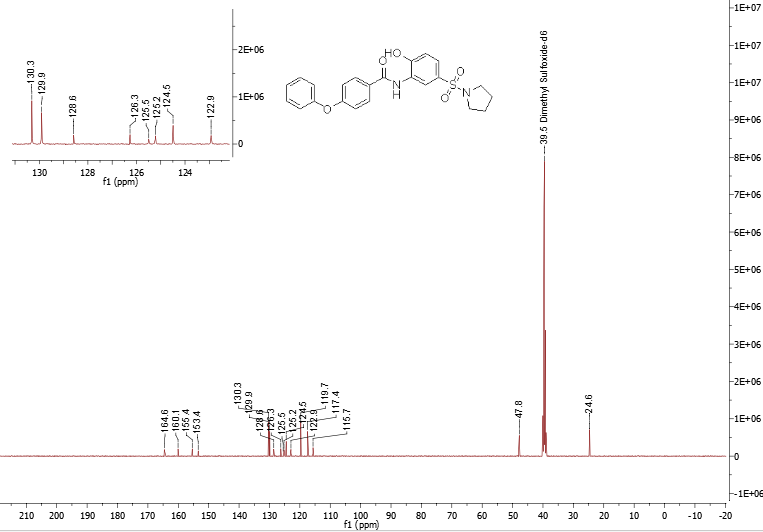


**Figure S21: ^1^H-NMR, ^13^C-NMR, HRMS analysis of compound 16**


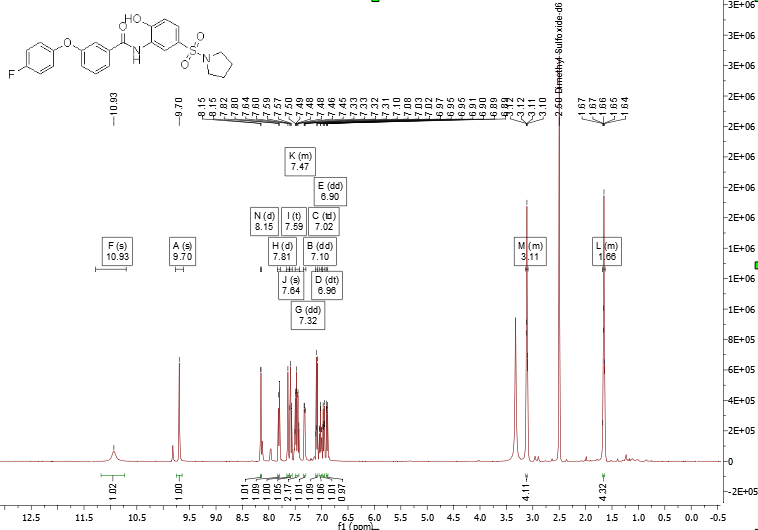


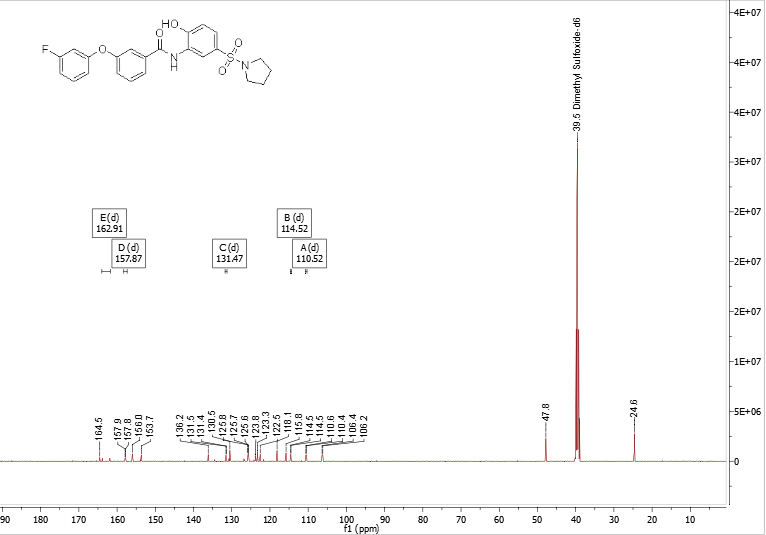


**Figure S22: ^1^H-NMR, ^13^C-NMR, HRMS analysis of compound 17**


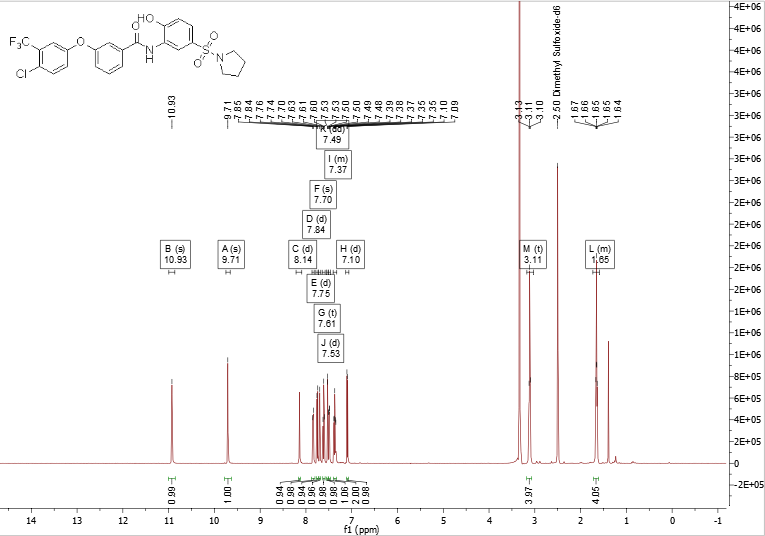


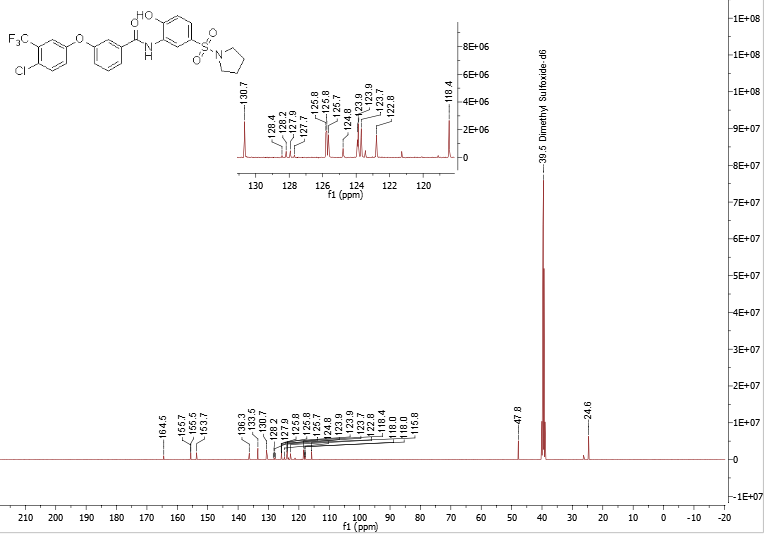


**Figure S23: ^1^H-NMR, ^13^C-NMR, HRMS analysis of compound 18**


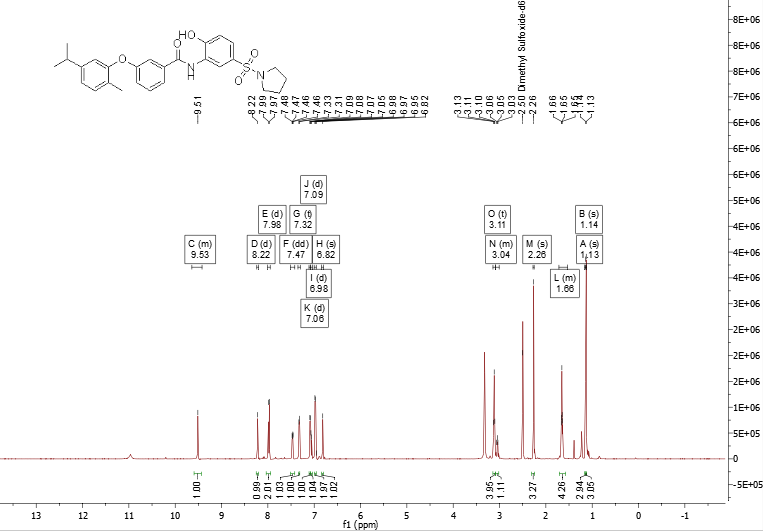


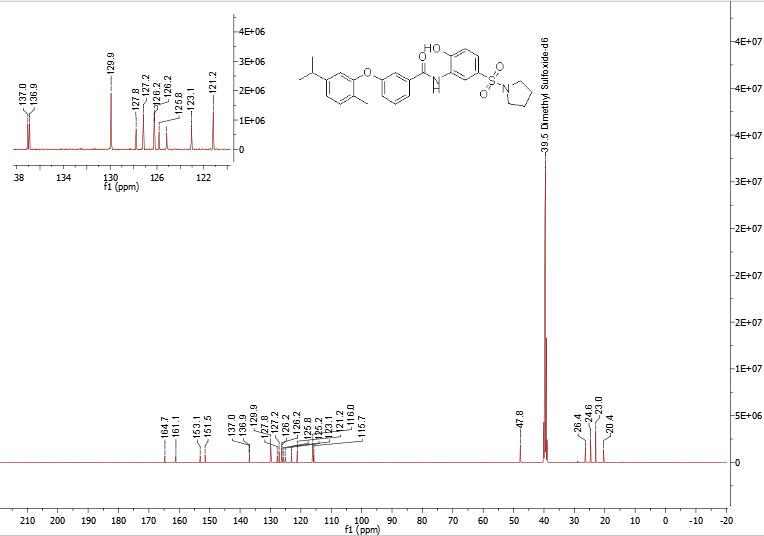


**Figure S24: ^1^H-NMR, ^13^C-NMR, HRMS analysis of compound 19**


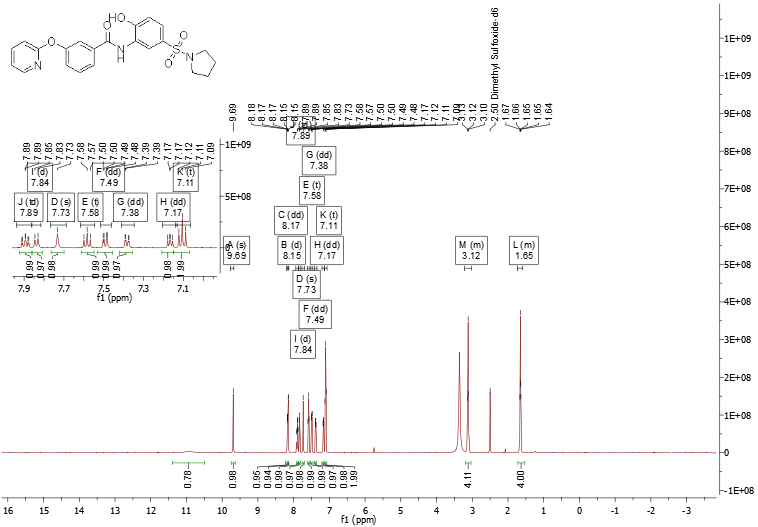


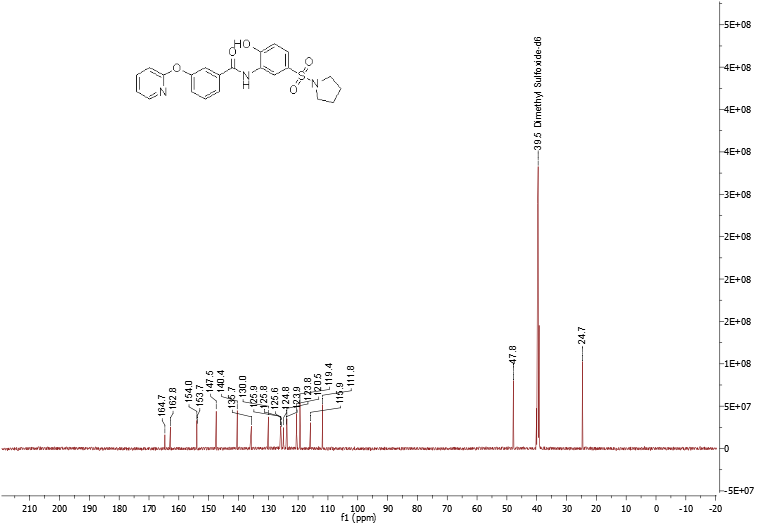


**Figure S25: ^1^H-NMR, ^13^C-NMR, HRMS analysis of compound 20**


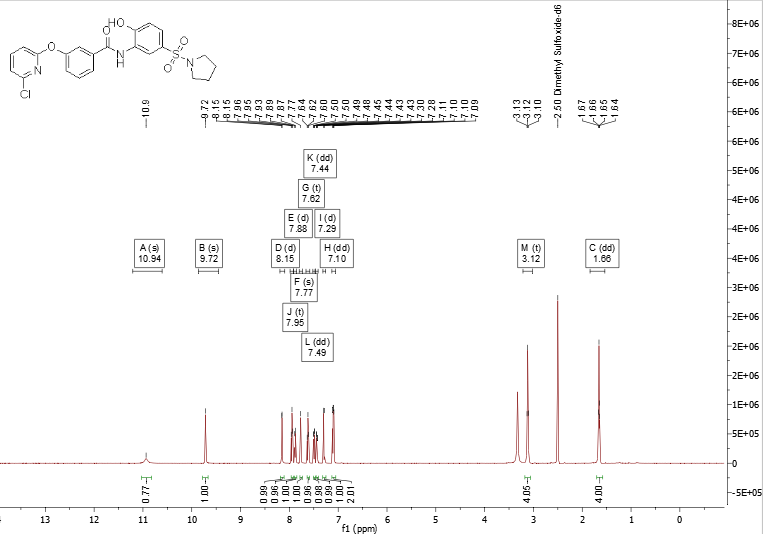


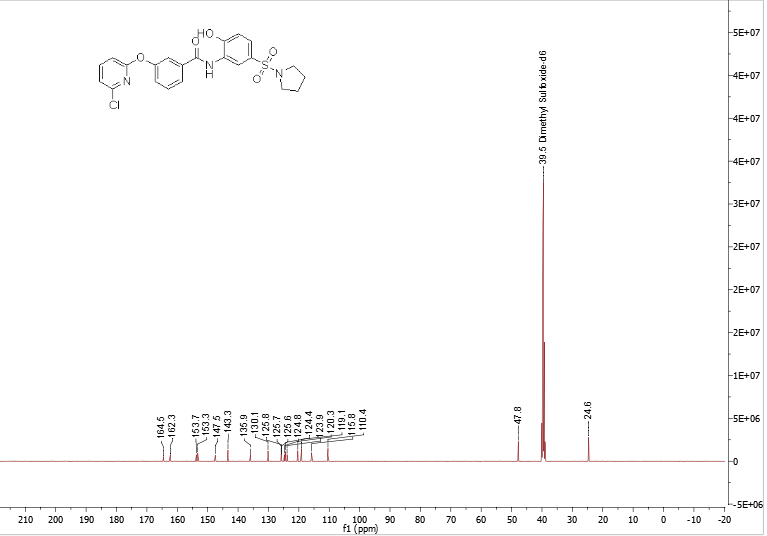


**Figure S26: ^1^H-NMR, ^13^C-NMR, HRMS analysis of compound 21**


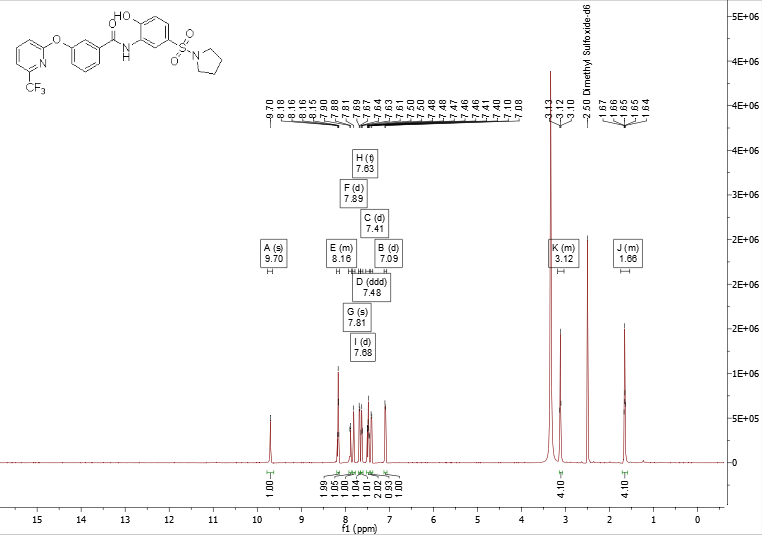


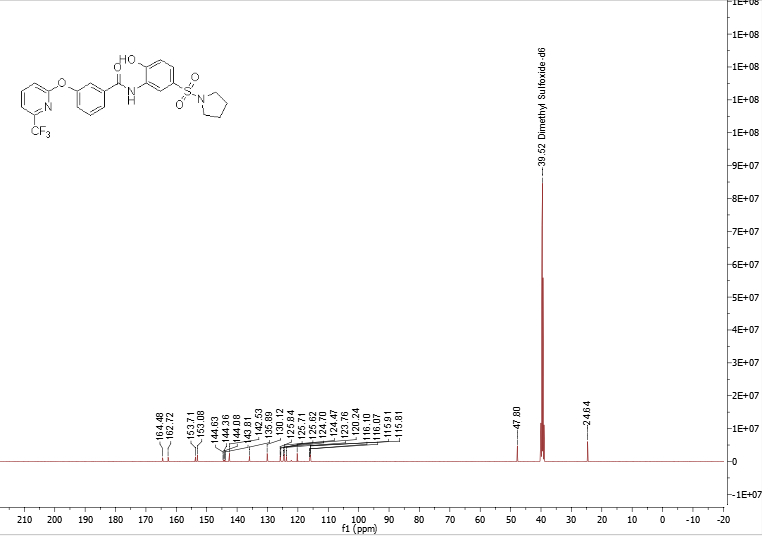


**Figure S27: ^1^H-NMR, ^13^C-NMR, HRMS analysis of compound 22**


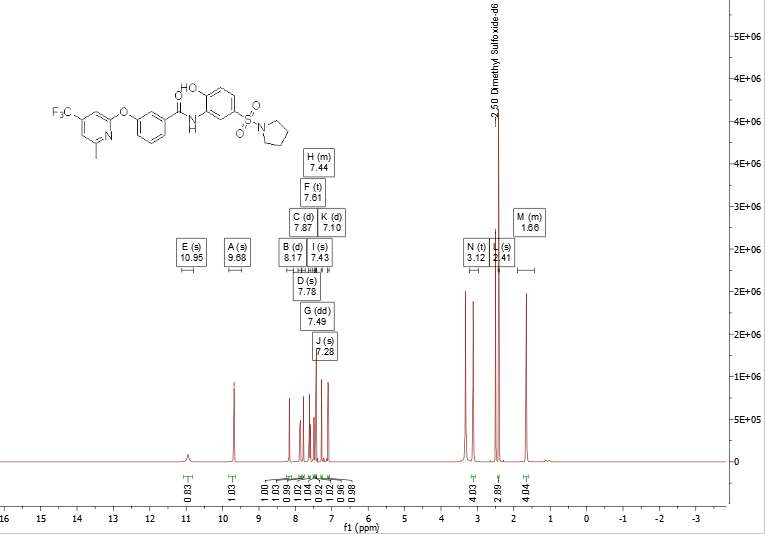


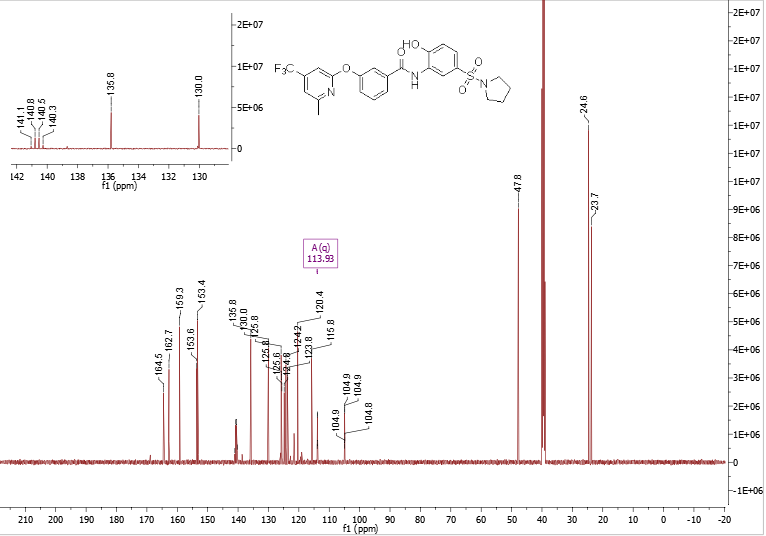


**Figure S28: ^1^H-NMR, ^13^C-NMR, HRMS analysis of compound 23**

**
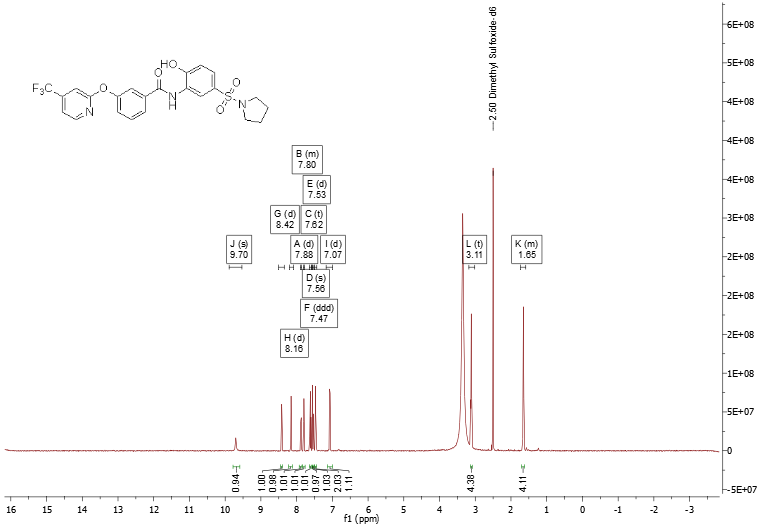
**

**
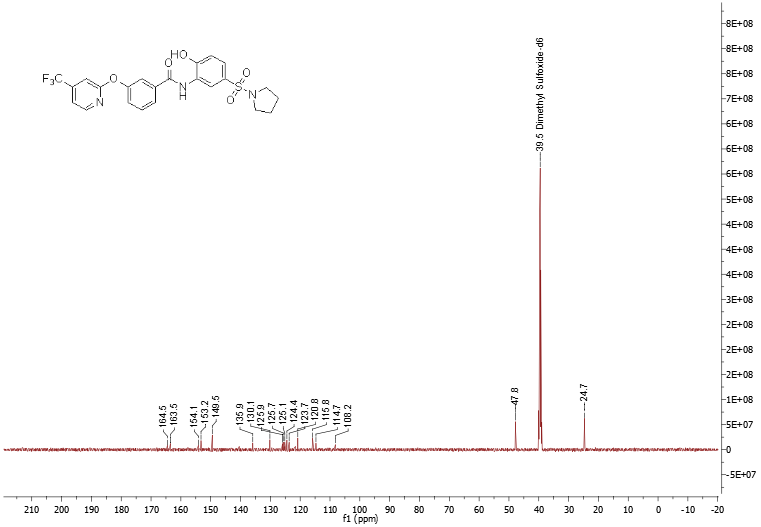
**

**
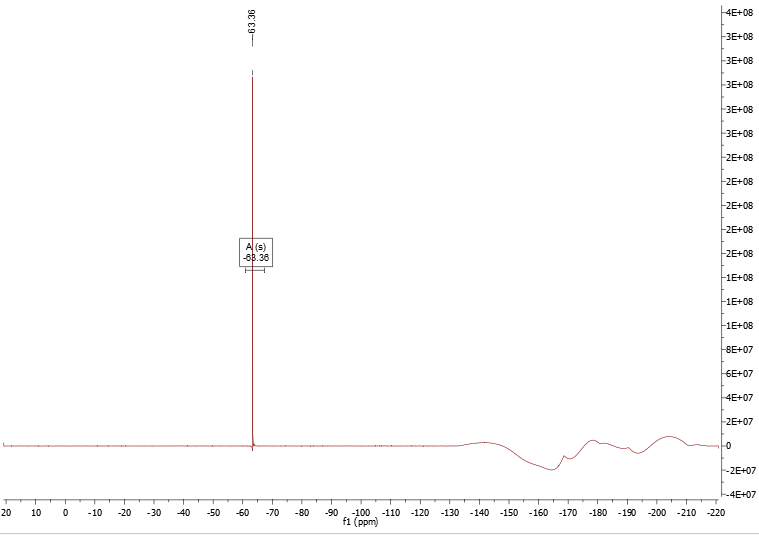
**

**Figure S29: ^1^H-NMR, ^13^C-NMR, HRMS analysis of compound 24**


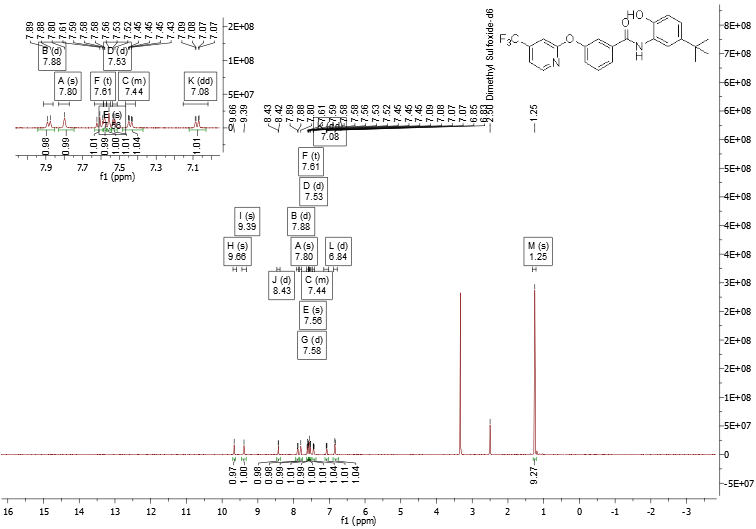


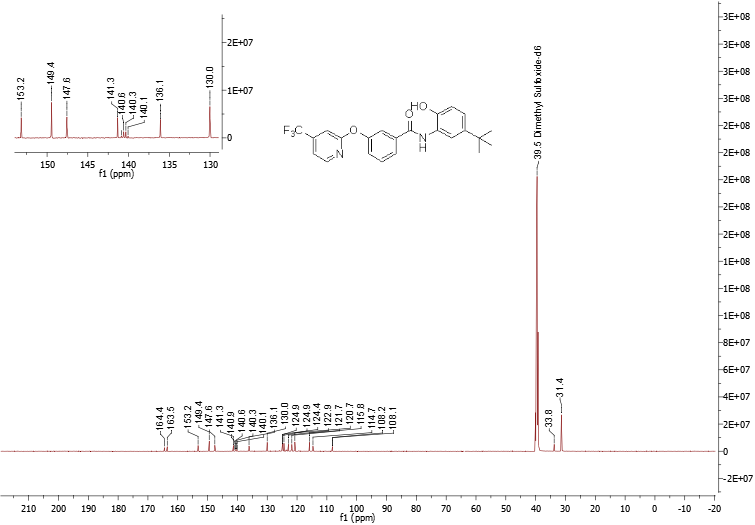


**Figure S30: ^1^H-NMR, ^13^C-NMR, HRMS analysis of compound 25**

**
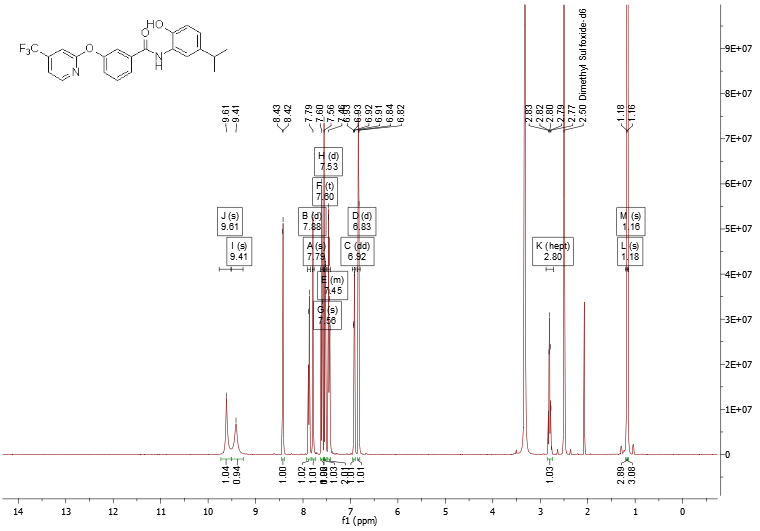
**

**
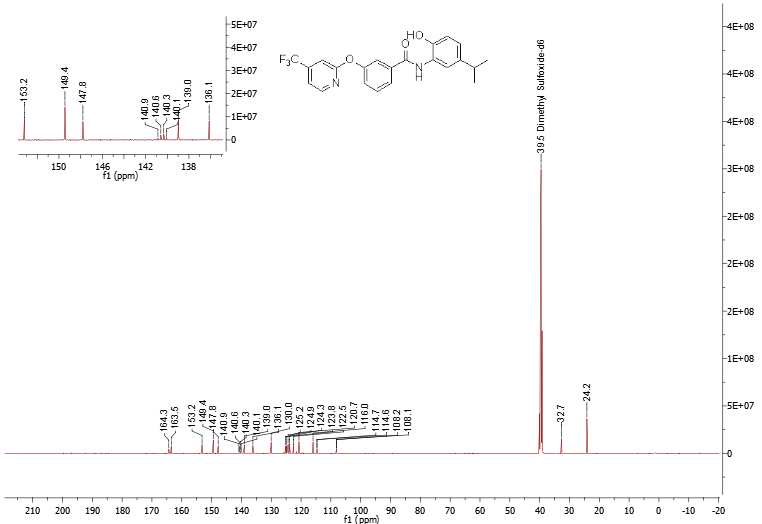
**

**Figure S31: ^1^H-NMR, ^13^C-NMR, HRMS analysis of compound 26**

**
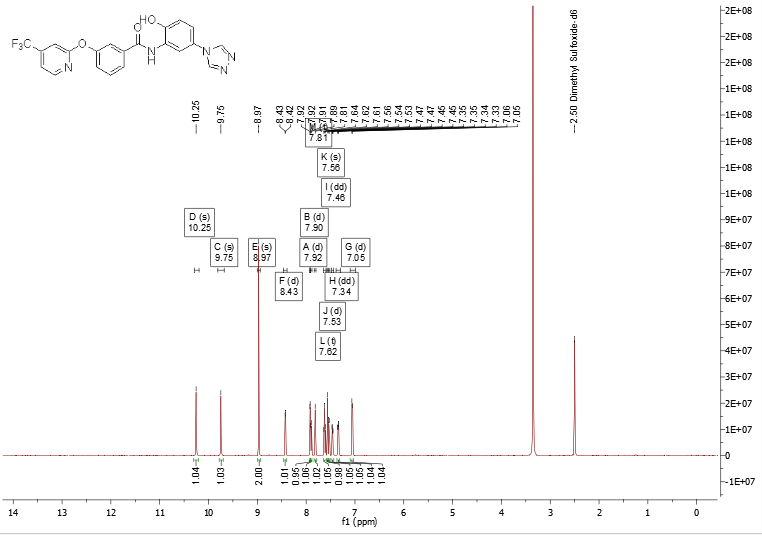
**

**
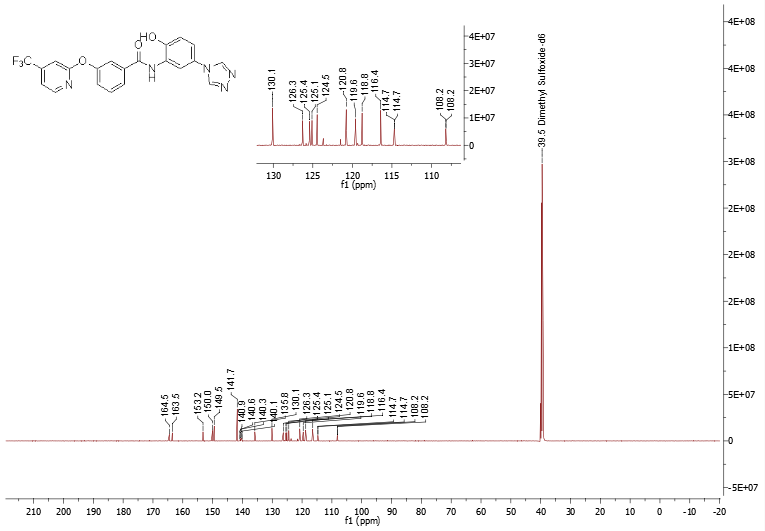
**

**Figure S32: ^1^H-NMR, ^13^C-NMR, HRMS analysis of compound 27**

**
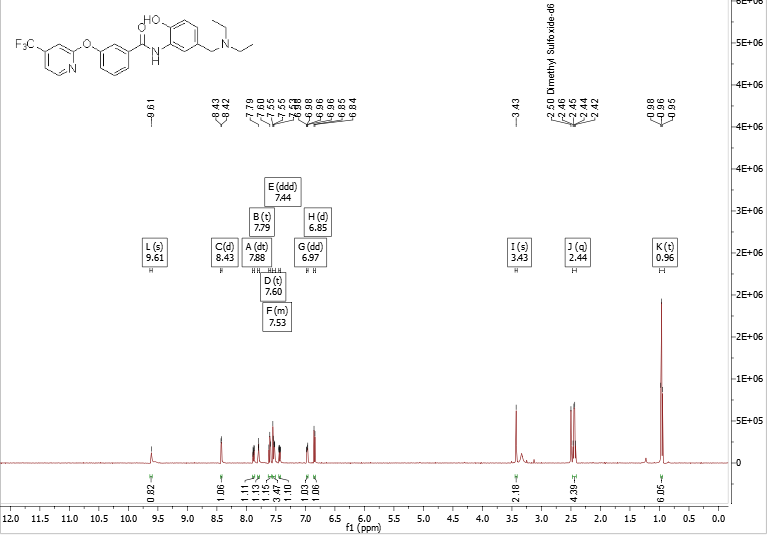
**

**
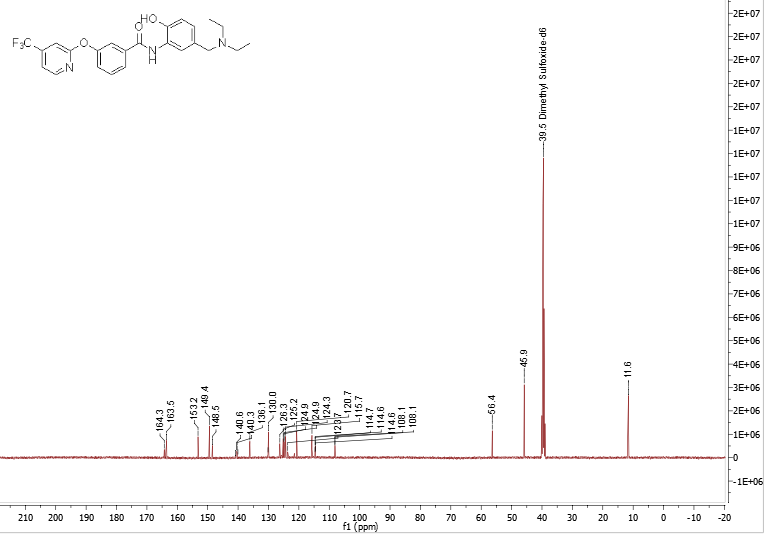
**

**Figure S33: 1H-NMR, 13C-NMR, HRMS analysis of compound 28**

**
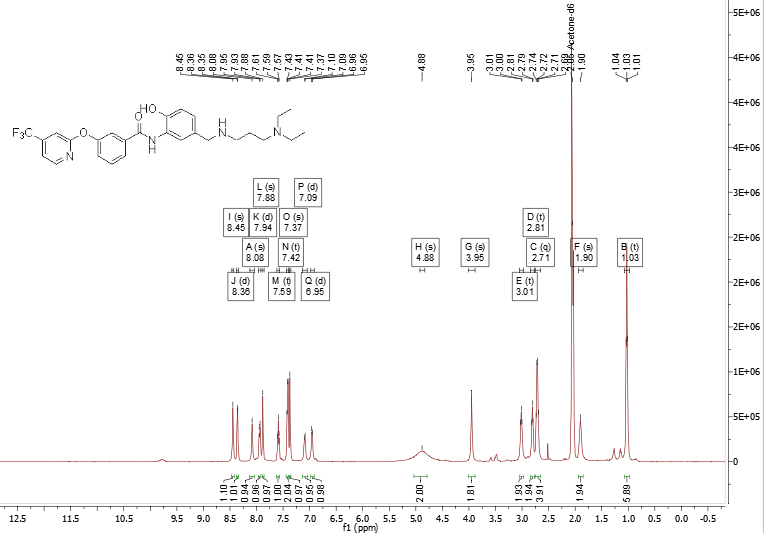
**

**
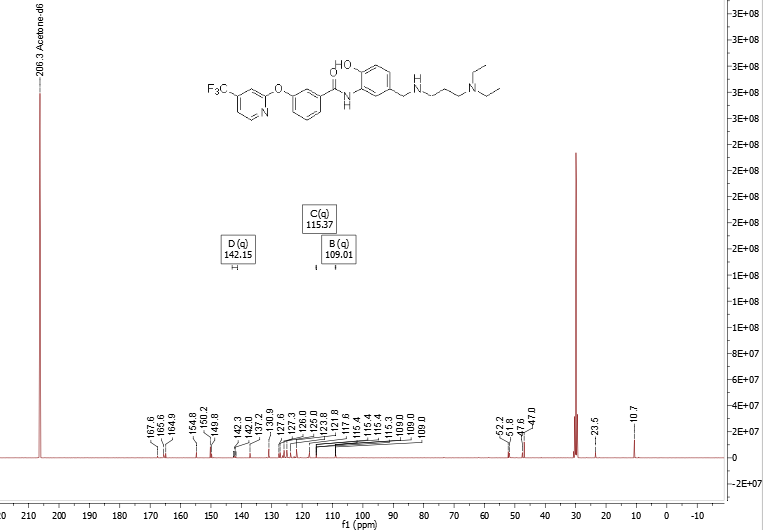
**

**Figure S34: ^1^H-NMR, ^13^C-NMR, HRMS analysis of compound 29**

**
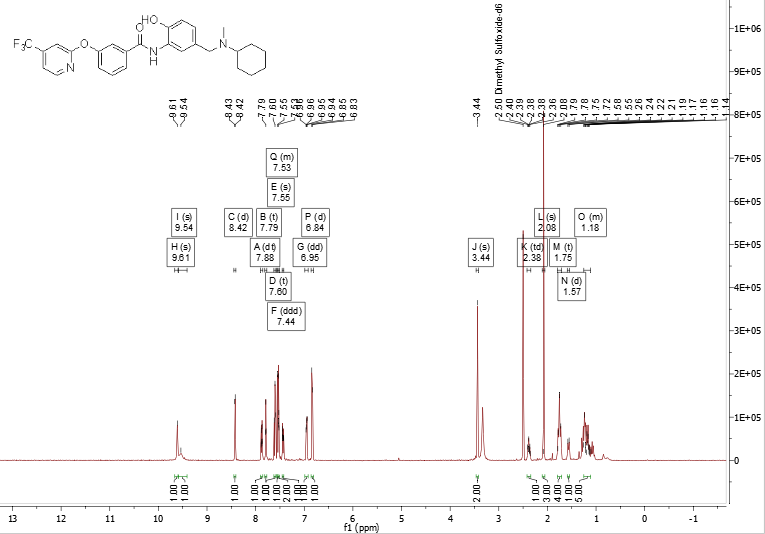
**

**
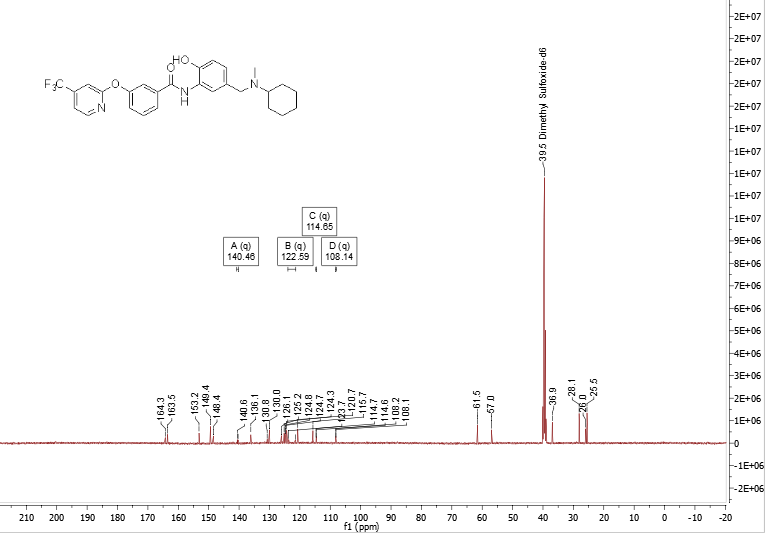
**

**Figure S35: ^1^H-NMR, ^13^C-NMR, HRMS analysis of compound 30**

**
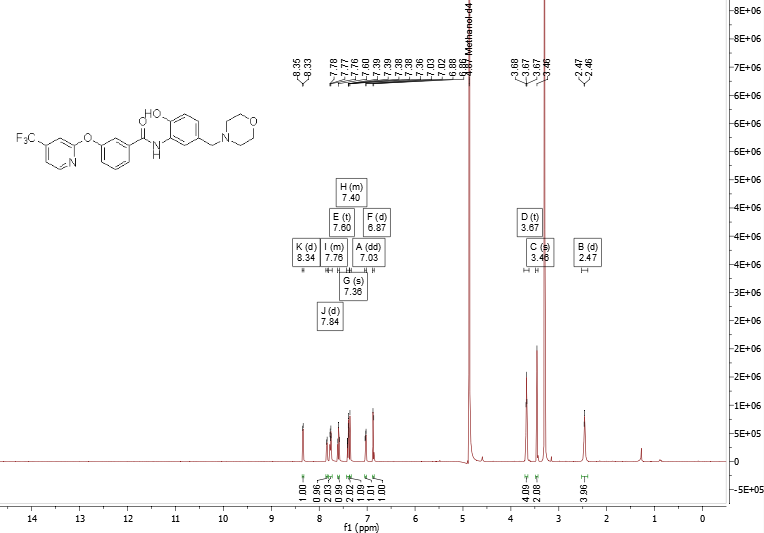
**

**
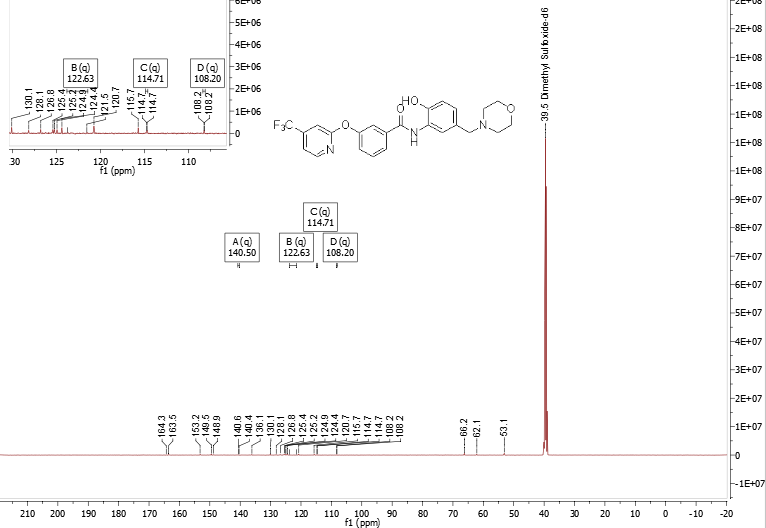
**

**Figure S36: ^1^H-NMR, ^13^C-NMR, HRMS analysis of compound 31**

**
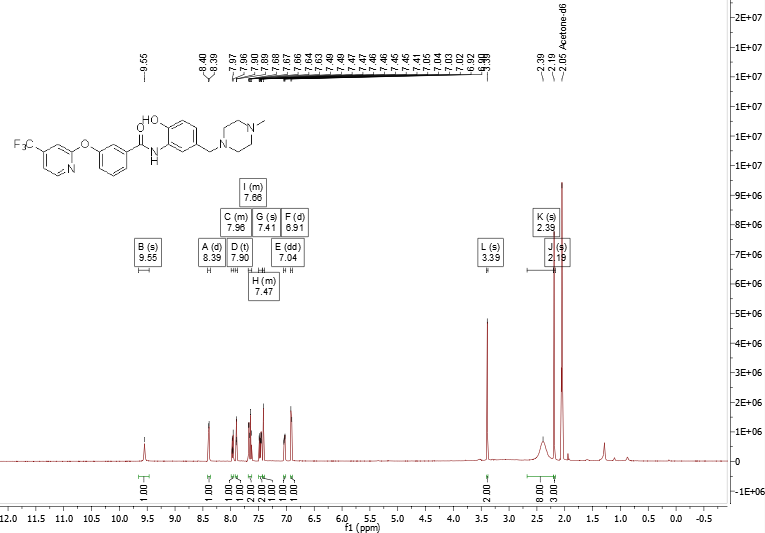
**

**
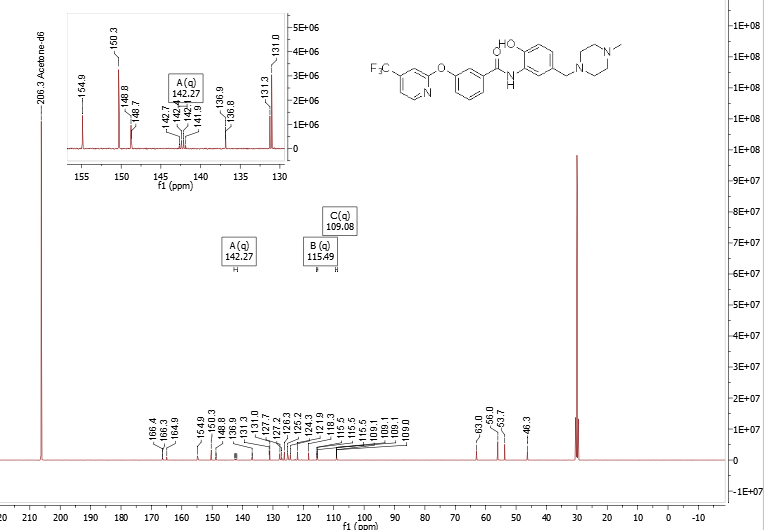
**

**Figure S37: ^1^H-NMR, ^13^C-NMR, HRMS analysis of compound 32**

**
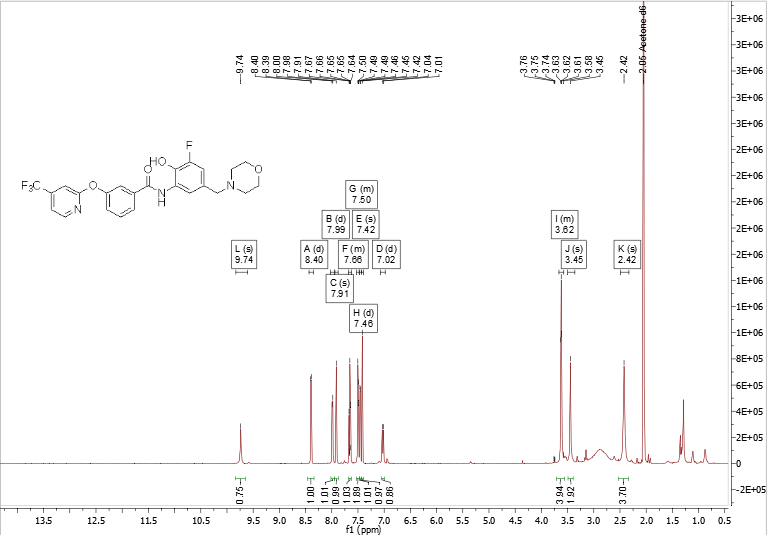
**

**
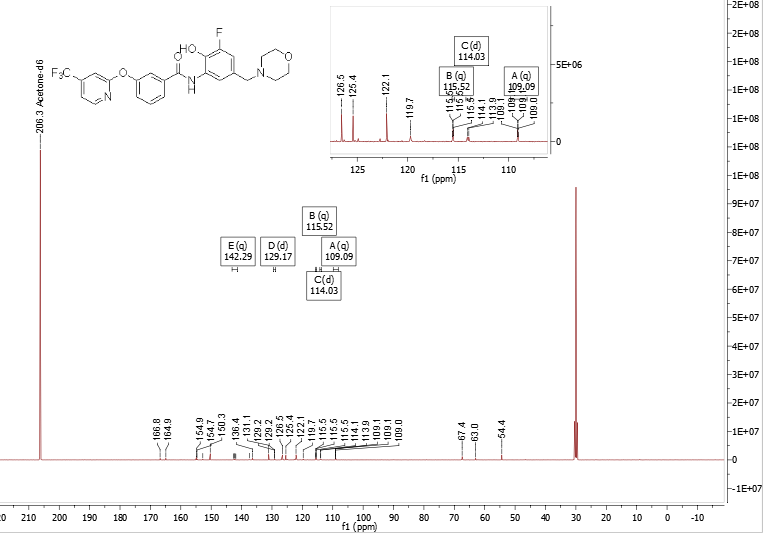
**

**Figure S38: ^1^H-NMR, ^13^C-NMR, HRMS analysis of compound 33**

**
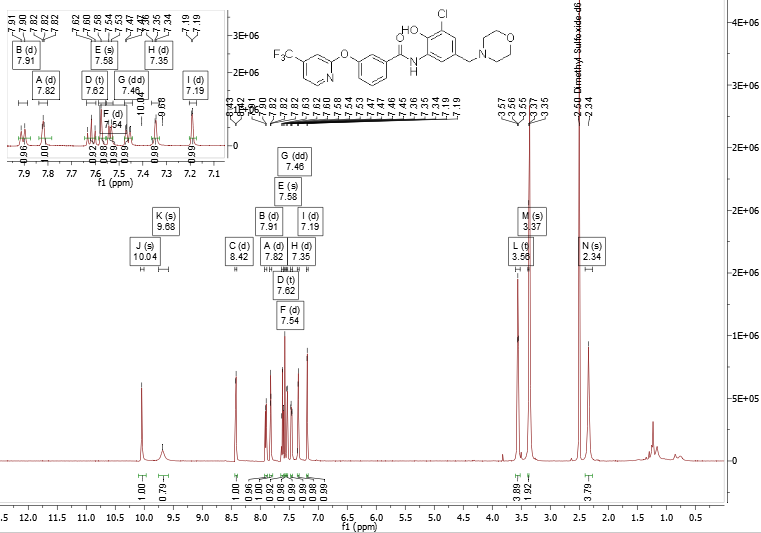
**

**
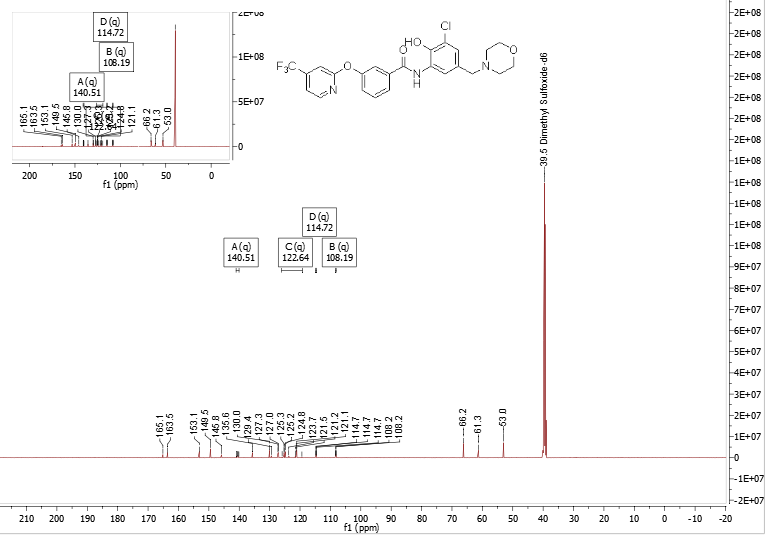
**

**Figure S39: ^1^H-NMR, ^13^C-NMR, HRMS analysis of compound 34**

**
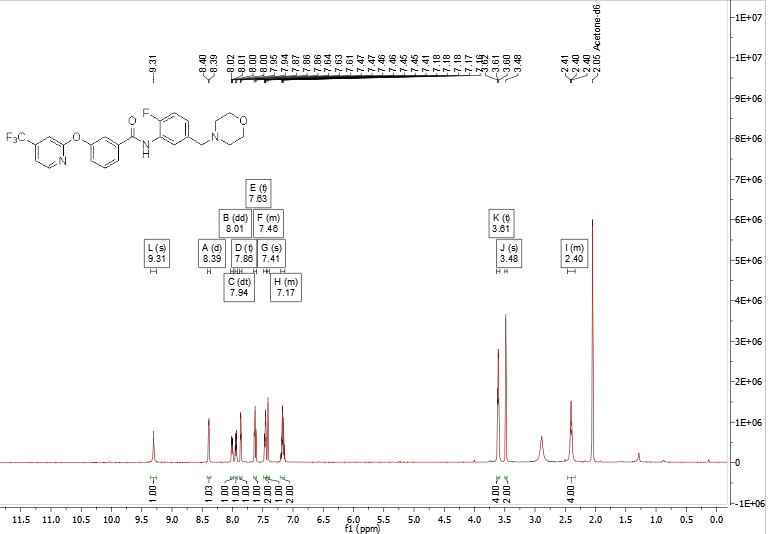
**

**
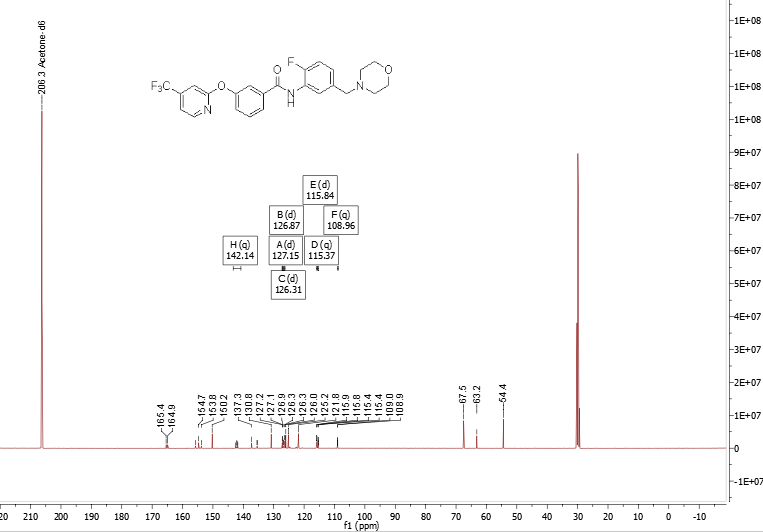
**

**Figure S40: ^1^H-NMR, ^13^C-NMR, HRMS analysis of compound 35**

**
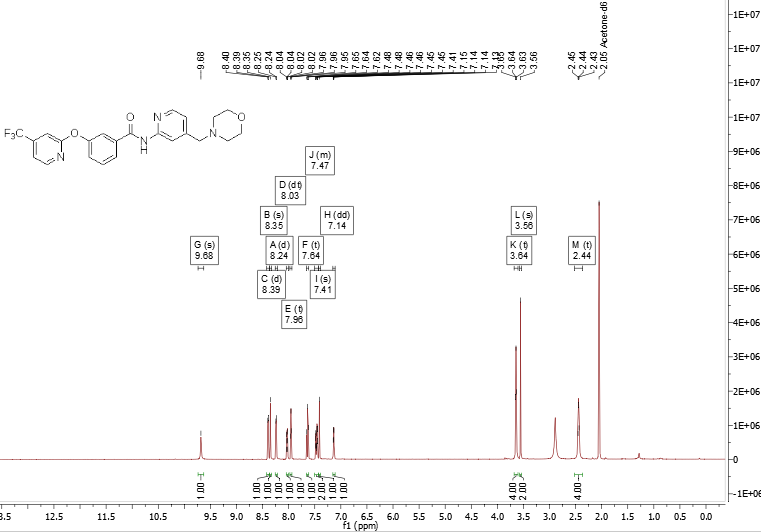
**

**
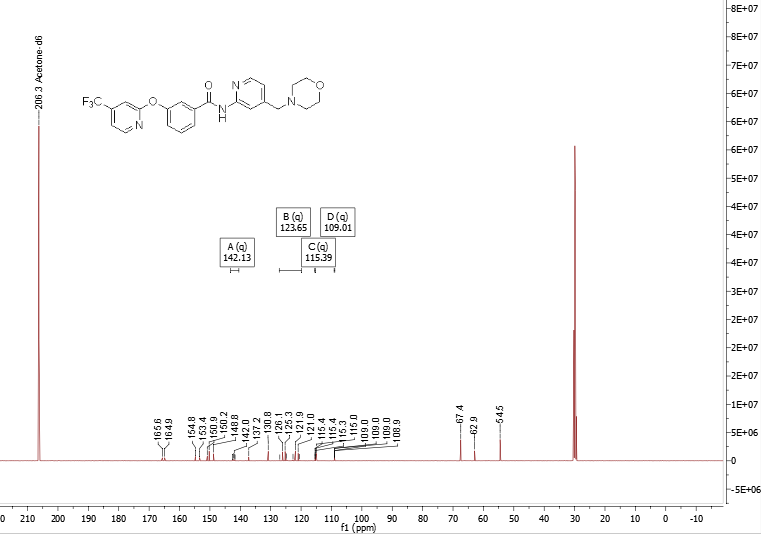
**

**Figure S41: ^1^H-NMR, ^13^C-NMR, HRMS analysis of compound 36**

**
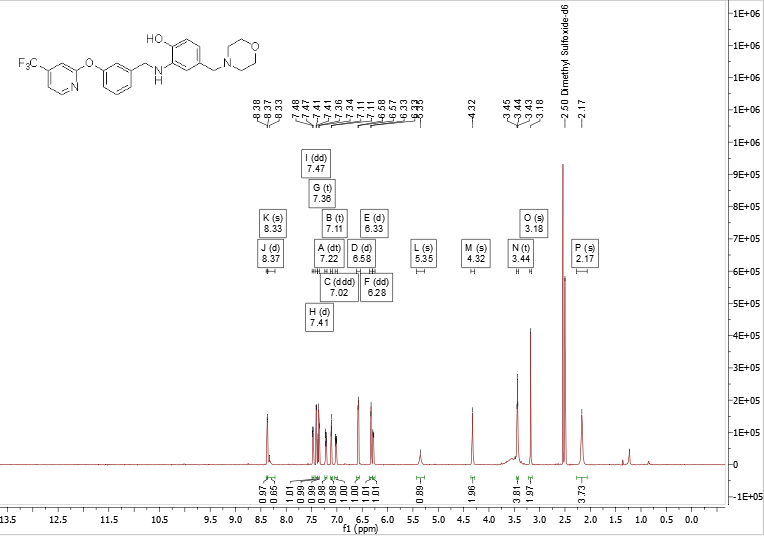
**

**
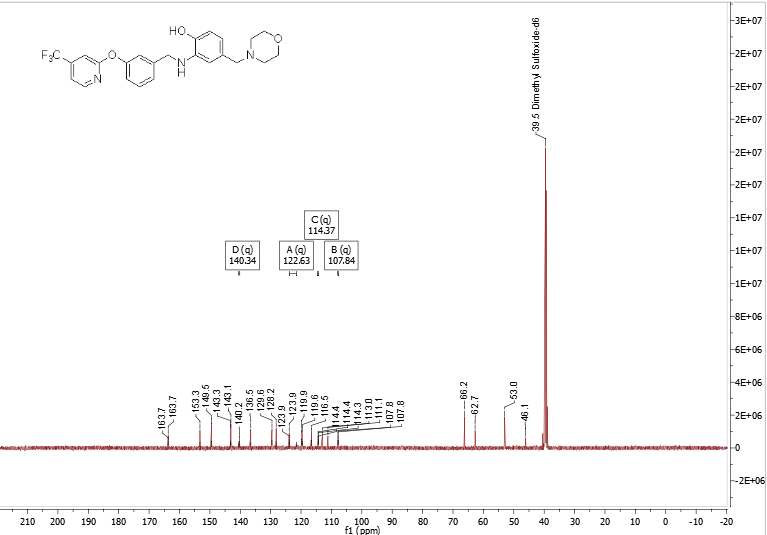
**

**Figure S42: ^1^H-NMR, ^13^C-NMR, HRMS analysis of compound 37**

**
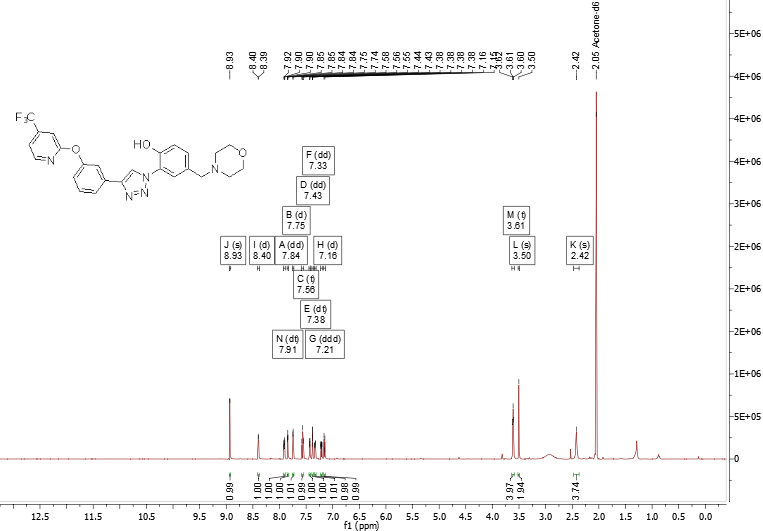
**

**
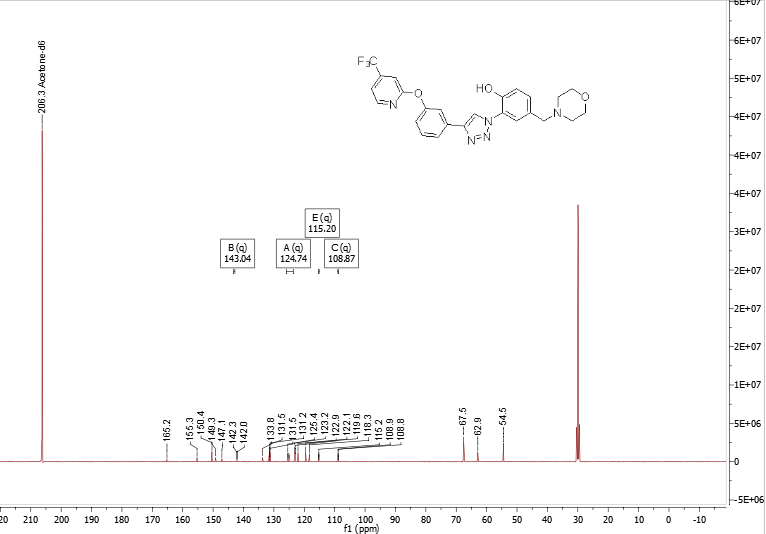
**

# Biological Testing

## Materials

Compounds were synthesized by the following institutions: Helmholtz Institute for Pharmaceutical Research (HIPS, Saarbrücken, Germany) and BASF SE (Ludwigshafen, Germany). The compounds were received as powder and dissolved with dimethyl sulfoxide (DMSO) to obtain 10 mM stock solutions. Control compounds used were purchased from the following: Artesunate (MedChemExpress HY-N0193), Chloroquine (Sigma #C6628), Mefloquine (Sigma #PHR1705), Lumefantrine (AK Scientific, Inc #82186-77-4), Amodiaquine (Sigma #A2799), Pyrimethamine (MedChemExpress, #HY-18062), and Cycloheximide (MedChemExpress #HY-12320). All compounds were prepared as 10 mM stock solutions in 100% DMSO (chloroquine was prepared in ddH_2_0).

## Cytotoxicity assays

HepG2 (2x10^4^ cells per well) were seeded in 96-well flat-bottom culture plates in 100 µL of culture medium (DMEM containing 10% fetal calve serum and 1% penicillin-streptomycin). After 24 hours, the medium was replaced by fresh medium containing test compounds in a final DMSO concentration of 1%. Compounds were tested in duplicate at a single concentration or, for CC_50_ determination, at 8 concentrations that were prepared via 2-fold serial dilutions in 1% DMSO/medium. Epirubicin and doxorubicin were used as positive controls in serial dilutions starting from 10 µM, and rifampicin was used as a negative control (at 100 µM). Cell viability was determined 48 h after treatment with compounds by adding 0.1 volumes of 3-(4,5-dimethylthiazol-2-yl)-2,5-diphenyltetrazolium bromide (MTT) solution (5 mg/mL sterile PBS) (Sigma, St. Louis, MO) to the wells. After incubating the cells for 30 min at 37 °C (atmosphere containing 5% CO_2_), medium was removed and MTT crystals were dissolved in 75 µL of a solution containing 10% SDS and 0.5% acetic acid in DMSO. The optical density (OD) of the samples was measured photometrically at 570 nm in a PHERAstar Omega plate reader (BMG labtech, Ortenberg, Germany). Percent viability was calculated by normalizing sample ODs to those of the DMSO controls. Each compound was tested in at least two independent experiments and CC_50_ values were determined using the nonlinear regression function of GraphPad Prism 10 (GraphPad Software, San Diego, CA, USA).

## Metabolic Stability in Liver S9 Fractions

To evaluate phase I and phase II metabolic stability, the compound (1 μM) was incubated at 37 °C for 240 min with 1 mg/mL pooled mouse liver S9 fraction (Xenotech, Kansas City, USA), 2 mM NADPH, 1 mM UDPGA, 10 mM MgCl_2_, 5 mM GSH and 0.1 mM PAPS. Metabolic stability of reference compounds: testosterone, verapamil and ketoconazole was determined in parallel to confirm the enzymatic activity of mouse S9 fractions. At defined time points, the incubation was stopped by precipitation of aliquots of S9 enzymes with 2 volumes of cold acetonitrile containing internal standard (150 nM diphenhydramine or 15 nM for Altis Plus measurements). Samples were kept on ice until the end of the incubation, after which precipitated protein was removed by centrifugation (15 min, 4 °C, 4,000 g). The residual concentration of the test compound at the different time points was analyzed by HPLC-MS/MS (Ultimate 3000 coupled to TSQ Quantum Access MAX, or Vanquish Flex coupled to a TSQ Altis Plus, Thermo Fisher, Dreieich, Germany) to calculate the half-life (t½).

## *In vitro* *P. falciparum* cell culture and [^3^H]-hypoxanthine incorporation assay

*P. falciparum* asexual blood stage parasites were cultured in human red blood cells (RBC, Blutspende SRK, Zurich) at a hematocrit of 5% in RPMI 1640 medium. The RPMI 1640 medium (ThermoFisher Scientific #51800−043) was supplemented with the following: 24 mM NaHCO_3_ (pH 7.3, Sigma-Aldrich #31437−500G-R), 25 mM Hepes (Sigma-Aldrich #H4034−500G), 0.36 mM hypoxanthine (Sigma-Aldrich # H9377−25G), 100 µg mL^-1^ neomycin (Sigma-Aldrich #N6386−100G) and 0.5% Albumax II (Thermo Fisher #11021−045). Additionally, 2 mM choline chloride (279 mg/L, Sigma-Aldrich #C7527−100G) was added to the culture to block the induction of sexual commitment as described. ^[31]^ The RPMI 1640 medium and components were dissolved in water (Milli-Q purified), stirred for at least 3 h, and then sterile filtered (0.22 μm pore size) with a bottle top filter (Corning # 431118). Parasite cultures were maintained in an atmosphere of 3% O_2_, 4% CO_2_, and 93% N_2_ in air-tight chambers at 37 °C. The [^3^H]-hypoxanthine incorporation assay was used to test compound activity in the specific parasite lines as described.^[32]^ Briefly, compounds were dissolved in DMSO (10 mM) and diluted in a hypoxanthine-free culture medium. A limiting dilution was performed over a 64-fold range (6-step twofold dilution) in a 96-well plate in duplicates. For every starting concentration, a limiting dilution was performed over a 64-fold range (6-step twofold dilution) in a 96-well plate in two biological duplicates. Parasites were added at 100 µL to each well containing the compound to obtain a final parasitemia of 0.3% and haematocrit of 1.25%. The plates were incubated for 48 h before 0.25 µCi of [^3^H]-hypoxanthine was added per well for an additional 24 h. Parasites were harvested on a glass-fiber filter with a Microbeta FilterMate cell harvester (Perkin Elmer, Waltham, USA) and radioactivity was counted using a MicroBeta2 liquid scintillation counter (Perkin Elmer, Waltham, US). The results were recorded, processed by subtraction of the mean background signal obtained from the uninfected RBC controls and expressed as percentage of untreated controls. The fifty percent inhibitory concentration (IC_50_) was calculated by using nonlinear, four parameter (variable slope) curve fitting as described ^[33]^ after importing into GraphPad Prism (version 8.3.0).

## Resistance selection

**31** was used for resistance selection studies using the strain Dd2b2 (kindly provided by David Fidock). Resistance selection was done as described in ^[34]^. As summarized in Table S3, an initial inoculum of 2 x 10^9^ asexual blood stage parasites (at 2-2.5% parasitemia) were cultured in three independent flasks. Each flask was exposed to 3xIC_50_ (58.4 nM, Day 1-5), increased to 5xIC_50_ (97.3 nM, Day 7-11) and then maintained at 7xIC_50_ (136.2 nM) until recrudescence was observed. Cloning via limiting dilution was performed to obtain resistant clones for further characterization as described in section 2.2.5. Drug susceptibility experiments were done as described in section 2.2.3 to confirm the resistant phenotype.

## Cloning via Limiting Dilution

Cloning through limiting dilution was performed as described.^[35]^ Briefly, asynchronous parasite cultures were diluted with fresh RPMI 1640 culture media (with supplements described in section 2.2.3) and RBCs to a parasitemia of 0.0006% (parasite cell suspension) and a haematocrit of 0.75%. Into each well of row A, 100 µL parasite cell suspension was added and 200 µL RBC suspension (culture media/0.75% haematocrit) resulting in a 0.0002% parasitemia (approximately 30 parasites per well, 1/3 dilution). Next, 100 µL from row A suspension was pipetted into row B resulting in a 1/3 dilution (approximately 10 parasites/well). Serial dilutions were continued until the row H and the microplate was incubated at 37^o^C with mixed gas containing 3% O_2_, 4% CO_2_, and 93% N_2_ in an airtight container under atmospheric pressure for 14 days without medium change. Thereafter, the 96-well microplate was imaged to visualize plaques in the RBC (Perfection V750 Pro scanner, Epson, Nagano, Japan). Wells with single plaques were selected and contents were transferred to individual cells of 6-well culture plates with 5 mL fresh culture media and RBCs (5% haematocrit). Parasite growth was monitored with blood smears performed every 2 days. Cultures were monitored until 1% parasitemia was reached and subsequently transferred to a 10-mL culture dish.

## Illumina Whole Genome Sequencing Analysis and Sanger Sequencing

The gDNA of the Dd2b2 **31** selected clones (R2.1, R3.1 and R3.2) and the Dd2b2^WT^ (from day 0 of the start of resistance selection) was isolated using the QIAamp DNA Blood kit mini. The DNA sequencing libraries were prepared using the PCR-free KAPA HyperPrep Kit (Roche) to avoid amplification bias due to the high AT-content of *P.falciparum* gDNA. Libraries were sequenced with the Illumina NextSeq 500 with a read length of 2 x 150 bp and the WGS was analyzed as described in ^[36]^. Briefly, the quality of raw reads was analyzed with FastQC (version 0.11.4). ^[37]^ The raw reads were trimmed with Trimmomatic (version 0.39.11).^[38]^ The *Plasmodium falciparum* 3D7 reference genome (PlasmoDB version 57) ^[40]^ was then indexed using the Burrows-Wheeler Aligner (BWA) (version 0.7.17). ^[39]^ The FASTQ files were mapped to the indexed *Plasmodium falciparum* 3D7 reference genome. The aligned SAM files were converted to BAM files using SAMtools (version 1.7).^[41]^ Using Picard (version 2.6.0), the BAM files were coordinate sorted and indexed. ^[42]^ The read groups were added and duplicate reads were marked. Base quality scores were recalibrated using GATK BaseRecalibrator using known variant sites from the MalariaGEN Pf3k (version 4.2) project. ^[43]^ The variants (SNP and InDels) were called using the Genome Analysis Toolkit HaplotypeCaller (GATK version 4.0.7.0) ^[44]^ in GVCF mode to do a multisample variant analysis wherein the corresponding g.vcf file was combined and genotyped using GATK GenotypeGVCF (GATK version 4.0.7.0).^[44]^ The corresponding variants were filtered using GATK VariantFiltration (GATK version 4.0.3.0) filtering out SNPs and InDels that did not meet the standard criteria set by GATK. Variants were annotated using SnpEff (version 5.0).^[45]^ Around 150`000 variants were called and downstream filtering was applied to remove false positives. Variants fulfilling the following criteria were passed on for downstream analysis: 1) variants that “PASS” the default criteria set by GATK VariantFiltration; 2) variants predicted by SnpEff to have a “HIGH” or “MODERATE” impact these would be variants resulting in protein truncation, nonsense-mediated decay (i.e. stop gained or frameshift *variant*), or other changes of the amino acid sequence (i.e. missense variant or inframe deletion); 3) variants that are absent in the parent clone (Dd2b2^WT^); 4) variants with a phred-scaled probability that a given reference or alternative allele is present on the given gene location (“QUAL > 500”); 5) variants with an assigned genotype quality of 99 for the parent clone. The 59 variants that pass the downstream filtering criteria were visually scanned with the Integrative Genomics Viewer (IGV, version 2.5.0). ^[46]^ Variants were deemed as false positives when they were: 1) supported only by a small number of reads or 2) present in reads from the parent clone and not called due to low allele frequency. Mutations in PF3D7_0523000 and PF3D7_0305500 passed all criteria and were identified as potential alterations causing reduced susceptibility to **31**. Sanger sequencing (Microsynth AG), after PCR amplification of the target regions (primers listed in Table S11: Primers 1,2 for *pfmdr1* G182(S), primers 3,4 for *pfmdr1* K1065(N), and primers 5, 6 for *pfdopey* N3449 (dup)) was carried out to confirm the variants identified with WGS. Sanger sequencing data analysis and visualization was performed using SnapGene software (version 5.1.2). Pymol (v 2.6.0) was used to study the localization of mutated amino acids identified in the PfMDR1 protein (PDB: 8jvh).^[47]^

## Transfection construction

The CRISPR/Cas9 gene editing system is based on the cotransfection of a donor and a Cas9 plasmid as described in ^[18]^. The Cas9 plasmid, referred to as pHF-gC_pfmdr1S182, is derived from the pHF-gC plasmid encoding the Cas9 enzyme, the single guide RNA (sgRNA) cassette, and the human dihydrofolate reductase (hDHFR; conferring resistance to WR99210) resistance marker fused to the negative selection marker yeast cytosine deaminase/uridyl phosphoribosyl transferase (hDHFR-yFCU) ^[18]^. The mother plasmid pHF-gC is described in.^[18]^ The Cas9 endonuclease is expressed from the 5` heat shock protein (hsp) 86 promoter. ^[18]^ The sgRNA was expressed from the U6 snRNA polymerase III promoter (5` U6). ^[18]^ The sgRNA sequences were selected using ChopChop, an online sgRNA design tool, and was selected based on the proximity within the region of interest, GC content and absence of polymeric AT regions. ^[48]^ Moreover, each sgRNA sequence selected was ran on EuPaGDT, an online design tool to check for potential off-target hits ^[49]^ and the efficiency score based on Doensch.^[50]^ Two sgRNAs were used to obtain the 3D7^pfmdr1 G182S^ parasite clone. To do this, complimentary oligonucleotides with *BsaI* overhangs (sgRNA1_F and sgRNA1_R to get sgRNA1; sgRNA2_F and sgRNA2_R for sgRNA2) were annealed and the resulting double-stranded fragment was ligated into the *BsaI*-digested pHF-gC plasmid with the T4 DNA ligase generating the pHF-gC_pfmdr1S182 (pHF-gC_pfmdr1S182_sgRNA1 and pHF-gC_pfmdr1S182_sgRNA2).

The donor plasmid, referred to as pD-pUC19_pfmdr1S182, contains the sequence assembly needed for homology-directed repair of the DNA double-strand break induced by the Cas9. This includes a 5` homology box (5` HB, 578 bp) and 3` homology box (3`HB, 785 bp) on either side of a recodonized region (RR, 133 bp) recodonized by including the mutation (G182S) and the silent mutations. To obtain the 3D7^Cas9 pfmdr1 G182S^ parasite line, the donor plasmid (pD-pUC19_pfmdr1S182) was generated with Gibson assembly reactions consisting of three fragments: (1) The 5` HB was amplified as follows. Briefly, fragment 1a was amplified from the gDNA of the 3D7 clone using PCR reaction primers ( primers 1_F and 1_R), fragment 1b was amplified using 1a as a template (primers 1_F and 2_R), fragment 1c was then amplified using 1b as a template (primers 1_F and 3_R) and fragment 1c was then used for the Gibson assembly reactions; (2) Fragment 2 (3` HB) was amplified from the gDNA of the 3D7 line (primers 2_F and 4_R); (3) Fragment 3 was amplified from the pUC19 plasmid (primers 3_F and 5_R). A Gibson assembly reaction was then performed on fragment 1c, fragment 2 and fragment 3 (NEBuilder HiFi DNA assembly Master Mix (NEB #M5520)) to obtain the pD-pUC19_pfmdr1S182 plasmid.

## Transfection and transgenic lines

The *P. falciparum* 3D7 wild type parasites in the ring stage were transfected as described.^[20]^ Infected RBCs collected from a 5 ml culture of synchronous ring stage parasites (5-6% parasitemia at ) were added to 250 µl of packed RBCs and were co-transfected with 50µg of Cas9 and 50 µg of donor plasmids using a Bio-Rad Gene Pulser Xcell Electroporation System (single exponential pulse, 310 V, 250 µF). 24 hours post-transfection, 5 nM WR99210 (MedChemExpress #HY-116387) was added to the culture medium for six days to select for gene-edited parasites, and henceforth parasites were maintained in normal culture medium until a stably propagating parasite population was obtained. After ~4 weeks post-transfection, stably propagating parasite population were obtained. The transgenic parasite line (3D7*^pfmdr1^* ^G182S^) was cloned out using an established plaque assays via limiting dilution.^[35]^ Successful recodonizing of the target region was confirmed by PCR amplification and subsequent sanger sequencing of the gDNA from the transfected clone (primer 1 and 2, Table S11).

## Surface sensing of translation (SUnSET) and Western Blotting

SUnSET: The SUnSET assay was done as described in. ^[27]^Tightly synchronized NF54 wild type parasites were treated with the compounds for 4h at 30-35 hpi (trophozoite stage). The following compounds and amounts used were: cycloheximide (3xIC_50_, 1,2 µM; Calbiochem), pyrimethamine (3xIC_50_, 21 nM; MedChemExpress), **34** (3xIC_50_, 12 µM), and **31** (3xIC_50_, 12 nM; 50xIC_50_, 200 nM; 100xIC_50_, 400 nM). Parasites were incubated with 1 µg mL^-1^ (Sigma P8833) for 1 h at 37^o^C and saponin lysis was performed.

Saponin lysis and Western blot: Saponin lysis was done on infected RBC at ~5% parasitemia to lyse erythrocytes. Parasite cultures were centrifuged at 1,200 g for 5 min at room temperature. Once the supernatant was discarded, 10 volumes of ice-cold 0.1% (w/v) saponin (Calbiochem 558255, dissolved in PBS, and filtered with 0,22) was added, incubated on ice for 10 min and centrifuged for 2,272 g for 15 min at 4^o^C. Washing steps were done with ice-cold PBS and centrifuged for 2,272 g for 5 min at 4^o^C until supernatant was transparent and lysed erythrocytes were removed. The parasite pellet was first dissolved in 5% SDS lysis buffer supplemented with cOmplete (EDTA-free) protease inhibitor cocktail (Roche), and 1 mM DTT in 50 mM Tris-HCl (pH 7.5). Next, a 1:1 dilution was done with Laemmli buffer (62.5 mM Tris base, 2% SDS, 10% glycerol and 1 mM DTT) before loading. Proteins were separated on 4-12% Bis-Tris gel (Novex, Qiagen) using MOPS buffer (NOvex, Qiagen). Proteins were transferred into an iBlot^TM^ transfer stack (Thermofisher, Scientific) and was transferred with an iBlot 3 western blot transfer system (Thermofisher, Scientific). Proteins were blocked with 5% milk powder in PBS/0.1% Tween (PBS-T) for 1h. The membrane was probed with primary antibodies mAb mouse α-PfGAPDH (1:20’000) ^[51]^, or mAb mouse α-puromycin (1:5’000, Sigma MABE343) diluted in PBS-T with 0.1% milk powder. After an overnight incubation at 4^o^C, the membrane was washed 3 times in PBS-T (incubated for 3 min in each washing step). The membrane was probed with the secondary antibody goat α-mouse IgG (H&L)-HRP (1:10’000, Invitrogen, #31430), which was diluted in PBS-T with 0.1% milk powder and was incubated for 1h at room temperature. The membrane was washed 3 times in PBS-T (3 min incubation in each washing step) and the signal was detected using the chemiluminescent substrate SuperSignal West Pico Plus (Thermo Scientific, REF 34580) and the imaging system Vilber Fusion FX7 Edge 17.10 SN. Quantification of Western blot signal was done by measuring the integrated density of each lane using ImageJ analysis software (version 1.54p). Background signal was subtracted from each measurement, and the resulting values were normalized to the corresponding GAPDH loading control to obtain normalized signal intensities.

## Dose-response assay on gametocytes using the MitoTracker-based readout

Sexual commitment was induced in the NF54/iGP1_RE9H^ulg8^ parasite line as described in ^[18,52]^. Synchronous ring stage cultures at 2-3% parasitemia (8-16 hpi) were grown for 48 hours in a culture medium lacking D-(+)-glucosamine hydrochloride (GlcN) and containing 1.25 μM Shield-1 to induce GDV1-GFP-DD expression. The induction medium was replaced with standard culture medium containing 50 mM N-acetyl-glucosamine (GlcNAc) for six consecutive days.) to selectively kill asexual parasites.^[53,54]^ Gametocytes were maintained in standard culture medium from day 7 onwards. During the first seven days of gametocyte development, the culture medium was exchanged daily and every second day subsequently. Visual inspection of Hemacolor-stained thin blood smears with a 100x immersion oil objective (total magnification = 1000x) was performed to assess gametocytemia.

Dose response assays were performed in biological triplicates, each in technical duplicate, as described in ^[18, 55]^. Synchronous NF54/iGP1_RE9H^ulg8^ gametocyte cultures were resuspended at ~2% gametocytemia in an assay medium at 3% haematocrit. 75 μl gametocyte suspension (~450’000 gametocytes/well) was pipetted into each well of a 96-well cell culture plate, pre-loaded with 75 μl compound serially diluted in assay medium (11-step/12-step dilution series; 3-fold/4-fold serial dilutions). Gametocytes were exposed to the compounds for 72 hours under standard culture conditions. MitoTracker-based gametocyte viability was quantified using a modified version of published protocols.^[56,57]^ From each well of the assay plate, 36 μl compound-treated gametocyte suspension was transferred to the wells of a cell culture plate preloaded with 4 μl PBS containing 50 μg/ml Hoechst 33342 (Thermo Fisher Scientific; t# H3570) and 5 μM MitoTracker Red CMXRos (Invitrogen;# M46752). After a 2-hour incubation step at 37 ^o^C, 8 μl of the stained gametocyte suspensions were transferred to the wells of a clear-bottom 96-well high content imaging plate (Greiner; #655090) preloaded with 192 μl where a final haematocrit of 0.1% was reached. The cells were allowed to settle for 20 min before image acquisition using the ImageXpress Micro XLS widefield high content screening system (Molecular Devices) in combination with the MetaXpress software (version 6.5.4.532, Molecular Devices) and a Sola SE solid state white light engine (Lumencor). Filtersets for the detection of Hoechst (Ex: 377/50 nm, Em: 447/60 nm) and MitoTracker signals (Ex: 543/22 nm, Em: 593/40 nm) were used with exposure times of 80 ms and 100 ms, respectively. Thirty-six sites per well were imaged using a Plan-Apochromat 40x objective (Molecular Devices, #1-6300-0297). Automated image analysis was performed using the MetaXpress software (see Supplementary Methods) to determine the number of viable gametocytes/well (1294 - 3572 cells/well imaged for untreated control samples). After imaging two technical replicates, the results were averaged and normalized to the plateau of sub-lethal compound concentrations using the “first mean in each data set” function and IC_50_ values were calculated using nonlinear, four parameter (variable slope) curve fitting in GraphPad Prism (version 8.3.0).

## Statistical Analysis

Data were expressed as mean ±s.d. Statistical analysis was done using a Student`s *t*-test using GraphPad Prism (v 8.3.0).

# Proteomics

## *P. falciparum* lysate extraction

*P. falciparum* NF54 lysates were extracted as described in ^[26]^ . The parasite asynchronous culture was extracted once 3-5% parasitemia was reached. Saponin lysis was done as described in 2.2.10. Once the parasite pellet was obtained, 2.5 volumes of ice-cold lysis buffer (50 mM Tris/HCl pH 7.5 (Avantor), 5% glycerol (Sigma-Aldrich), 150 mM NaCl (Carl Roth), 1.5 mM MgCl2 (Sigma-Aldrich), 1 mM DTT (Carl Roth), 0.8% IGEPAL CA-630 (Sigma-Aldrich), 1X Halt Protease and Phosphatase Inhibitor-Cocktails EDTA-free (Thermo Fisher Scientific)) was added to the parasite pellet. The lysate solution was subjected to three cycles of flash freeze-thaw with liquid nitrogen and ddH_2_O at RT. The lysates were centrifuged at 20,000 g for 20 minutes at 4^o^C. The soluble protein fraction (supernatant) was collected and the protein content was quantified using the BCA assay (Pierce™ BCA Protein Assay Kit, Thermo Fisher Scientific). The lysate was stored at -80^o^C until further use.

## iSPP profiling

The *P. falciparum* lysate was thawed on ice and diluted to 0.8 mg/mL. The lysate was aliquoted, and each aliquot was incubated with either a vehicle control (DMSO), **34** (dissolved in DMSO), or varying concentrations of **31** (dissolved in DMSO) at 0.01 µM, 0.1 µM, 10 µM, 50 µM, and 100 µM. Subsequently, the samples were incubated at room temperature for 30 minutes using a rotating mixer. After distribution into a 96-well plate, the samples were treated with increasing concentrations of A.E.A. (v/v) ranging from 14% to 28% (14, 16, 18, 20, 22, 24, 26, and 28%). Following a 20-minute incubation at 37°C with shaking at 750 rpm, the samples were centrifuged at 4400 g for 35 minutes to remove precipitated proteins. The soluble fractions were then pooled in equal volumes into a single sample using the Bravo Automated Liquid Handling Platform. The pooled samples were subsequently prepared for LC-MS/MS analysis.

## Sample preparation for LC-MS/MS analysis

The soluble fractions were dried down and resuspended to a final protein concentration of 1.0 mg/mL in 5% SDS containing 50 mM TEAB, pH 7.5. To reduce disulfide bonds, 10 mM DTT was added to all samples, followed by incubation at 35°C for 30 minutes at 700 rpm (ThermoMixer). For protein alkylation, 55 mM chloroacetamide (CAA, Merck) was added, and samples were incubated for 30 minutes at room temperature in the dark. The samples were acidified with phosphoric acid to a final concentration of 2.5% and diluted sevenfold with 90% methanol in 100 mM TEAB, pH 7.5. The samples were transferred to an S-trap column (Protifi) and subjected to five wash cycles using the same buffer. Sequencing Grade Modified Trypsin (Promega) in TEAB, pH 8.5, was added to the S-trap column at a ratio of 1:10 (trypsin/protein), and digestion was carried out overnight at 37°C. Peptides were eluted sequentially with 50 mM TEAB, pH 8.5, 0.1% formic acid (FA, Th. Geyer), and 50/50 acetonitrile (ACN, SigmaAldrich)/water with 0.1% FA. After drying, peptides were resuspended in 0.5% FA. The peptides underwent desalting on the Bravo Automated Liquid Handling Platform using C18 cartridges (5 μL bed volume, Agilent) following the standard AssayMAP peptide cleanup v2.0 protocol. The C18 cartridges were primed with 100 μL of 50/50 ACN/water with 0.1% FA and equilibrated with 50 μL of 0.1% FA at a flow rate of 10 μL/min. Samples were loaded at 5 μL/min, followed by an internal cartridge wash with 0.1% FA at 10 μL/min. Peptides were eluted with 50 μL of 60/40 ACN/water with 0.1% FA at a flow rate of 5 μL/min. The eluted samples were dried and stored at −80°C until further use.

## Liquid Chromatography and mass spectrometry data acquisition

The samples were solubilized in 0.1% FA and injected in a volume equating to 1 μg into a Dionex UltiMate 3000 nano System (Thermo Fisher Scientific), coupled online to a Q Exactive Plus (Thermo Fisher Scientific) equipped with an Orbitrap mass analyzer. Peptides were delivered to a trap column (75 μm × 2 cm), packed in-house with ReproSil-Pur 120 ODS-3 resin (Dr. Maisch). The separation was performed on an analytical column (75 μm × 55 cm), also packed in-house with Reprosil-Gold 120 C18, 3 μm resin (Dr. Maisch). The flow rate was set at 300 nL/min using a 100-minute gradient ranging from 2% to 32% solvent B (0.1% FA, 5% DMSO in acetonitrile) in solvent A (0.1% FA, 5% DMSO in HPLC-grade water), with the column oven temperature maintained at 50 °C. The Q Exactive Plus instrument operated in data-independent acquisition (DIA) mode with positive ionization. Full scan spectra (m/z 400−1000) were acquired in centroid mode at an Orbitrap resolution of 70,000, with an AGC target set to 3e6 and a maximum injection time of 20 ms. DIA scans were collected using 30 windows with a 1 Da window overlap. HCD collision energy was set to 27%, with a loop count of 30, an Orbitrap resolution of 35,000, an AGC target set to 3e6, and a maximum injection time set to automatic.

## Peptide and protein identification and quantification

The raw LFQ-DIA files were processed using DIA-NN (v. 18.1) in library-free mode, utilizing the UniProt FASTA file for *P. falciparum* NF54 (taxon identifier: 5843). The raw files were digested with Trypsin/P, allowing for a maximum of two missed cleavages. Peptide lengths were restricted to 7-30 amino acids, and the precursor m/z range was set from 300 to 1800. Cysteine carbamidomethylation was specified as a fixed modification, while methionine oxidation and N-terminal acetylation were included as variable modifications. The maximum number of variable modifications was set to three, and the 'match between runs' functionality was enabled. All other parameters were kept at default settings, including a precursor FDR of 1%.

## iSPP profiling data analysis

The raw LFQ-DIA intensity values of biological replicates across all conditions were normalized to the median abundance using Excel. The normalized values were then log2 transformed in Perseus (v2.0.10.0).^[58]^ Missing values were imputed from a normal distribution (width 0.3, down shift 1.5), and p-values were obtained using a two-sample t-test over replicates with a permutation-based false discovery rate correction (FDR 0.05). Volcano plots were generated in RStudio using the EnhancedVolcano package (v. 1.20.0), ^[59]^ plotting proteins by statistical significance (y-axis: –Log10 p-value) versus the magnitude of change (x-axis: log2 fold change of protein intensities for each compound condition relative to the vehicle control). The heatmap was generated in Perseus (v2.0.10.0). Dose-dependent curves were generated using normalized protein intensity values, expressed as a ratio to the vehicle control. Plots were created in GraphPad Prism (v8.3.0) using a nonlinear regression model.

# References

[29] E. M. Zdobnov, R. Apweiler, *Bioinformatics* **2001**, *17*, 847–848.

[30] S. G. Valderramos, D. A. Fidock, *Trends Pharmacol Sci* **2006**, *27*, 594–601.

[31] N. M. B. Brancucci, J. P. Gerdt, C. Wang, M. De Niz, N. Philip, S. R. Adapa, M. Zhang, E. Hitz, I. Niederwieser, S. D. Boltryk, M.-C. Laffitte, M. A. Clark, C. Grüring, D. Ravel, A. Blancke Soares, A. Demas, S. Bopp, B. Rubio-Ruiz, A. Conejo-Garcia, D. F. Wirth, E. Gendaszewska-Darmach, M. T. Duraisingh, J. H. Adams, T. S. Voss, A. P. Waters, R. H. Y. Jiang, J. Clardy, M. Marti, *Cell* **2017**, *171*, 1532-1544.e15.

[32] C. Snyder, J. Chollet, J. Santo-Tomas, C. Scheurer, S. Wittlin, *Exp Parasitol* **2007**, *115*, 296–300.

[33] W. Huber, J. C. Koella, **1993**.

[34] C. L. Ng, D. A. Fidock, *Malar Control Elimin* **2019**, 123–140.

[35] J. A. Thomas, C. R. Collins, S. Das, F. Hackett, A. Graindorge, D. Bell, E. Deu, M. J. Blackman, *PLoS One* **2016**, *11*, e0157873.

[36] E. Hitz, N. Wiedemar, A. Passecker, B. A. S. Graça, C. Scheurer, S. Wittlin, N. M. B. Brancucci, I. Vakonakis, P. Mäser, T. S. Voss, *PLoS Biol* **2021**, *19*, e3001483.

[37] S.W. Wingett, and S. Andrews, FastQ Screen: A tool for multi-genome mapping and quality control. *F1000Research*, **2018,** *7*, 1338

[38] A. M. Bolger, M. Lohse, B. Usadel, *Bioinformatics* **2014**, *30*, 2114–2120.

[39] H. Li, R. Durbin, *bioinformatics* **2009**, *25*, 1754–1760.

[40] C. Aurrecoechea, J. Brestelli, B. P. Brunk, J. Dommer, S. Fischer, B. Gajria, X. Gao, A. Gingle, G. Grant, O. S. Harb, *Nucleic Acids Res* **2009**, *37*, D539–D543.

[41] H. Li, B. Handsaker, A. Wysoker, T. Fennell, J. Ruan, N. Homer, G. Marth, G. Abecasis, R. Durbin, 1000 Genome Project Data Processing Subgroup, *bioinformatics* **2009**, *25*, 2078–2079.

[42] Broad Institute, “Picard Tools 2017,” **2017**.

[43] Malaria Gen Consortium, “MalariaGEN: Genomic Epidemiology Network,” **2024**.

[44] A. McKenna, M. Hanna, E. Banks, A. Sivachenko, K. Cibulskis, A. Kernytsky, K. Garimella, D. Altshuler, S. Gabriel, M. Daly, *Genome Res* **2010**, *20*, 1297–1303.

[45] P. Cingolani, A. Platts, L. L. Wang, M. Coon, T. Nguyen, L. Wang, S. J. Land, X. Lu, D. M. Ruden, *Fly (Austin)* **2012**, *6*, 80–92.

[46] H. Thorvaldsdóttir, J. T. Robinson, J. P. Mesirov, *Brief Bioinform* **2013**, *14*, 178–192.

[47] W. L. DeLano, *CCP4 Newsl. Protein Crystallogr* **2002**, *40*, 82–92.

[48] T. G. Montague, J. M. Cruz, J. A. Gagnon, G. M. Church, E. Valen, *Nucleic Acids Res* **2014**, *42*, W401–W407.

[49] D. Peng, R. Tarleton, *Microb Genom* **2015**, *1*, e000033.

[50] J. G. Doench, E. Hartenian, D. B. Graham, Z. Tothova, M. Hegde, I. Smith, M. Sullender, B. L. Ebert, R. J. Xavier, D. E. Root, *Nat Biotechnol* **2014**, *32*, 1262–1267.

[51] C. A. Daubenberger, E. J. Tisdale, M. Curcic, D. Diaz, O. Silvie, D. Mazier, W. Eling, B. Bohrmann, H. Matile, G. Pluschke, **2003**.

[52] S. D. Boltryk, A. Passecker, A. Alder, E. Carrington, M. van de Vegte-Bolmer, G.-J. van Gemert, A. van der Starre, H.-P. Beck, R. W. Sauerwein, T. W. A. Kooij, *Nat. Commun.* **2021**, *12*, 4806.

[53] Q. L. Fivelman, L. McRobert, S. Sharp, C. J. Taylor, M. Saeed, C. A. Swales, C. J. Sutherland, D. A. Baker, *Mol Biochem Parasitol* **2007**, *154*, 119–123.

[54] T. Ponnudurai, A. H. W. Lensen, J. Meis, J. H. E. T. Meuwissen, *Parasitology* **1986**, *93*, 263–274.

[55] N. M. B. Brancucci, C. Gumpp, G. J. van Gemert, X. Yu, A. Passecker, F. Nardella, B. T. Thommen, M. Chambon, G. Turcatti, L. Halby, *bioRxiv* **2024**, 2010–2024.

[56] S. Duffy, V. M. Avery, *Malar J* **2013**, *12*, 1–15.

[57] Q. L. Fivelman, L. McRobert, S. Sharp, C. J. Taylor, M. Saeed, C. A. Swales, C. J. Sutherland, D. A. Baker, *Mol Biochem Parasitol* **2007**, *154*, 119–123.

[58] S. Tyanova, J. Cox, *Cancer systems biology: Methods and protocols* **2018**, 133–148.

[59] K. Blighe, S. Rana, M. Lewis, *R package version* **2019**, *1*, 10–18129.
